# Syntheses of 2- and 3-Substituted Morpholine Congeners via Ring Opening of 2-Tosyl-1,2-Oxazetidine

**DOI:** 10.1021/acs.joc.3c00207

**Published:** 2023-04-26

**Authors:** Bálint Kőnig, Gábor Sztanó, Tamás Holczbauer, Tibor Soós

**Affiliations:** † Institute of Organic Chemistry, Research Centre for Natural Sciences, 2 Magyar tudósok körútja, H-1117 Budapest, Hungary; ‡ Hevesy György PhD School of Chemistry, Eötvös Loránd University, 1/A Pázmány Péter sétány, H-1117 Budapest, Hungary; § Centre for Structural Science, Research Centre for Natural Sciences, 2 Magyar tudósok körútja, H-1117 Budapest, Hungary

## Abstract

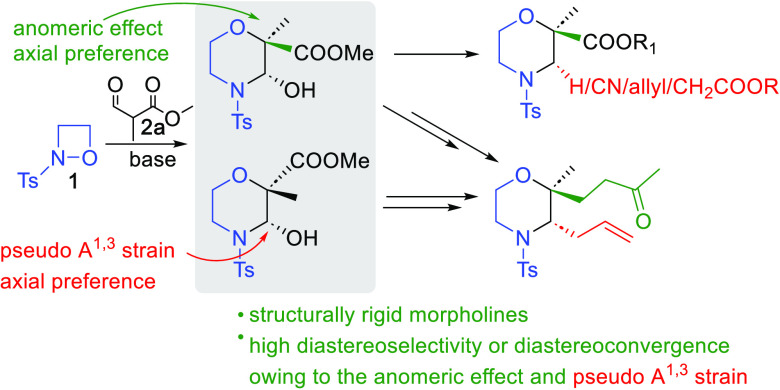

Diastereoselective and diastereoconvergent syntheses of 2- and 3-substituted morpholine congeners are reported. Starting from tosyl-oxazatedine **1** and α-formyl carboxylates **2**, base catalysis is utilized to yield morpholine hemiaminals. Their further synthetic elaborations allowed the concise constructions of conformationally rigid morpholines. The observed diastereoselectivities and the unusual diastereoconvergence in the photoredox radical processes seem to be the direct consequence of the avoidance of pseudo A^1,3^ strain between the C-3 substituent and the N-tosyl group and the anomeric effect of oxygen atoms.

Morpholine and its substituted congeners are among the most extensively utilized heterocycles in medicinal chemistry.^1^ Owing to their reduced p*K*_a_ value, advantageous metabolic property, good solubility, and enhanced blood–brain barrier permeability, these heterocycles have a prominent role in the development of CNS-active compounds.^2^ As a result, many marketed CNS drugs have morpholines as a key structural component (Figure 1). Accordingly, there is a constant need to access diversely substituted morpholines in a rapid and modular manner to modulate the pharmacokinetic properties of morpholine-based ligands. Herein, we disclose the development of a concise and modular synthetic route to polysubstituted morpholines which relies on the ring opening 2-tosyl-1,2-oxazetidine (**1**).

**Figure 1:**
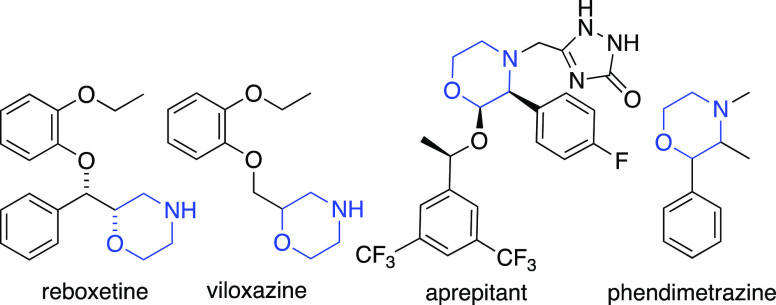
Representative examples of morpholine based drugs.

Given the prevalence and importance of morpholines in drug developments, their syntheses have been extensively studied and various synthetic strategies have evolved.^3−5^ Despite these significant advances, however, the facile access to these valuable molecules is often hampered by the availability, cost, and toxicity of the reagents and the lengthy and inefficient synthetic routes. Additionally, these synthetic routes are mainly based on logical heterolytic disconnections; both oxygen and nitrogen carry a natural negative charge in the disconnected fragments. Recently, 2-tosyl-1,2-oxazetidine (**1**), an umpoled synthon, has emerged as an alternative building block for morpholine ring construction (Figure 2).^6^ Owing to the strongly electron-withdrawing substituents on the nitrogen, the N–O bond is highly polarized and further activated by the ring strain in the four membered ring. Consequently, the oxygen becomes susceptible to nucleophilic attacks and results in the heterolytic cleavage of the N–O bond. First, Orentas reported that this strained heterocycle **1** could react with aromatic organometallic reagents on its electrophilic oxygen providing aminoether derivatives that could be cyclized to benzomorpholines.^6a^ Then, Hu reported the asymmetric synthesis of some fused and spiro morpholines using the 2-nosyl derivative of **1** and indanone carboxylates.^6b^

**Figure 2:**
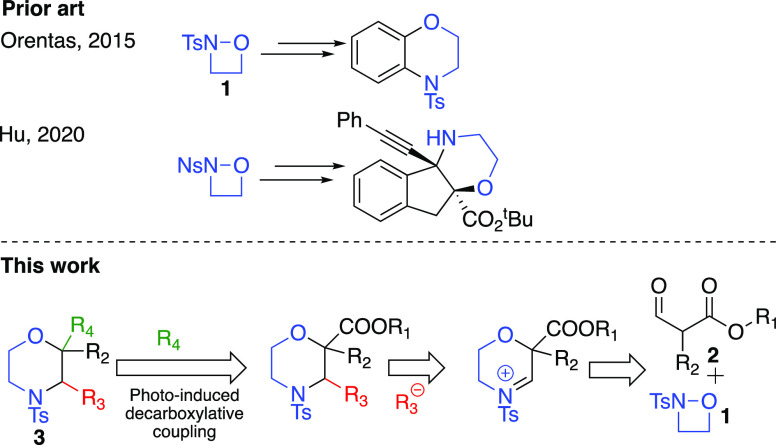
Prior art in oxazetidine chemistry and a retrosynthetic scheme for the preparation of substituted morpholines.

Inspired by the promise of this novel umpoled synthon, we became interested in the exploration of oxazetidine-based synthesis of 2- and/or 3-substituted morpholines. These substitution patterns can introduce constraint in otherwise conformationally flexible morpholines that would be a valuable tool in drug developments.^7^ Thus, we set out to explore a synthetic method toward substituted morpholines using discrete nucleophiles, α-formyl carboxylates **2**, which allow further, divergent synthetic elaborations (Figure 2).

First, α-formyl propionate **2a** was reacted with oxazetidine **1** in the presence of DBU (Table 1, entry 1). Gratifyingly, the applied organic base promoted a cascade sequence to deliver a diastereomeric mixture of morpholine hemiaminal **3a**. Thus, the ring opening of oxazetidine **1** was followed by a spontaneous ring closure. The resulting hemiaminal functionality appeared to be both thermodynamically and kinetically stable, the open chain tautomer was not detected, and the diastereomers could even be separated by column chromatography. Then NOE experiments were used to assign the stereochemistry of both diastereomers (Table 1). Interestingly, both the hydroxy and carboxylate functionalities were in axial positions in the major **3a′** diastereomer to avoid the pseudo A^1,3^ strain^8^ between the hydroxy group and the N-tosyl group, and the stereochemistry at C-2 was influenced by the anomeric effect^9^ of the oxygen atom. It was also established that the hydroxy group resides axially at the C-3 position in **3a″**.^10^ Thus, the pseudo A^1,3^ strain imposed a conformational preference on the morpholine ring of both diastereomers.

**Table 1:**
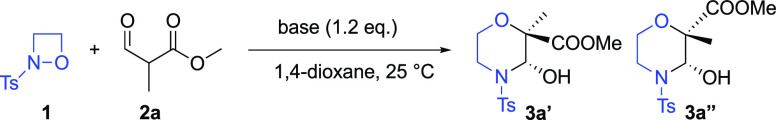
Base Screening to Affect Cascade Reaction of Oxazetidine **1** and α-Formyl Propionate **2a**^a^

entry	base	conversion^d^ (%)	d.r.^e^^,^^f^
**1**	DBU	59	2.9
**2**	TMG	55	2.5
**3**	DBN	45	2.7
**4**	TEA	2	n.d.
**5**	TBD	2	n.d.
**6**	BDMAN^b^	0	–
**7**	DABCO	0	–
**8**	BDCI^c^	33	2.4
**9**	K_2_CO_3_	91	2.0
**10**	K_3_PO_4_	63	2.0
**11**	KOH	53	2.0
**12**	CH_3_COOK	19	2.2
**13**	K_2_HPO_4_	2	2.7

a Reaction conditions: 0.25 mmol substrates **1** and **2a**, 0.30 mmol base in 0.5 mL of 1,4-dioxane at room temperature for 16 h.

b 1,8-Bis(dimethylamino)naphthalene (BDMAN) base was applied.

c (*S*)-(2-((2,3-Bis(dicyclohexylamino)cycloprop-2-en-1-ylidene)amino)-2-phenylethan-1-ol was used as a base.

d Determined by qNMR using 1,3,5-trimethoxybenzene internal standard.

e The diastereomeric ratio (d.r.) was determined by NMR.

f n.d.: not determined.

Then, optimized reaction conditions were established.^10^ In terms of solvent, aprotic, polar, and apolar solvents were viable media for the process, but toluene and 1,4-dioxane proved to be the optimal choice. While variation of concentration had less effect on the yields, the reaction temperature had a more profound impact, as the yield dramatically eroded at high, e.g., 100 °C, temperature. Finally, we screened various organic and inorganic bases to promote the cascade reaction (Table 1). For solubility reasons, we employed 1,4-dioxane as a solvent for this study. As a general trend, we found that strong organic bases could elicit the desired transformation (entries 1–3 vs 4–7). We also tried to promote an enantioselective transformation using a chiral base (2-((2,3-bis(dicyclohexylamino)cycloprop-2-en-1-ylidene)amino)-2-phenylethan-1-ol);^11^ however, no chiral induction was detected (entry 8). Using inorganic bases, the reaction efficiency showed a similar tendency at varying basicity strength (entries 9–11 vs 12, 13). Importantly, K_2_CO_3_ proved to be the most effective base to promote the hemiaminal **3a** formation with a conversion of 91%. The applied organic and inorganic bases, however, had no significant impact on the diastereoselectivity of the reaction that ranged between 2.0–2.9.

With the optimal conditions in hand (Table 1, entry 9), we began to explore the scope of the morpholine ring forming cascade reaction focusing on substituted α-formyl esters. This screening of nucleophiles revealed that these esters are viable substrates for this reaction and afforded the desired ring-closed morpholine products **3b**–**l** (Scheme 1). Importantly, alkyl halogenide (**3h**), multiple bonds (**3d**,**f**), and acetal functionality (**3k**) were all tolerated. In the case of diester **2l**, the cascade reaction spontaneously underwent intramolecular transesterification under the standard conditions, yielding a 6/5 fused ring system **3l**. A further important observation was that highly acidic substrates failed to undergo the desired transformation (**3m**–**p**). Finally, the synthesis of **3a** was conducted on a gram scale without significant change in the yield and diastereoselectivity (Scheme 1).

**Scheme 1:**
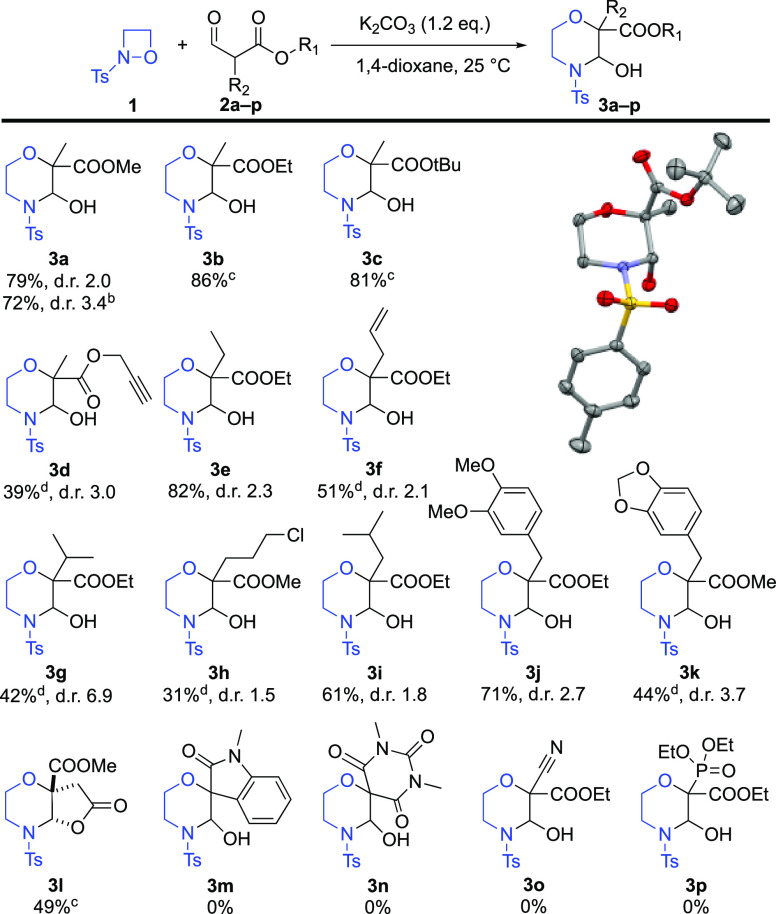
Scope of the Morpholine Ring Forming Cascade Reaction Using α-Formyl Ester **2a**–**p** Nucleophiles and Perspective View of **3c** with 30% Ellipsoid Probability Reaction conditions: 0.25 or 0.30 mmol substrates **1** and **2a**–**p**, 0.30 or 0.36 mmol K_2_CO_3_ in 0.5 mL of 1,4-dioxane at room temperature for overnight, isolated yields, d.r. determined by NMR of the crude product. Gram scale (11.7 mmol) reaction. Single diastereomer. Only the major isomer could be isolated.

When the same protocol was applied in the reaction of oxazetidine **1** and various β-keto and cyano esters **4a**–**f**, only the formation of ring opened **5a**–**f** product was observed (Scheme 2). Thus, the keto functional group was less susceptible to nucleophilic attack by the tosyl-NH group. Additionally, under this condition, no ring-closure of the tosyl-NH to ester or cyano groups was detected. Interestingly, we could modulate the reactivity of α-unsubstituted β-keto-esters (**4d**,**e**) with steric effect. In the case of *t*-Bu derivative **4d**, the reaction halted at the monosubstituted level, and no further reaction occurred (**5d** vs **5e**).

**Scheme 2:**
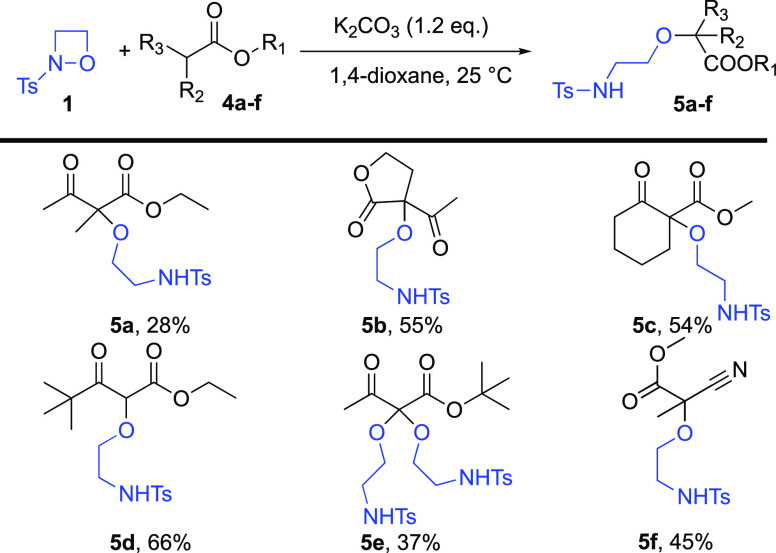
Scope of the Ring-Opening Reaction with Keto and Cyano Carbon Nucleophiles **4a**–**f** Reaction conditions: 0.30 mmol substrates **1** and **4a**–**f**, 0.36 mmol K_2_CO_3_ in 0.5 mL of 1,4-dioxane at room temperature for overnight, isolated yields.

Next, further synthetic elaboration of morpholine hemiaminal was explored to access variously substituted morpholines. First, we probed viable synthetic routes to introduce functionalities at the 3-position of the morpholine scaffold of **3a′** using the hemiaminal functionality as a handle. As shown in Scheme 3, the hemiaminal **3a′** underwent smooth reduction with Et_3_SiH and BF_3_·Et_2_O Lewis acid to afford **6**. As might be expected, the hemiaminal **3a′** could be elaborated to the corresponding cyano derivative **7** using TMSCN. Surprisingly, the reaction proved to be diastereoselective, and X-ray crystallography was used to determine the molecular structure of **7**. This revealed that the substitution was a formal stereoretentive nucleophilic substitution affording a trans product **7** in a conformer which could minimize the pseudo A^1,3^ strain^8^ (N-Ts and CN) and influenced by the anomeric effect of oxygen atoms. Although we could not detect the open chain tautomer of **3a′**, we were delighted that Wittig and Horner–Wittig reactions could be accomplished under mild conditions to yield **8** and its ring closed derivative **9**, respectively. Importantly, a diastereoselective aza-Michael reaction can easily be effected on **8** with either a base or heat to furnish the ring-closed morpholine product **9**. Nevertheless, both methods delivered the same, conformationally rigid diastereomer. Finally, attempts to convert **3a′** in the Hosomi–Sakurai reaction were met with success. However, the reaction was less diastereoselective than previous methods and afforded both possible and chromatographically separable diastereomers, the C-3 allylated **10′** and **10″** products, in a 3.4:1 ratio. Importantly, the Hosomi–Sakurai allylation reaction on the diastereomeric **3a″** yielded the same, consistent result (**10′** and **10″** in 3.4:1 ratio), which suggests that these diastereoconvergent reactions^12^ likely proceeded via the more stable iminium intermediate **11a** (presumably the same putative intermediate toward **7**) in which the ester group prefers a pseudoaxial orientation owing to anomeric effect (**11a** vs **11b**). Additionally, the nucleophilic addition along the axial trajectory from top face (a) is presumed as it gives a lower-energy product via chairlike transition state.^13^

**Scheme 3:**
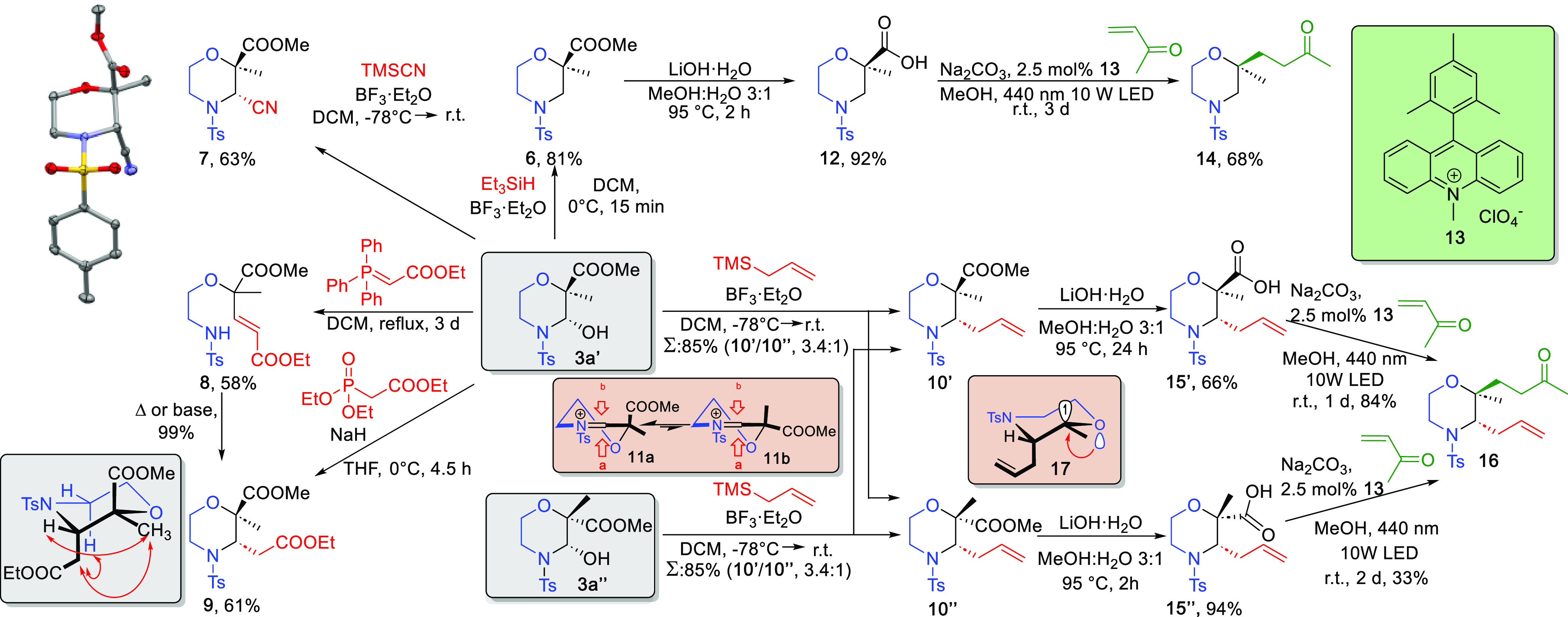
Further Synthetic Elaboration of **3a′** and **3a″** to Access Various 2- and 3-Substituted Morpholines and Possible Intermediates of Stereodivergent Reactions and Perspective View of **7** with 30% Ellipsoid Probability

Having thus developed methodologies to derivatize **3a** and **3a′** at the 3-position, we then sought to advance toward the functionalization at the 2-position using the carboxylate functionality as a radical source. To this end, we considered applying a visible light promoted direct decarboxylative Giese-type reaction.^14^ As outlined in Scheme 3, a two-step route from morpholine derivative **6** was realized using Fukuzumi catalyst **13**. Accordingly, the ester hydrolysis to carboxylic acid derivative **12** was followed by the decarboxylative alkylation under air and room temperature to deliver the desired ketone **14** with good yield. Next, the diastereoselectivity of this radical process was investigated using both allylated products **10′** and **10″**. The cleavage of the ester group of **10′** and **10″** furnished **15′** and **15″**, respectively. As expected, the axial ester **10′** was more resistant to hydrolysis than the equatorial ester **10″** due to 1,3-diaxial strain in **10′** which resulted in a longer reaction time and a diminished yield. Then, the decarboxylative Giese-type reaction was initiated and processed essentially as described for the analogous **12**. Thus, after the generation of radicals, methyl vinyl ketone was introduced into the 2-position. However, instead of formation of diastereomers, both reactions yielded exclusively the same, single diastereomer **16** in a diastereoconvergent manner. Additionally, an anomeric assistance was observed during these homolytic decarboxylations, as the diastereomer **15′** having axial carboxylic group reacted faster and proceeded in higher yield than the equatorially substituted carboxyl isomer **15″**. These results suggest that both stereoelectronic and steric effects (pseudo A^1,3^ strain) govern the reaction,^8,9b,15^ and it proceeds via a common radical intermediate **17**.

In conclusion, we have developed concise synthetic routes to afford elaborated 2- and 3-substituted morpholine congeners. Starting from tosyl-oxazatedine **1** and α-formyl carboxylates **2**, base catalysts is utilized to yield morpholine hemiaminals **3a**–**l**. Using the diastereomeric morpholine hemiaminals **3a′** and **3a″** and relying on selected classical methods and a photoredox mediated Giese-type reaction, we demonstrated that their further synthetic elaborations allowed the concise, diastereoselective, and even diastereoconvergent constructions of highly decorated and conformationally rigid morpholines. The observed diastereoselectivities and the unusual diastereoconvergence in the photoredox radical processes seem to be the direct consequence of stereoelectronic and steric effects. This operationally simple approach can be a valuable addition to morpholine chemistry with a potential to further expand the scaffold diversity.

## Experimental Section

### General Information

All starting materials were used without further purification unless stated otherwise. Anhydrous THF and toluene were distilled from sodium/benzophenone. The light promoted reactions were performed in 4 mL borosilicate vials (5 cm from light source) using 440 nm power LED (Shenzhen Weili Optical Co., Royal Blue 10 W high power LED). Melting points were determined with an SRS MPA100 apparatus and are uncorrected. Exact mass measurements were performed on a high-resolution hybrid quadrupole-time-of-flight mass spectrometer (Waters Select Series IMS, Waters Corp., Wilmslow, U.K.) equipped with a Z-spray electrospray ionization source. Samples were dissolved in acetonitrile–water 1:1 (V/V) solvent mixture containing 0.1% (V/V) formic acid. Solutions were directly introduced into the ion source using a syringe pump. Under the applied conditions, the compounds form protonated, [M + H]^+^, and sodiated, [M + Na]^+^, molecules in positive ionization ESI. X-ray crystallography was performed on a Rigaku RAXIS-RAPID II diffractometer. NMR spectra were recorded on Varian 300 and Varian 500 spectrometers. ^13^C NMR spectra were acquired in broad-band continuous decoupled mode. The chemical shifts (δ) are given in parts per million (ppm) using solvent residual as an internal reference. Abbreviations: singlet (s), doublet (d), triplet (t), quartet (q), heptet (hept), doublet of doublets (dd), triplet of doublets (td), doublet of triplets (dt), double doublet of doublets (ddd), double triplet of doublets (dtd), double doublet of triplets (ddt), doublet of doublet of quartets (ddq), doublet of doublet of doublet of doublets (dddd), multiplet (m), and broad (br). Flash column chromatography was carried out with a Teledyne ISCO CombiFlash Rf200 and Teledyne ISCO CombiFlash Nextgen 300+ systems, using RediSep Rf Gold columns. Thin-layer chromatography (TLC) was carried out using Merck TLC Silica gel 60 F_254_ precoated plates.

### Preparation of Starting Material

The 2-tosyl-1,2-oxazetidine (**1**) was prepared according to the literature procedure,^6a^ and the recorded spectra were in agreement with the previously reported data.

### Preparation of Formyl Esters

Ethyl 2-methyl-3-oxopropanoate (**2b**),^16^*tert*-butyl 2-methyl-3-oxopropanoate (**2c**),^16^ ethyl 2-formylbutanoate (**2e**),^17^ methyl 5-chloro-2-formylpentanoate (**2h**),^18^ and ethyl 2-cyanopropanoate (**2o**)^19^ were prepared according to the literature procedures.

### Methyl 2-methyl-3-oxopropanoate (2a)

The material was prepared according to a modified literature procedure.^20^ A solution of diisopropylamine (14.3 mL, 102 mmol, 1.2 equiv) in anhydrous THF (40 mL) was cooled to −78 °C, *n*-BuLi (40.8 mL, 2.5 M in hexane, 102 mmol, 1.2 equiv) was added dropwise under N_2_, and the solution was stirred for 15 min. After that the solution of methyl propionate (7.49 g, 85 mmol, 1.0 equiv) in 20 mL THF was added. After 1 h, methyl formate (7.66 g, 128 mmol, 1.5 equiv) was added in 15 mL THF. After stirring at −78 °C for an additional 1 h, the reaction was allowed to warm up to rt and 100 mL water was added. The solution was extracted 2× with Et_2_O, the aqueous phase was acidified with 1 M HCl to pH = 3–4, and it was extracted 3× with DCM. The organic phase was dried over Na_2_SO_4_, filtered, and concentrated. The residue was distilled in a vacuum to give a tautomeric mixture. Yield: 3.50 g (35%), colorless oil. ^1^H NMR (300 MHz, CDCl_3_) δ 11.23 (d, *J* = 12.5 Hz, 0.85H), 9.77 (s, 0.15H), 6.98 (d, *J* = 12.5 Hz, 0.85H), 3.78 (s, 3H), 1.67 (s, 2.55H), 1.35 (d, *J* = 7.2 Hz, 0.45H). ^13^C{^1^H} NMR (75 MHz, CDCl_3_) δ 197.3, 173.0, 170.4, 160.2, 100.1, 52.63, 52.57, 51.6, 12.5, 10.4. HRMS (ESI) *m*/*z* [M + H]^+^ calcd for C_5_H_9_O_3_: 117.0546, found: 117.0549.

### Prop-2-yn-1-yl 2-methyl-3-oxopropanoate (2d)

The solution of *tert*-butyl 2-methyl-3-oxopropanoate (1.00 g, 6.32 mmol, 1.0 equiv) and propargyl alcohol (531 mg, 9.48 mmol, 1.5 equiv) in 30 mL toluene was heated to reflux in oil bath. After 1.5 h the reaction was evaporated. The residue was distilled in a vacuum. Yield: 444 mg (46%), colorless liquid. ^1^H NMR (500 MHz, DMSO-*d*_6_) δ 10.59 (s, 1H), 7.63 (d, *J* = 1.6 Hz, 1H), 4.68 (d, *J* = 2.5 Hz, 2H), 3.43 (t, *J* = 2.5 Hz, 1H), 1.63 (d, *J* = 1.3 Hz, 3H). ^13^C{^1^H} NMR (126 MHz, DMSO-*d*_6_) δ 167.5, 155.2, 102.2, 79.2, 76.9, 50.8, 8.6. HRMS (ESI) *m*/*z* [M + H]^+^ calcd for C_7_H_9_O_3_: 141.0546, found 141.0546.

### Ethyl 2-formylpent-4-enoate (2f)

The material was prepared according to a modified literature procedure.^21^ The mixture of ethyl 3-(allyloxy)acrylate (4.47 g, 28.6 mmol, 1.0 equiv) and 2-naphthol (144 mg, 1.0 mmol, 3.5 mol %) was heated to 145–155 °C in a bomb for 1 h in oil bath. After that the reaction was cooled to rt, 15 mL 10% NaOH solution was added, and the mixture was extracted with Et_2_O. The aqueous phase was acidified by HCl solution and extracted with Et_2_O. The organic phase was extracted by brine, dried over Na_2_SO_4_, filtered, and concentrated. The crude product was purified by gradient flash column chromatography with hexane/EtOAc eluent to give a tautomeric mixture. Yield: 1.69 g (38%), pale yellow oil. ^1^H NMR (500 MHz, DMSO-*d*_6_) δ 10.47 (s, 1H), 7.63 (s, 1H), 5.75 (ddt, *J* = 17.3, 10.1, 6.1 Hz, 1H), 4.93 (dd, *J* = 17.2, 1.9 Hz, 1H), 4.87 (dd, *J* = 10.0, 1.9 Hz, 1H), 4.05 (q, *J* = 7.1 Hz, 2H), 2.89 (dt, *J* = 6.3, 1.6 Hz, 2H), 1.17 (t, *J* = 7.1 Hz, 3H). ^13^C{^1^H} NMR (126 MHz, DMSO-*d*_6_) δ 167.7, 154.7, 136.3, 114.1, 105.7, 58.9, 27.2, 14.3. HRMS (ESI) *m*/*z* [M + H]^+^ calcd for C_8_H_13_O_3_: 157.0859, found 157.0859.

### Ethyl 2-formyl-3-methylbutanoate (2g)

A solution of diisopropylamine (16.8 mL, 120 mmol, 1.2 equiv) in anhydrous THF (60 mL) was cooled to −78 °C, *n*-BuLi (48 mL, 2.5 M in hexane, 120 mmol, 1.2 equiv) was added dropwise under N_2_, and the solution was stirred for 15 min. After that the solution of ethyl 3-methylbutanoate (13.2 g, 100 mmol, 1.0 equiv) in 30 mL THF was added and the reaction mixture was stirred for 1 h. Then, ethyl formate (11.3 g, 150 mmol, 1.5 equiv) was added in 20 mL THF. After stirring at −78 °C for an additional 1 h, the reaction was allowed to warm up to rt and 100 mL water was added. The solution was extracted 2× with Et_2_O, the aqueous phase was acidified with 1 M HCl to pH = 3–4, and it was extracted 3× with DCM. The extract was washed with brine, dried over Na_2_SO_4_, filtered, and concentrated. The residue was distilled in a vacuum (70–74 °C at 15 mbar). Yield: 7.0 g (44%), colorless liquid. ^1^H NMR (500 MHz, CDCl_3_) δ 11.57 (d, *J* = 12.4 Hz, 1H × 5.0/10), 9.69 (d, *J* = 3.9 Hz, 1H × 5.0/10), 7.01 (dd, *J* = 12.4, 0.9 Hz, 1H × 5.0/10), 4.26–4.17 (m, 4H), 2.94 (dd, *J* = 7.9, 3.8 Hz, 1H × 5.0/10), 2.58 (dtd, *J* = 13.8, 6.9, 0.9 Hz, 1H × 5.0/10), 2.45–2.35 (m, 1H × 5.0/10), 1.30 (t, *J* = 7.1 Hz, 3H), 1.27 (t, *J* = 7.1 Hz, 3H), 1.06 (d, *J* = 6.9 Hz, 6H), 1.01 (dd, *J* = 6.9, 2.7 Hz, 6H). ^13^C{^1^H} NMR (126 MHz, CDCl_3_) δ 198.5, 172.6, 169.3, 159.8, 111.0, 65.3, 61.3, 60.4, 28.6, 26.6, 22.4, 20.3, 20.2, 14.3. HRMS (ESI) *m*/*z* [M + H]^+^ calcd for C_8_H_15_O_3_: 159.1016, found 159.1015.

### Ethyl 2-formyl-4-methylpentanoate (2i)

A solution of diisopropylamine (3.9 mL, 27.6 mmol, 1.2 equiv) in anhydrous THF (20 mL) was cooled to −78 °C, *n*-BuLi (11 mL, 2.5 M in hexane, 27.6 mmol, 1.2 equiv) was added dropwise under N_2_, and the solution was stirred for 15 min. After that the solution of ethyl 4-methylpentanoate (3.32 g, 23.0 mmol, 1.0 equiv) in 10 mL THF was added and the reaction mixture was stirred for 1 h. Then, ethyl formate (5.11 g, 69.0 mmol, 3 equiv) was added dropwise. After another 1 h, the reaction was allowed to warm up to rt and 100 mL water was added. The solution was acidified with 1 M HCl to pH = 3–4, and it was extracted 3× with DCM. The organic phase was dried over Na_2_SO_4_, filtered, and concentrated. The crude product was purified by gradient column chromatography with hexane/EtOAc eluent. Yield: 2.1 g (52%), colorless oil. ^1^H NMR (500 MHz, DMSO-*d*_6_) δ 10.25 (s, 1H), 7.64 (s, 1H), 4.04 (q, *J* = 7.1 Hz, 2H), 2.04 (d, *J* = 7.2 Hz, 2H), 1.70 (hept, *J* = 6.9 Hz, 1H), 1.17 (t, *J* = 7.0 Hz, 3H), 0.81 (d, *J* = 6.7 Hz, 6H). ^13^C{^1^H} NMR (126 MHz, DMSO-*d*_6_) δ 168.3, 154.7, 107.1, 58.7, 32.0, 27.3, 22.2, 14.3. HRMS (ESI) *m*/*z* [M + H]^+^ calcd for C_9_H_17_O_3_: 173.1172, found 173.1173.

### Ethyl 2-(3,4-dimethoxybenzyl)-3-oxopropanoate (2j)

Ethyl 3-(3,4-dimethoxyphenyl)propanoate (2.38 g, 10.0 mmol, 1.0 equiv) and ethyl formate (2.42 mL, 30.0 mmol, 3.0 equiv) was dissolved in anhydrous THF (30 mL) and NaH (1.2 g, 60% in mineral oil, 30 mmol, 3.0 equiv) was added under N_2_. The suspension was stirred for 19 h, and water was added. The solution was acidified with 1 M HCl to pH = 3–4 and extracted with DCM. The extract was dried over Na_2_SO_4_, filtered, and concentrated. The crude product was purified by gradient column chromatography with hexane/EtOAc eluent. Yield: 392 mg (15%), yellow oil. ^1^H NMR (500 MHz, DMSO-*d*_6_) δ 10.62 (s, 1H), 7.71 (s, 1H), 6.81–6.78 (m, 2H), 6.69–6.66 (m, 1H), 4.04 (q, *J* = 7.1 Hz, 2H), 3.69 (d, *J* = 3.9 Hz, 6H), 3.42 (s, 2H), 1.16 (t, *J* = 7.1 Hz, 3H). ^13^C{^1^H} NMR (126 MHz, DMSO-*d*_6_) δ 167.9, 154.7, 148.4, 146.9, 133.7, 119.9, 112.4, 111.9, 107.8, 58.9, 55.5, 55.3, 28.3, 14.3. HRMS (ESI) *m*/*z* [M + H]^+^ calcd for C_14_H_19_O_5_: 267.1227, found 267.1224.

### Methyl 3-(benzo[*d*][1,3]dioxol-5-yl)-2-formylpropanoate (2k)

A solution of diisopropylamine (1.0 mL, 7.2 mmol, 1.2 equiv) in anhydrous THF (6 mL) was cooled to −78 °C, *n*-BuLi (2.88 mL, 2.5 M in hexane, 7.2 mmol, 1.2 equiv) was added dropwise under N_2_, and the solution was stirred for 15 min. After that the solution of methyl 3-(benzo[*d*][1,3]dioxol-5-yl)propanoate (1.25 g, 6.0 mmol, 1.0 equiv) in 6 mL THF was added. After 5 min, ethyl formate (2.22 g, 30.0 mmol, 5.0 equiv) was added dropwise. After 1 h, the reaction was poured into 1 M HCl, and the solution was extracted 3× with EtOAc. The organic phase was dried over Na_2_SO_4_, filtered, and evaporated. The residue was purified by gradient column chromatography with hexane/EtOAc eluent to give a tautomeric mixture. Yield: 697 mg (49%), white solid, mp 98 °C. ^1^H NMR (500 MHz, CDCl_3_) δ 11.43 (d, *J* = 12.6 Hz, 0.66H), 9.71 (d, *J* = 1.9 Hz, 0.34H), 7.02 (d, *J* = 12.3 Hz, 0.66H), 6.72–6.69 (m, 1H), 6.68–6.65 (m, 1H), 6.64–6.60 (m, 1H), 5.91 (s, 0.68H), 5.90 (s, 1.32H), 3.73 (s, 1.02H), 3.73 (s, 1.98H), 3.61–3.55 (m, 0.34H), 3.31 (s, 1.32H), 3.17–3.06 (m, 0.68H). ^13^C{^1^H} NMR (126 MHz, CDCl_3_) δ 196.3, 172.5, 169.1, 161.9, 147.9, 147.7, 146.6, 146.0, 133.9, 131.1, 122.0, 121.3, 109.3, 108.9, 108.4, 108.2, 104.7, 101.1, 100.9, 60.5, 52.5, 51.6, 32.9, 32.1. HRMS (ESI) *m*/*z* [M + Na]^+^ calcd for C_12_H_12_NaO_5_: 259.0577, found 259.0574.

### Dimethyl 2-formylsuccinate (2l)

The material was prepared according to the modification of literature procedure.^22^ The suspension of NaH (2.30 g, 60% in mineral oil, 57.5 mmol, 1.51 equiv) in anhydrous THF (50 mL) was cooled to 0 °C, and methanol (0.155 mL, 3.82 mmol, 0.1 equiv), dimethyl succinate (5.0 mL, 38.2 mmol, 1.00 equiv), and methyl formate (15 mL, 245 mmol, 6.41 equiv) were added under N_2_. The mixture was warmed to rt and stirred overnight. After that the mixture was concentrated in a vacuum, water was added to the concentrate and the solution was acidified with 1 M HCl to pH = 2–3. The mixture was extracted with DCM, the organic phase was washed with brine, dried over Na_2_SO_4_, filtered and evaporated. The resulting residue was purified by gradient column chromatography with DCM/methanol eluent. Yield: 2.84 g (43%), pale yellow oil. ^1^H NMR (500 MHz, DMSO-*d*_6_) δ 10.89 (s, 1H), 7.70 (s, 1H), 3.59 (s, 3H), 3.56 (s, 3H), 3.16 (s, 2H). ^13^C{^1^H} NMR (126 MHz, DMSO-*d*_6_) δ 171.3, 167.9, 156.3, 101.7, 51.4, 50.8, 28.6. HRMS (ESI) *m*/*z* [M + H]^+^ calcd for C_7_H_11_O_5_: 175.0601, found 175.0601.

### Preparation of Morpholines 3a–l. General Procedure

A mixture of 2-tosyl-1,2-oxazetidine (**1)** (53.3 mg, 0.25 mmol, 1.0 equiv), formyl ester (**2a**–**l**) (0.25 mmol, 1.0 equiv), K_2_CO_3_ (0.30 mmol, 1.2 equiv), and 1,4-dioxane (500 μL, 0.5 M) was stirred overnight at rt. Then, the reaction mixture was filtered, concentrated, and the resulting residue was purified by gradient column chromatography using hexane/EtOAc as eluent.

### Methyl 3-hydroxy-2-methyl-4-tosylmorpholine-2-carboxylate (3a)

Prepared according to the general procedure from 2-tosyl-1,2-oxazetidine (**1**, 53.3 mg, 0.25 mmol), methyl 2-methyl-3-oxopropanoate (**2a**, 29.0 mg, 0.25 mmol), and K_2_CO_3_ (41.5 mg, 0.30 mmol). Yield: 64.8 mg (79%), dr: 2.0/1, white solid, mp 150 °C (major) and 135 °C (minor).

The reaction was also performed in gram scale from 2-tosyl-1,2-oxazetidine (**1**, 2.50 g, 11.7 mmol), methyl 2-methyl-3-oxopropanoate (**2a**, 1.39 g, 11.7 mmol), and K_2_CO_3_ (1.94 g, 14.0 mmol). Yield: 2.79 g (72%), dr: 3.4/1. ^1^H NMR (500 MHz, CDCl_3_, major, **3a′**) δ 7.71 (d, *J* = 8.3 Hz, 2H), 7.30 (d, *J* = 8.0 Hz, 2H), 5.64 (d, *J* = 7.6 Hz, 1H), 4.05–3.97 (m, 1H), 3.84–3.79 (m, 1H), 3.68 (s, 3H), 3.31–3.19 (m, 2H), 2.82 (d, *J* = 7.7 Hz, 1H), 2.42 (s, 3H), 1.38 (s, 3H). ^13^C{^1^H} NMR (126 MHz, CDCl_3_, major, **3a′**) δ 171.5, 144.1, 135.8, 129.7, 127.9, 79.9, 77.5, 62.8, 52.5, 37.9, 22.8, 21.6. ^1^H NMR (500 MHz, CDCl_3_, minor, **3a″**) δ 7.73 (d, *J* = 8.3 Hz, 2H), 7.31 (d, *J* = 8.1 Hz, 2H), 5.41 (d, *J* = 7.5 Hz, 1H), 3.98 (td, *J* = 12.0, 3.5 Hz, 1H), 3.90 (dd, *J* = 12.0, 4.2 Hz, 1H), 3.79 (s, 3H), 3.39 (dd, *J* = 12.1, 3.0 Hz, 1H), 3.16 (td, *J* = 12.1, 4.2 Hz, 1H), 2.92 (d, *J* = 8.0 Hz, 1H), 2.42 (s, 3H), 1.58 (s, 3H). ^13^C{^1^H} NMR (126 MHz, CDCl_3_, minor, **3a″**) δ 171.7, 144.3, 135.7, 129.9, 127.9, 79.6, 78.9, 60.4, 53.0, 38.7, 21.7, 18.0. HRMS (ESI) *m*/*z* [M + Na]^+^ calcd for C_14_H_19_NNaO_6_S: 352.0825, found 352.0820.

### Ethyl 3-hydroxy-2-methyl-4-tosylmorpholine-2-carboxylate (3b)

Prepared according to the general procedure from 2-tosyl-1,2-oxazetidine (**1**, 64.0 mg, 0.30 mmol), ethyl 2-methyl-3-oxopropanoate (**2b**, 39.0 mg, 0.30 mmol), and K_2_CO_3_ (49.8 mg, 0.36 mmol). Yield: 88.6 mg (86%), white solid, mp 111 °C. ^1^H NMR (500 MHz, CDCl_3_) δ 7.72 (d, *J* = 8.3 Hz, 2H), 7.30 (d, *J* = 8.1 Hz, 2H), 5.67 (s, 1H), 4.25–4.13 (m, 2H), 4.06 (td, *J* = 12.2, 3.7 Hz, 1H), 3.81 (dd, *J* = 12.2, 3.6 Hz, 1H), 3.28 (dd, *J* = 12.0, 3.6 Hz, 1H), 3.19 (td, *J* = 12.2, 3.9 Hz, 1H), 2.42 (s, 3H), 1.38 (s, 3H), 1.27 (t, *J* = 7.1 Hz, 3H). ^13^C{^1^H} NMR (126 MHz, CDCl_3_) δ 170.9, 144.2, 135.9, 129.8, 128.0, 79.7, 77.7, 62.8, 61.8, 38.0, 22.8, 21.7, 14.2. HRMS (ESI) *m*/*z* [M + Na]^+^ calcd for C_15_H_21_NNaO_6_S: 366.0982, found 366.0978.

### *tert*-Butyl 3-hydroxy-2-methyl-4-tosylmorpholine-2-carboxylate (3c)

Prepared according to the general procedure from 2-tosyl-1,2-oxazetidine (**1**, 64.0 mg, 0.30 mmol), *tert*-butyl 2-methyl-3-oxopropanoate (**2c**, 47.5 mg, 0.30 mmol), and K_2_CO_3_ (49.8 mg, 0.36 mmol). Yield: 88.9 mg (81%), white solid, mp 174 °C. ^1^H NMR (500 MHz, CDCl_3_) δ 7.74 (d, *J* = 8.3 Hz, 2H), 7.30 (d, *J* = 8.1 Hz, 2H), 5.66 (d, *J* = 8.7 Hz, 1H), 4.11 (td, *J* = 12.3, 3.6 Hz, 1H), 3.76 (dd, *J* = 12.0, 3.8 Hz, 1H), 3.26 (dd, *J* = 12.0, 3.5 Hz, 1H), 3.09 (td, *J* = 12.3, 4.0 Hz, 1H), 2.68 (d, *J* = 8.7 Hz, 1H), 2.42 (s, 3H), 1.50 (s, 9H), 1.34 (s, 3H). ^13^C{^1^H} NMR (126 MHz, CDCl_3_) δ 169.8, 144.1, 135.9, 129.8, 128.1, 83.1, 79.7, 78.0, 62.6, 38.0, 28.0, 22.7, 21.7. HRMS (ESI) *m*/*z* [M + Na]^+^ calcd for C_17_H_25_NNaO_6_S: 394.1295, found 394.1290.

### Prop-2-yn-1-yl 3-hydroxy-2-methyl-4-tosylmorpholine-2-carboxylate (3d)

Prepared according to the general procedure from 2-tosyl-1,2-oxazetidine (**1**, 64.0 mg, 0.30 mmol), prop-2-yn-1-yl 2-methyl-3-oxopropanoate (**2d**, 42.0 mg, 0.30 mmol), and K_2_CO_3_ (49.8 mg, 0.36 mmol). Yield: 41.2 mg (39%) dr: 3.0 (only the major diastereomer could be isolated), pale yellow solid, mp 131 °C. ^1^H NMR (500 MHz, CDCl_3_,) δ 7.72 (d, *J* = 8.3 Hz, 2H), 7.30 (d, *J* = 8.0 Hz, 2H), 5.66 (d, *J* = 5.7 Hz, 1H), 4.79 (dd, *J* = 15.5, 2.5 Hz, 1H), 4.55 (dd, *J* = 15.4, 2.5 Hz, 1H), 3.99 (ddd, *J* = 12.0, 9.9, 6.0 Hz, 1H), 3.85–3.80 (m, 1H), 3.29–3.24 (m, 2H), 2.94 (d, *J* = 7.3 Hz, 1H), 2.49 (t, *J* = 2.5 Hz, 1H), 2.42 (s, 3H), 1.40 (s, 3H). ^13^C{^1^H} NMR (126 MHz, CDCl_3_) δ 170.4, 144.2, 135.8, 129.8, 128.0, 79.8, 77.4, 76.9, 75.7, 63.0, 52.9, 37.9, 22.7, 21.7. HRMS (ESI) *m*/*z* [M + Na]^+^ calcd for C_16_H_19_NNaO_6_S: 376.0825, found 376.0819.

### Ethyl 2-ethyl-3-hydroxy-4-tosylmorpholine-2-carboxylate (3e)

Prepared according to the general procedure from 2-tosyl-1,2-oxazetidine (**1**, 64.0 mg, 0.30 mmol), ethyl 2-formylbutanoate (**2e**, 43.3 mg, 0.30 mmol), and K_2_CO_3_ (49.8 mg, 0.36 mmol). Yield: 88.4 mg (82%), dr: 2.3, pale yellow solid, mp 125 °C (major). ^1^H NMR (500 MHz, CDCl_3_, major, **3e′**) δ 7.73 (d, *J* = 8.3 Hz, 2H), 7.31 (d, *J* = 8.0 Hz, 2H), 5.67 (d, *J* = 7.9 Hz, 1H), 4.26–4.13 (m, 2H), 4.09 (td, *J* = 12.2, 3.7 Hz, 1H), 3.82 (dd, *J* = 12.0, 3.9 Hz, 1H), 3.29 (dd, *J* = 12.1, 3.6 Hz, 1H), 3.19 (td, *J* = 12.2, 4.0 Hz, 1H), 2.63 (d, *J* = 8.1 Hz, 1H), 2.43 (s, 3H), 1.81–1.67 (m, 2H), 1.28 (t, *J* = 7.1 Hz, 3H), 0.88 (t, *J* = 7.6 Hz, 3H). ^13^C{^1^H} NMR (126 MHz, CDCl_3_, major, **3e′**) δ 169.7, 144.2, 136.0, 129.8, 128.0, 82.9, 77.5, 62.8, 61.6, 38.3, 29.3, 21.7, 14.3, 7.5. ^1^H NMR (500 MHz, CDCl_3_, minor, **3e″**) δ 7.73 (d, *J* = 8.1 Hz, 2H), 7.29 (d, *J* = 8.0 Hz, 2H), 5.40 (s, 1H), 4.30–4.18 (m, 2H), 3.90–3.81 (m, 2H), 3.39–3.33 (m, 1H), 3.12 (td, *J* = 11.6, 5.2 Hz, 1H), 2.41 (s, 3H), 2.28 (dq, *J* = 15.0, 7.5 Hz, 1H), 1.83 (dq, *J* = 15.0, 7.5 Hz, 1H), 1.29 (t, *J* = 7.1 Hz, 3H), 0.83 (t, *J* = 7.5 Hz, 3H). ^13^C{^1^H} NMR (126 MHz, CDCl_3_, minor, **3e″**) δ 170.7, 144.5, 136.1, 130.2, 128.2, 83.3, 79.1, 62.3, 60.3, 38.8, 23.2, 22.0, 14.6, 7.1. HRMS (ESI) *m*/*z* [M + Na]^+^ calcd for C_16_H_23_NNaO_6_S: 380.1138, found 380.1132.

### Ethyl 2-allyl-3-hydroxy-4-tosylmorpholine-2-carboxylate (3f)

Prepared according to the general procedure from 2-tosyl-1,2-oxazetidine (**1**, 64.0 mg, 0.30 mmol), ethyl 2-formylpent-4-enoate (**2f**, 46.9 mg, 0.30 mmol), and K_2_CO_3_ (49.8 mg, 0.360 mmol). Yield: 57.0 mg (51%) dr: 2.1 (only the major diastereomer could be isolated), pale yellow oil. ^1^H NMR (500 MHz, CDCl_3_) δ 7.71 (d, *J* = 8.3 Hz, 2H), 7.29 (d, *J* = 8.1 Hz, 2H), 5.77–5.69 (m, 1H), 5.67 (d, *J* = 7.3 Hz, 1H), 5.10–5.04 (m, 2H), 4.22–4.02 (m, 3H), 3.84–3.79 (m, 1H), 3.24 (dd, *J* = 9.3, 2.9 Hz, 2H), 3.02 (d, *J* = 7.5 Hz, 1H), 2.50 (dd, *J* = 14.2, 6.6 Hz, 1H), 2.44–2.39 (m, 4H), 1.23 (t, *J* = 7.1 Hz, 3H). ^13^C{^1^H} NMR (126 MHz, CDCl_3_) δ 169.4, 144.1, 135.9, 131.0, 129.7, 127.9, 119.4, 82.4, 77.2, 62.8, 61.6, 40.8, 38.2, 21.6, 14.3. HRMS (ESI) *m*/*z* [M–H_2_O+H]^+^ calcd for C_17_H_22_NO_5_S: 352.1213, found 352.1209.

### Ethyl 3-hydroxy-2-isopropyl-4-tosylmorpholine-2-carboxylate (3g)

Prepared according to the general procedure from 2-tosyl-1,2-oxazetidine (**1**, 64.0 mg, 0.30 mmol), ethyl 2-formyl-3-methylbutanoate (**2g**, 47.5 mg, 0.30 mmol), and K_2_CO_3_ (49.8 mg, 0.36 mmol). Yield: 47.0 mg (42%), dr: 6.9 (only the major diastereomer could be isolated), pale yellow oil. ^1^H NMR (500 MHz, CDCl_3_) δ 7.71 (d, *J* = 8.3 Hz, 2H), 7.29 (d, *J* = 8.1 Hz, 2H), 5.80 (d, *J* = 5.4 Hz, 1H), 4.20 (ddq, *J* = 37.6, 10.7, 7.1 Hz, 2H), 4.02 (td, *J* = 12.2, 3.7 Hz, 1H), 3.79 (dd, *J* = 12.0, 3.8 Hz, 1H), 3.23 (dd, *J* = 12.0, 3.6 Hz, 1H), 3.14 (td, *J* = 12.2, 4.0 Hz, 1H), 2.84 (d, *J* = 7.3 Hz, 1H), 2.41 (s, 3H), 1.99 (hept, *J* = 7.0 Hz, 1H), 1.28 (t, *J* = 7.1 Hz, 3H), 0.99 (d, *J* = 7.0 Hz, 3H), 0.90 (d, *J* = 6.9 Hz, 3H). ^13^C{^1^H} NMR (126 MHz, CDCl_3_) δ 168.2, 144.0, 135.9, 129.7, 128.0, 85.2, 76.9, 62.6, 61.3, 38.2, 33.9, 21.7, 16.9, 16.7, 14.4. HRMS (ESI) *m*/*z* [M + Na]^+^ calcd for C_17_H_25_NNaO_6_S: 394.1295, found 394.1293.

### Methyl 2-(3-chloropropyl)-3-hydroxy-4-tosylmorpholine-2-carboxylate (3h)

Prepared according to the general procedure from 2-tosyl-1,2-oxazetidine (**1**, 53.3 mg, 0.25 mmol), methyl 5-chloro-2-formylpentanoate (**2h**, 44.7 mg, 0.25 mmol), and K_2_CO_3_ (41.5 mg, 0.30 mmol). Yield: 30.5 mg (31%), dr: 1.5 (only the major diastereomer could be isolated), pale yellow oil. ^1^H NMR (500 MHz, CDCl_3_,) δ 7.71 (d, *J* = 8.3 Hz, 2H), 7.31 (d, *J* = 8.1 Hz, 2H), 5.62 (s, 1H), 4.07 (td, *J* = 11.6, 4.9 Hz, 1H), 3.82 (dd, *J* = 12.1, 2.3 Hz, 2H), 3.66 (s, 3H), 3.56–3.41 (m, 2H), 3.32–3.22 (m, 2H), 2.43 (s, 3H), 1.94–1.78 (m, 3H), 1.65–1.55 (m, 1H). ^13^C{^1^H} NMR (126 MHz, CDCl_3_,) δ 170.2, 144.3, 135.9, 129.8, 127.9, 82.2, 77.5, 62.8, 52.4, 44.8, 38.1, 33.4, 26.5, 21.7. HRMS (ESI) *m*/*z* [M + Na]^+^ calcd for C_16_H_22_ClNNaO_6_S: 414.0749, found 414.0740.

### Ethyl 3-hydroxy-2-isobutyl-4-tosylmorpholine-2-carboxylate (3i)

Prepared according to the general procedure from 2-tosyl-1,2-oxazetidine (**1**, 64.0 mg, 0.30 mmol), ethyl 2-formyl-4-methylpentanoate (**2i**, 51.7 mg, 0.30 mmol), and K_2_CO_3_ (49.8 mg, 0.36 mmol). Yield: 70.9 mg (61%), dr: 1.8/1, pale yellow oil. ^1^H NMR (500 MHz, CDCl_3_, major, **3i′**) δ 7.70 (d, *J* = 8.2 Hz, 2H), 7.29 (d, *J* = 8.1 Hz, 2H), 5.59 (d, *J* = 8.0 Hz, 1H), 4.22–4.10 (m, 3H), 3.77 (dd, *J* = 12.0, 3.8 Hz, 1H), 3.26 (dd, *J* = 12.0, 3.6 Hz, 1H), 3.17 (td, *J* = 12.2, 4.0 Hz, 1H), 2.79 (d, *J* = 8.1 Hz, 1H), 2.41 (s, 3H), 1.70–1.55 (m, 3H), 1.26 (t, *J* = 7.1 Hz, 3H), 0.91 (d, *J* = 6.3 Hz, 3H), 0.84 (d, *J* = 6.3 Hz, 3H). ^13^C{^1^H} NMR (126 MHz, CDCl_3_, major, **3i′**) δ 169.9, 144.1, 135.9, 129.7, 128.0, 82.6, 78.3, 62.3, 61.5, 44.7, 38.1, 24.2, 24.1, 23.9, 21.7, 14.2. ^1^H NMR (500 MHz, CDCl_3_, minor, **3i″**) δ 7.72 (d, *J* = 8.2 Hz, 2H), 7.28 (d, *J* = 8.0 Hz, 2H), 5.31 (d, *J* = 8.2 Hz, 1H), 4.29–4.14 (m, 2H), 3.93–3.83 (m, 2H), 3.40–3.35 (m, 1H), 3.12–3.05 (m, 2H), 2.40 (s, 3H), 2.28–2.19 (m, 1H), 1.74–1.63 (m, 2H), 1.29 (t, *J* = 7.1 Hz, 3H), 0.95 (d, *J* = 6.4 Hz, 3H), 0.84 (d, *J* = 6.2 Hz, 3H). ^13^C{^1^H} NMR (126 MHz, CDCl_3_, minor, **3i″**) δ 171.0, 144.2, 135.7, 129.8, 127.9, 82.2, 79.3, 61.9, 60.3, 38.5, 37.7, 23.7, 23.7, 22.8, 21.7, 14.2. HRMS (ESI) *m*/*z* [M + Na]^+^ calcd for C_18_H_27_NNaO_6_S: 408.1451, found 408.1447.

### Ethyl 2-(3,4-dimethoxybenzyl)-3-hydroxy-4-tosylmorpholine-2-carboxylate (3j)

Prepared according to the general procedure from 2-tosyl-1,2-oxazetidine (**1**, 64.0 mg, 0.30 mmol), ethyl 2-(3,4-dimethoxybenzyl)-3-oxopropanoate (**2j**, 79.9 mg, 0.30 mmol), and K_2_CO_3_ (49.8 mg, 0.36 mmol). Yield: 101.6 mg (71%), dr: 2.7, pale yellow oil. ^1^H NMR (500 MHz, CDCl_3_, major, **3j′**) δ 7.71 (d, *J* = 8.1 Hz, 2H), 7.29 (d, *J* = 8.0 Hz, 2H), 6.78–6.67 (m, 3H), 5.71 (d, *J* = 7.3 Hz, 1H), 4.08–4.00 (m, 1H), 3.96 (dddd, *J* = 17.9, 10.7, 7.1, 3.6 Hz, 2H), 3.88–3.83 (m, 1H), 3.83 (s, 3H), 3.82 (s, 3H), 3.31–3.26 (m, 2H), 3.00–2.90 (m, 3H), 2.41 (s, 3H), 1.07 (t, *J* = 7.1 Hz, 3H). ^13^C{^1^H} NMR (126 MHz, CDCl_3_, **3j′**) δ 169.2, 148.6, 148.3, 144.2, 135.9, 129.8, 127.9, 127.0, 122.4, 113.7, 111.1, 83.3, 77.6, 62.9, 61.4, 56.0, 42.1, 38.1, 21.7, 14.0. ^1^H NMR (500 MHz, CDCl_3_, minor, **3j″**) δ 7.76 (d, *J* = 8.3 Hz, 2H), 7.29 (d, *J* = 8.1 Hz, 2H), 6.77–6.68 (m, 3H), 5.57–5.53 (m, 1H), 4.21 (td, *J* = 11.9, 3.6 Hz, 1H), 4.11 (qq, *J* = 7.0, 3.6 Hz, 2H), 3.99–3.94 (m, 1H), 3.84 (s, 3H), 3.83 (s, 3H), 3.50–3.40 (m, 2H), 3.19 (td, *J* = 12.1, 4.2 Hz, 1H), 3.14 (d, *J* = 14.9 Hz, 1H), 3.11–3.06 (m, 1H), 2.42 (s, 3H), 1.17 (t, *J* = 7.1 Hz, 3H). ^13^C{^1^H} NMR (126 MHz, CDCl_3_, **3j″**) δ 170.1, 148.9, 148.3, 144.3, 135.6, 129.9, 128.0, 127.0, 122.0, 113.1, 111.4, 83.2, 78.8, 61.9, 60.5, 56.0, 56.0, 38.8, 35.5, 21.7, 14.1. HRMS (ESI) *m*/*z* [M + Na]^+^ calcd for C_23_H_29_NNaO_8_S: 502.1506, found 502.1500.

### Methyl 2-(benzo[*d*][1,3]dioxol-5-ylmethyl)-3-hydroxy-4-tosylmorpholine-2-carboxylate (3k)

Prepared according to the general procedure from 2-tosyl-1,2-oxazetidine (**1**, 64.0 mg, 0.30 mmol), ethyl 3-(benzo[*d*][1,3]dioxol-5-yl)-2-formylpropanoate (70.9 mg, 0.30 mmol), and K_2_CO_3_ (49.8 mg, 0.36 mmol). Yield: 59.3 mg (44%), dr: 3.7 (only the major diastereomer could be isolated), pale brown solid, mp 173 °C. ^1^H NMR (500 MHz, CDCl_3_) δ 7.70 (d, *J* = 8.3 Hz, 2H), 7.30 (d, *J* = 8.1 Hz, 2H), 6.67 (dd, *J* = 4.9, 3.1 Hz, 2H), 6.54 (dd, *J* = 8.0, 1.7 Hz, 1H), 5.90 (d, *J* = 1.6 Hz, 2H), 5.67 (s, 1H), 3.99 (td, *J* = 12.0, 3.9 Hz, 1H), 3.88–3.82 (m, 1H), 3.49 (s, 3H), 3.33 (td, *J* = 12.0, 3.8 Hz, 1H), 3.27 (dd, *J* = 12.1, 3.8 Hz, 1H), 2.95 (d, *J* = 14.0 Hz, 1H), 2.89 (d, *J* = 14.0 Hz, 1H), 2.42 (s, 3H). ^13^C{^1^H} NMR (126 MHz, CDCl_3_) δ 169.7, 147.5, 146.8, 144.2, 135.9, 129.8, 128.1, 127.9, 123.1, 110.6, 108.1, 101.0, 83.6, 77.4, 63.0, 52.0, 42.2, 38.1, 21.7. HRMS (ESI) *m*/*z* [M–H_2_O+H]^+^ calcd for C_21_H_22_NO_7_S: 432.1111, found 432.1107.

### 7a-Methyl-4-tosylhexahydro-6*H*-furo[3,2-*b*][1,4]oxazin-6-one (3l)

Prepared according to the general procedure from 2-tosyl-1,2-oxazetidine (**1**, 64.0 mg, 0.30 mmol), dimethyl 2-formylsuccinate (52.3 mg, 0.30 mmol), and K_2_CO_3_ (49.8 mg, 0.36 mmol). Yield: 51.8 mg (49%), white solid, mp 134 °C. ^1^H NMR (500 MHz, CDCl_3_) δ 7.73 (d, *J* = 8.3 Hz, 2H), 7.33 (d, *J* = 8.1 Hz, 2H), 6.44 (s, 1H), 3.99 (td, *J* = 12.0, 2.7 Hz, 1H), 3.91–3.86 (m, 4H), 3.48–3.43 (m, 1H), 3.02 (d, *J* = 17.0 Hz, 1H), 2.96 (td, *J* = 12.0, 3.4 Hz, 1H), 2.71 (d, *J* = 17.0 Hz, 1H), 2.43 (s, 3H). ^13^C{^1^H} NMR (126 MHz, CDCl_3_) δ 170.2, 167.8, 145.0, 134.3, 130.0, 128.3, 84.0, 79.2, 63.2, 53.7, 42.8, 38.2, 21.7. HRMS (ESI) *m*/*z* [M + H]^+^ calcd for C_15_H_18_NO_7_S: 356.0798, found 356.0794.

### Ethyl 2-methyl-2-(2-(4-methylphenylsulfonamido)ethoxy)-3-oxobutanoate (5a)

The 2-tosyl-1,2-oxazetidine (**1**, 53.3 mg, 0.25 mmol, 1.0 equiv), ethyl 2-methyl-3-oxobutanoate (29.0 mg, 0.25 mmol, 1.0 equiv), and K_2_CO_3_ (41.5 mg, 0.30 mmol, 1.2 equiv) in 1,4-dioxane (0.5 mL) was stirred overnight at room temperature. Then, the reaction mixture was diluted with DCM and extracted with 1 M HCl. The organic phase was separated, dried over Na_2_SO_4_, filtered, and concentrated. The crude product was purified by gradient column chromatography with hexane/EtOAc eluent. Yield: 41.4 mg (28%), yellow oil. ^1^H NMR (500 MHz, CDCl_3_) δ 7.75 (d, *J* = 8.3 Hz, 2H), 7.29 (d, *J* = 8.0 Hz, 2H), 5.37 (t, *J* = 5.4 Hz, 1H), 4.21 (q, *J* = 7.1 Hz, 2H), 3.60–3.53 (m, 1H), 3.50–3.40 (m, 1H), 3.21–3.11 (m, 2H), 2.41 (s, 3H), 2.15 (s, 3H), 1.46 (s, 3H), 1.26 (t, *J* = 7.1 Hz, 3H). ^13^C{^1^H} NMR (126 MHz, CDCl_3_) δ 143.6, 137.1, 129.8, 127.3, 86.6, 64.1, 62.4, 43.3, 25.1, 21.6, 18.2, 14.2. HRMS (ESI) *m*/*z* [M + Na]^+^ calcd for C_16_H_23_NNaO_6_S: 380.1138, found 380.1134.

### *N*-(2-((3-Acetyl-2-oxotetrahydrofuran-3-yl)oxy)ethyl)-4-methylbenzenesulfonamide (5b)

The 2-tosyl-1,2-oxazetidine (**1**, 64.0 mg, 0.30 mmol, 1.0 equiv), 3-acetyldihydrofuran-2(3H)-one (38.4 mg, 0.30 mmol, 1.0 equiv), and K_2_CO_3_ (49.8 mg, 0.36 mmol, 1.2 equiv) in 1,4-dioxane (0.7 mL) was stirred overnight at room temperature. Then, the reaction mixture was diluted with DCM and extracted with 1 M HCl. The organic phase was separated, dried over Na_2_SO_4_, filtered, and concentrated. The crude product was purified by gradient column chromatography with hexane/EtOAc eluent. Yield: 54.9 mg (55%), yellow oil. ^1^H NMR (500 MHz, CDCl_3_) δ 7.76 (d, *J* = 8.3 Hz, 2H), 7.31 (d, *J* = 8.0 Hz, 2H), 5.11 (t, *J* = 5.7 Hz, 1H), 4.38 (td, *J* = 8.9, 3.1 Hz, 1H), 4.31 (td, *J* = 8.8, 7.1 Hz, 1H), 3.65–3.59 (m, 1H), 3.54–3.47 (m, 1H), 3.27–3.16 (m, 2H), 2.62 (ddd, *J* = 13.3, 7.0, 3.1 Hz, 1H), 2.43 (s, 3H), 2.37–2.31 (m, 1H), 2.29 (s, 3H). ^13^C{^1^H} NMR (126 MHz, CDCl_3_) δ 203.8, 171.6, 143.7, 137.0, 129.9, 127.1, 86.9, 65.9, 65.2, 43.0, 29.7, 25.3, 21.6. HRMS (ESI) *m*/*z* [M + NH_4_]^+^ calcd for C_15_H_23_N_2_O_6_S: 359.1271, found 359.1263.

### Methyl 1-(2-((4-methylphenyl)sulfonamido)ethoxy)-2-oxocyclohexane-1-carboxylate (5c)

The 2-tosyl-1,2-oxazetidine (**1**, 53.3 mg, 0.25 mmol, 1.0 equiv), methyl 2-oxocyclohexane-1-carboxylate (39.1 mg, 0.25 mmol, 1.0 equiv), and K_2_CO_3_ (41.5 mg, 0.30 mmol, 1.2 equiv) in 1,4-dioxane (0.5 mL) was stirred 3 days at room temperature. Then, the reaction mixture was diluted with DCM and extracted with 1 M HCl. The organic phase was separated, dried over Na_2_SO_4_, filtered, and concentrated. The resulting residue was purified by gradient column chromatography with hexane/EtOAc eluent. Yield: 49.9 mg (54%), yellow oil. ^1^H NMR (500 MHz, CDCl_3_) δ 7.73 (d, *J* = 8.3 Hz, 2H), 7.27 (d, *J* = 8.1 Hz, 2H), 5.58 (t, *J* = 5.2 Hz, 1H), 3.77 (s, 3H), 3.58 (ddd, *J* = 9.5, 5.7, 3.8 Hz, 1H), 3.45 (ddd, *J* = 9.7, 6.4, 3.7 Hz, 1H), 3.19–3.11 (m, 2H), 2.53–2.41 (m, 2H), 2.40 (s, 3H), 2.27–2.20 (m, 1H), 1.96–1.89 (m, 1H), 1.87–1.67 (m, 3H), 1.63–1.55 (m, 1H). ^13^C{^1^H} NMR (126 MHz, CDCl_3_) δ 205.7, 170.3, 143.3, 137.0, 129.7, 127.3, 86.5, 64.5, 52.8, 43.3, 39.9, 35.6, 26.9, 21.6, 20.8. HRMS (ESI) *m*/*z* [M–H_2_O+H]^+^ calcd for C_17_H_22_NO_5_S: 352.1213, found 352.1210.

### Ethyl 4,4-dimethyl-2-(2-((4-methylphenyl)sulfonamido)ethoxy)-3-oxopentanoate (5d)

The 2-tosyl-1,2-oxazetidine (**1**, 53.3 mg, 0.25 mmol, 1.0 equiv), ethyl 4,4-dimethyl-3-oxopentanoate (43.1 mg, 0.25 mmol, 1.0 equiv), and K_2_CO_3_ (41.5 mg, 0.30 mmol, 1.2 equiv) in 1,4-dioxane (0.5 mL) was stirred overnight at room temperature. Then, the reaction mixture was diluted with DCM and extracted with 1 M HCl. The organic phase was separated, dried over Na_2_SO_4_, filtered, and concentrated. The crude product was purified by gradient column chromatography with hexane/EtOAc eluent. Yield: 63.8 mg (66%), yellow oil. ^1^H NMR (500 MHz, CDCl_3_) δ 7.73 (d, *J* = 8.3 Hz, 2H), 7.27 (d, *J* = 8.0 Hz, 2H), 5.44 (t, *J* = 5.8 Hz, 1H), 4.70 (s, 1H), 4.29–4.13 (m, 2H), 3.66 (ddd, *J* = 9.6, 5.7, 4.0 Hz, 1H), 3.57 (ddd, *J* = 9.8, 6.3, 3.9 Hz, 1H), 3.14 (dq, *J* = 9.8, 5.0, 4.5 Hz, 2H), 2.40 (s, 3H), 1.25 (t, *J* = 7.1 Hz, 3H), 1.16 (s, 9H). ^13^C{^1^H} NMR (126 MHz, CDCl_3_) δ 206.5, 168.1, 143.4, 137.0, 129.8, 127.2, 79.9, 70.1, 62.1, 44.5, 43.1, 26.2, 21.6, 14.1. HRMS (ESI) *m*/*z* [M + H]^+^ calcd for C_18_H_28_NO_6_S: 386.1632, found 386.1627.

### *tert*-Butyl 2,2-bis(2-(4-methylphenylsulfonamido)ethoxy)-3-oxobutanoate (5e)

The 2-tosyl-1,2-oxazetidine (**1**, 53.3 mg, 0.25 mmol, 1.0 equiv), *tert*-butyl 3-oxobutanoate (39.6 mg, 0.25 mmol, 1.0 equiv), and K_2_CO_3_ (41.5 mg, 0.30 mmol, 1.2 equiv) in 1,4-dioxane (0.5 mL) was stirred overnight at room temperature. Then, the reaction mixture was diluted with DCM and extracted with 1 M HCl. The organic phase was separated, dried over Na_2_SO_4_, filtered, and concentrated. The crude product was purified by gradient column chromatography with hexane/EtOAc eluent. Yield: 54.0 mg (37%, calculated from *tert*-butyl 3-oxobutanoate), yellow oil. ^1^H NMR (500 MHz, CDCl_3_) δ 7.74 (d, *J* = 8.3 Hz, 4H), 7.29 (d, *J* = 8.0 Hz, 4H), 5.28 (t, *J* = 6.1 Hz, 2H), 3.54 (t, *J* = 5.2 Hz, 4H), 3.22–3.09 (m, 4H), 2.41 (s, 6H), 2.20 (s, 3H), 1.45 (s, 9H). ^13^C{^1^H} NMR (126 MHz, CDCl_3_) δ 201.7, 164.2, 143.7, 137.0, 129.9, 127.2, 100.8, 84.8, 63.4, 42.8, 27.8, 26.3, 21.6. HRMS (ESI) *m*/*z*. [M + H]^+^ calcd for C_26_H_37_N_2_O_9_S_2_: 585.1935, found 585.1927.

### Methyl 2-cyano-2-(2-(4-methylphenylsulfonamido)ethoxy)propanoate (5f)

The 2-tosyl-1,2-oxazetidine (**1**, 53.3 mg, 0.25 mmol, 1.0 equiv), methyl 2-cyanopropanoate (28.3 mg, 0.25 mmol, 1.0 equiv), and K_2_CO_3_ (41.5 mg, 0.30 mmol, 1.2 equiv) in 1,4-dioxane (0.7 mL) was stirred for 2 days at room temperature. Then, the reaction mixture was diluted with DCM and extracted with 1 M HCl. The organic phase was separated, dried over Na_2_SO_4_, filtered, and concentrated. The crude product was purified by gradient column chromatography with hexane/EtOAc eluent. Yield: 36.7 mg (45%), colorless oil. ^1^H NMR (500 MHz, CDCl_3_) δ 7.75 (d, *J* = 8.3 Hz, 2H), 7.30 (d, *J* = 8.0 Hz, 2H), 5.09 (t, *J* = 5.7 Hz, 1H), 3.87 (s, 3H), 3.81–3.74 (m, 1H), 3.59–3.52 (m, 1H), 3.21 (dt, *J* = 5.3, 3.7 Hz, 2H), 2.42 (s, 3H), 1.73 (s, 3H). ^13^C{^1^H} NMR (126 MHz, CDCl_3_) δ 167.2, 143.7, 137.0, 129.9, 127.3, 116.0, 74.7, 67.3, 54.2, 42.6, 25.2, 21.6. HRMS (ESI) *m*/*z* [M + H]^+^ calcd for C_14_H_19_N_2_O_5_S: 327.1009, found 327.1002.

### Methyl 2-methyl-4-tosylmorpholine-2-carboxylate (6)

The methyl 3-hydroxy-2-methyl-4-tosylmorpholine-2-carboxylate (**3a′**, 175 mg, 0.53 mmol, 1.0 equiv) in DCM (5 mL) was cooled to 0 °C, and triethylsilane (185 mg, 1.59 mmol, 3.0 equiv) and BF_3_·Et_2_O (226 mg, 1.59 mmol, 3.0 equiv) were added dropwise under N_2_. After 15 min sat. NaHCO_3_ was added, the phases were separated, the aqueous was extracted 2× with DCM. The combined organic phases were dried over Na_2_SO_4_, filtered, and concentrated. The crude product was purified by gradient column chromatography with hexane/EtOAc eluent. Yield: 135 mg (81%), pale yellow solid, mp 99 °C. ^1^H NMR (500 MHz, CDCl_3_) δ 7.63 (d, *J* = 8.2 Hz, 2H), 7.34 (d, *J* = 7.9 Hz, 2H), 4.00–3.92 (m, 2H), 3.85–3.80 (m, 1H), 3.79 (s, 3H), 3.36 (dq, *J* = 11.5, 2.5 Hz, 1H), 2.55 (td, *J* = 11.0, 3.5 Hz, 1H), 2.44 (s, 3H), 2.34 (d, *J* = 11.5 Hz, 1H), 1.36 (s, 3H). ^13^C{^1^H} NMR (126 MHz, CDCl_3_) δ 172.3, 144.2, 132.6, 129.9, 128.0, 76.3, 62.8, 52.7, 52.4, 45.3, 23.4, 21.7. HRMS (ESI) *m*/*z* [M + H]^+^ calcd for C_14_H_20_NO_5_S: 314.1057, found 314.1049.

### Methyl 3-cyano-2-methyl-4-tosylmorpholine-2-carboxylate (7)

Methyl 3-hydroxy-2-methyl-4-tosylmorpholine-2-carboxylate (**3a′**, 100 mg, 0.30 mmol, 1.0 equiv) was dissolved in DCM (5 mL) and cooled to −78 °C. To this solution TMSCN (91 μL, 0.73 mmol, 2.4 equiv) and BF_3_·Et_2_O (92 μL, 0.73 mmol, 2.4 equiv) was added dropwise under N_2_ atmosphere. The reaction was stirred at −78 °C for 1 h, allowed to warm up to 0 °C and stirred for 2 h. Then, the reaction mixture was poured on sat. NaHCO_3_ solution and extracted with DCM. The organic phase was separated, dried over Na_2_SO_4_, filtered, concentrated. the crude product was purified by gradient column chromatography with hexane/EtOAc eluent. Yield: 64 mg (63%), colorless needle-shaped crystals, mp 139 °C. ^1^H NMR (300 MHz, CDCl_3_) δ 7.70 (d, *J* = 8.2 Hz, 2H), 7.37 (d, *J* = 8.1 Hz, 2H), 5.31 (s, 1H), 4.02 (td, *J* = 12.0, 3.2 Hz, 1H), 3.92–3.85 (m, 1H), 3.85 (s, 3H), 3.62–3.52 (m, 1H), 2.93 (td, *J* = 12.1, 3.8 Hz, 1H), 2.45 (s, 3H), 1.53 (s, 3H). ^13^C{^1^H} NMR (75 MHz, CDCl_3_) δ 169.9, 145.4, 132.8, 130.2, 128.2, 112.6, 78.0, 63.2, 53.4, 51.8, 41.3, 23.5, 21.8. HRMS (ESI) *m*/*z* [M + H]^+^ calcd for C_15_H_19_N_2_O_5_S: 339.1009, found 339.1004.

### 1-Ethyl 5-methyl (*E*)-4-methyl-4-(2-((4-methylphenyl)sulfonamido)ethoxy)pent-2-enedioate (8)

Methyl 3-hydroxy-2-methyl-4-tosylmorpholine-2-carboxylate (**3a′**, 3.50 g, 10.6 mmol, 1.0 equiv) and ethyl 2-(triphenyl-λ^5^-phosphaneylidene)acetate (4.81 g, 13.8 mmol, 1.3 equiv) was dissolved in DCM (150 mL) and heated on oil bath to reflux for 3 days. Then, the reaction mixture was concentrated, and the crude product was purified by gradient column chromatography with hexane/EtOAc eluent. Yield: 2.45 g (58%), yellow oil. ^1^H NMR (500 MHz, CDCl_3_) δ 7.73 (d, *J* = 8.2 Hz, 2H), 7.28 (d, *J* = 8.1 Hz, 2H), 6.88 (d, *J* = 15.8 Hz, 1H), 5.97 (d, *J* = 15.8 Hz, 1H), 5.39 (t, *J* = 5.5 Hz, 1H), 4.18 (q, *J* = 7.1 Hz, 2H), 3.75 (s, 3H), 3.53–3.43 (m, 2H), 3.18–3.06 (m, 2H), 2.39 (s, 3H), 1.46 (s, 3H), 1.28 (t, *J* = 7.1 Hz, 3H). ^13^C{^1^H} NMR (126 MHz, CDCl_3_) δ 172.0, 165.9, 145.9, 143.4, 137.0, 129.7, 127.2, 122.4, 79.9, 63.8, 60.8, 53.0, 43.2, 23.0, 21.5, 14.3. HRMS (ESI) *m*/*z* [M + H]^+^ calcd for C_18_H_26_NO_7_S: 400.1424, found 400.1421.

### Methyl 3-(2-ethoxy-2-oxoethyl)-2-methyl-4-tosylmorpholine-2-carboxylate (9)

Triethyl phosphonoacetate (75.0 mg, 0.33 mmol, 1.1 equiv) was diluted in 3 mL anhydrous THF, cooled to 0 °C and NaH (13.4 mg, 60% in mineral oil, 0.33 mmol, 1.1 equiv) was added under N_2_. After 15 min the solution of methyl 3-hydroxy-2-methyl-4-tosylmorpholine-2-carboxylate (**3a′**, 100.1 mg, 0.30 mmol, 1.0 equiv) in 3 mL anhydrous THF was added to the suspension and stirred for 4.5 h. Then 10 mL of water was added, and the resulting mixture was extracted with EtOAc. The combined organic phases were dried over Na_2_SO_4_, filtered, and concentrated. The crude product was purified by gradient column chromatography with hexane/EtOAc eluent. Yield: 73.6 mg (61%), pale yellow oil.

Compound **9** can be accessed in an alternative route: 1-ethyl 5-methyl (*E*)-4-methyl-4-(2-((4-methylphenyl)sulfonamido)ethoxy)pent-2-enedioate (**8**, 130 mg, 0.325 mmol, 1 equiv) and DBU (50 mg, 0.325 mmol, 49 μL, 1 equiv) was dissolved in 5 mL of THF, stirred at room temperature until completion (indicated by TLC), and then diluted with sat. aq. citric acid solution, extracted with EtOAc, and concentrated in vacuo. Yield 130 mg (quant.). ^1^H NMR (300 MHz, CDCl_3_) δ 7.67 (d, *J* = 8.0 Hz, 2H), 7.29 (d, *J* = 7.8 Hz, 2H), 4.95 (dd, *J* = 9.0, 2.8 Hz, 1H), 4.14 (q, *J* = 7.1 Hz, 2H), 4.04 (td, *J* = 12.2, 3.7 Hz, 1H), 3.76 (dd, *J* = 11.9, 3.8 Hz, 1H), 3.63 (s, 3H), 3.45 (dd, *J* = 12.8, 3.6 Hz, 1H), 2.98 (td, *J* = 12.7, 3.9 Hz, 1H), 2.80 (dd, *J* = 16.7, 8.9 Hz, 1H), 2.42 (s, 3H), 2.20 (dd, *J* = 16.8, 2.8 Hz, 1H), 1.25 (t, *J* = 7.1 Hz, 3H), 1.19 (s, 3H). ^13^C{^1^H} NMR (75 MHz, CDCl_3_) δ 172.2, 170.9, 143.9, 136.4, 129.8, 127.5, 79.3, 62.8, 61.1, 53.5, 52.4, 39.5, 32.0, 22.6, 21.6, 14.2. HRMS (ESI) *m*/*z* [M + H]^+^ calcd for C_18_H_26_NO_7_S: 400.1424, found 400.1421.

### Methyl 3-allyl-2-methyl-4-tosylmorpholine-2-carboxylate (10)

A solution of methyl 3-hydroxy-2-methyl-4-tosylmorpholine-2-carboxylate (264 mg, 0.80 mmol, 1.0 equiv, either **3a′** or **3a″**) and allyltrimethylsilane (510 μL, 3.20 mmol, 4.0 equiv) in DCM (16 mL) was cooled to −78 °C and BF_3_·Et_2_O (200 μL, 1.60 mmol, 2.0 equiv) was added dropwise under N_2_ atmosphere. The reaction was stirred for 1 h at −78 °C, allowed to warm up to room temperature, and stirred until all starting material was consumed (monitored by LC). The reaction was quenched by addition of sat. NaHCO_3_, diluted with DCM, and extracted with sat. NaHCO_3_. The organic phase was dried over Na_2_SO_4_, filtered, concentrated and the crude product was purified by gradient column chromatography with hexane/EtOAc eluent. Yield: 239 mg (85%), dr: 3.4/1, yellow oil. ^1^H NMR (500 MHz, CDCl_3_, major, **10′**) δ 7.65 (d, *J* = 8.3 Hz, 2H), 7.26 (d, *J* = 8.6 Hz, 2H), 5.81 (ddt, *J* = 17.1, 10.1, 7.0 Hz, 1H), 5.07 (dd, *J* = 17.0, 1.6 Hz, 1H), 5.02 (dd, *J* = 10.1, 1.5 Hz, 1H), 4.59 (t, *J* = 6.8 Hz, 1H), 3.91 (td, *J* = 12.1, 3.7 Hz, 1H), 3.69 (dd, *J* = 11.8, 3.8 Hz, 1H), 3.65 (s, 3H), 3.28 (ddt, *J* = 13.4, 3.8, 1.0 Hz, 1H), 3.10 (td, *J* = 12.9, 3.9 Hz, 1H), 2.54–2.46 (m, 1H), 2.40 (s, 3H), 2.35–2.26 (m, 1H), 1.28 (s, 3H). ^13^C{^1^H} NMR (126 MHz, CDCl_3_, major, **10′**) δ 173.0, 143.5, 137.8, 135.1, 129.6, 127.5, 117.6, 79.8, 62.8, 56.2, 52.4, 39.1, 30.9, 23.9, 21.6. ^1^H NMR (500 MHz, CDCl_3_, minor, **10″**) δ 7.65 (d, *J* = 8.4 Hz, 2H), 7.25 (d, *J* = 8.4 Hz, 2H), 5.46 (ddt, *J* = 17.1, 10.1, 7.1 Hz, 1H), 4.87 (dq, *J* = 17.1, 1.6 Hz, 1H), 4.79 (dq, *J* = 10.0, 1.4 Hz, 1H), 4.12 (t, *J* = 7.0 Hz, 1H), 3.86 (td, *J* = 12.2, 3.6 Hz, 1H), 3.76 (dd, *J* = 11.9, 4.1 Hz, 1H), 3.69 (s, 3H), 3.44 (dd, *J* = 13.5, 3.6 Hz, 1H), 3.08 (td, *J* = 12.9, 4.1 Hz, 1H), 2.39 (s, 3H), 2.37–2.29 (m, 1H), 2.23–2.15 (m, 1H), 1.56 (s, 3H). ^13^C{^1^H} NMR (126 MHz, CDCl_3_, minor, **10″**) δ 172.3, 143.5, 137.8, 134.2, 129.7, 127.2, 117.3, 77.5, 60.3, 57.5, 52.3, 39.1, 31.7, 21.5, 19.6. HRMS (ESI) *m*/*z* [M + H]^+^ calcd for C_17_H_24_NO_5_S: 354.1370, found 354.1367.

### 2-Methyl-4-tosylmorpholine-2-carboxylic acid (12)

Methyl 2-methyl-4-tosylmorpholine-2-carboxylate (**6**, 127 mg, 0.41 mmol, 1.0 equiv) and LiOH·H_2_O (51 mg, 1.22 mmol, 3.0 equiv) was dissolved in a 3:1 mixture of MeOH/H_2_O (2.4 mL) and heated in a heat block to 95 °C. The reaction was stirred until all starting material was consumed. Then, the reaction mixture was allowed to cool to room temperature and extracted 2× with Et_2_O. The aqueous phase was acidified to pH = 2–3 by 1 M HCl and extracted 3× with EtOAc. The combined organic phases were dried over Na_2_SO_4_, filtered, and concentrated. Yield: 112 mg (92%), white solid, mp 165 °C. ^1^H NMR (500 MHz, CDCl_3_) δ 7.65 (d, *J* = 8.2 Hz, 2H), 7.36 (d, *J* = 8.0 Hz, 2H), 4.01 (ddd, *J* = 12.4, 9.5, 3.1 Hz, 1H), 3.88 (dt, *J* = 12.0, 3.6 Hz, 1H), 3.78 (d, *J* = 11.6 Hz, 1H), 3.31–3.26 (m, 1H), 2.69 (ddd, *J* = 12.4, 9.7, 3.4 Hz, 1H), 2.54 (d, *J* = 11.6 Hz, 1H), 2.45 (s, 3H), 1.47 (s, 3H). ^13^C{^1^H} NMR (126 MHz, CDCl_3_) δ 176.2, 144.4, 132.3, 130.0, 128.1, 76.0, 62.8, 52.0, 45.3, 22.9, 21.7. HRMS (ESI) *m*/*z* [M + H]^+^ calcd for C_13_H_18_NO_5_S: 300.0900, found 300.0895.

### 4-(2-Methyl-4-tosylmorpholin-2-yl)butan-2-one (14)

The 2-methyl-4-tosylmorpholine-2-carboxylic acid (**12**, 111 mg, 0.37 mmol, 1.0 equiv), methyl vinyl ketone (34 μL, 0.41 mmol, 1.1 equiv), Na_2_CO_3_ (7.9 mg, 0.07 mmol, 0.2 equiv), and 10-methyl-9-(2,4,6-trimethylphenyl)acridinium perchlorate (**13**, 3.8 mg, 0.009 mmol, 0.025 equiv) was diluted in MeOH (2.5 mL) and irradiated with 440 nm 10 W LED for 3 days at room temperature. Then, the reaction mixture was concentrated, and the residue was purified by gradient column chromatography with hexane/EtOAc eluent. Yield: 83 mg (68%), yellow oil. ^1^H NMR (500 MHz, CDCl_3_) δ 7.58 (d, *J* = 8.1 Hz, 2H), 7.31 (d, *J* = 8.0 Hz, 2H), 3.69 (dddd, *J* = 32.4, 11.9, 6.5, 3.4 Hz, 2H), 3.01–2.95 (m, 1H), 2.82–2.77 (m, 1H), 2.77 (d, *J* = 11.2 Hz, 1H), 2.63 (d, *J* = 11.3 Hz, 1H), 2.45 (t, *J* = 7.8 Hz, 2H), 2.41 (s, 3H), 2.11 (s, 3H), 2.03–1.95 (m, 1H), 1.70–1.63 (m, 1H), 1.18 (s, 3H). ^13^C{^1^H} NMR (126 MHz, CDCl_3_) δ 208.1, 144.0, 132.4, 129.8, 127.8, 72.2, 60.1, 54.1, 45.7, 37.2, 30.7, 30.0, 21.6, 21.4. HRMS (ESI) *m*/*z* [M + H]^+^ calcd for C_16_H_24_NO_4_S: 326.1421, found 326.1414.

### 3-Allyl-2-methyl-4-tosylmorpholine-2-carboxylic acid (15′)

Methyl 3-allyl-2-methyl-4-tosylmorpholine-2-carboxylate (**10′**, 169.7 mg, 0.48 mmol, 1.0 equiv) and LiOH·H_2_O (60.4 mg, 1.44 mmol, 3.0 equiv) was dissolved in a 3:1 mixture of MeOH/H_2_O (6 mL), heated in a heat block to 95 °C, and the reaction was stirred for 2 h. Then, the reaction mixture was allowed to cool to room temperature, extracted 2× with Et_2_O. The aqueous phase was acidified to pH = 2–3 by 1 M HCl and extracted 3× with EtOAc. The combined organic phases were dried over Na_2_SO_4_, filtered, and concentrated. Yield: 162.9 mg (66%), white solid, mp 170 °C. ^1^H NMR (500 MHz, CDCl_3_) δ 9.72 (s, 1H), 7.69 (d, *J* = 8.3 Hz, 2H), 7.27 (d, *J* = 7.9 Hz, 2H), 5.85 (ddt, *J* = 17.1, 10.2, 6.9 Hz, 1H), 5.10 (dd, *J* = 17.0, 1.6 Hz, 1H), 5.07 (dd, *J* = 10.2, 1.5 Hz, 1H), 4.62 (t, *J* = 6.7 Hz, 1H), 3.94 (td, *J* = 12.2, 3.7 Hz, 1H), 3.75 (dd, *J* = 11.9, 3.7 Hz, 1H), 3.31 (dd, *J* = 13.3, 3.6 Hz, 1H), 3.14 (td, *J* = 12.8, 3.9 Hz, 1H), 2.64–2.49 (m, 1H), 2.40 (s, 3H), 2.37–2.28 (m, 1H), 1.39 (s, 3H). ^13^C{^1^H} NMR (126 MHz, CDCl_3_) δ 177.8, 143.7, 137.2, 135.0, 129.7, 127.6, 117.8, 79.7, 63.0, 56.0, 39.0, 31.0, 24.0, 21.6.

### 3-Allyl-2-methyl-4-tosylmorpholine-2-carboxylic acid (15″)

Methyl 3-allyl-2-methyl-4-tosylmorpholine-2-carboxylate (**10″**, 69.6 mg, 0.20 mmol, 1.0 equiv) and LiOH·H_2_O (14.8 mg, 0.59 mmol, 3.0 equiv) was dissolved in a 3:1 mixture of MeOH/H_2_O (2.7 mL), heated in a heat block to 95 °C and the reaction was stirred for 24 h. Then, the reaction mixture was allowed to cool to room temperature and extracted 2× with Et_2_O. The aqueous phase was acidified to pH = 2–3 by 1 M HCl and extracted 3× with EtOAc. The combined organic phases were dried over Na_2_SO_4_, filtered, and concentrated. Yield: 66.9 mg (94%), amorphous solid. ^1^H NMR (500 MHz, CDCl_3_) δ 9.16 (s, 1H), 7.67 (d, *J* = 8.3 Hz, 2H), 7.27 (d, *J* = 7.9 Hz, 2H), 5.45 (dddd, *J* = 16.6, 10.0, 7.9, 6.3 Hz, 1H), 4.91 (dq, *J* = 16.9, 1.6 Hz, 1H), 4.80–4.74 (m, 1H), 4.19 (dd, *J* = 9.4, 5.4 Hz, 1H), 3.92–3.86 (m, 1H), 3.80 (dd, *J* = 11.9, 4.0 Hz, 1H), 3.49 (dd, *J* = 13.9, 3.4 Hz, 1H), 3.11 (ddd, *J* = 13.8, 12.5, 4.1 Hz, 1H), 2.41 (s, 4H), 2.30–2.23 (m, 1H), 1.60 (s, 3H). ^13^C{^1^H} NMR (126 MHz, CDCl_3_) δ 174.8, 143.7, 137.8, 133.9, 129.7, 127.3, 117.9, 77.6, 60.9, 57.2, 38.9, 31.4, 21.6, 19.6. HRMS (ESI) *m*/*z* [M + H]^+^ calcd for C_16_H_22_NO_5_S: 340.1213, found 340.1209.

### 4-(3-Allyl-2-methyl-4-tosylmorpholin-2-yl)butan-2-one (16)

3-Allyl-2-methyl-4-tosylmorpholine-2-carboxylic acid (**15′**, 62.8 mg, 0.19 mmol, 1.0 equiv), methyl vinyl ketone (19 μL, 0.20 mmol, 1.1 equiv), Na_2_CO_3_ (3.9 mg, 0.04 mmol, 0.2 equiv), and 10-methyl-9-(2,4,6-trimethylphenyl)acridinium perchlorate (**13**, 1.9 mg, 0.005 mmol, 0.025 equiv) was diluted in MeOH (1.3 mL) and irradiated with LED (440 nm, 10 W) for 24 h at room temperature. Then the reaction mixture was concentrated, and the residue was purified by gradient column chromatography with hexane/EtOAc eluent. Yield: 57.1 mg (84%), yellow oil. Starting from **15″** under nearly identical conditions, except for 2 days of reaction time, the reaction yielded the same **16** product. Yield: 22.0 mg (33%). ^1^H NMR (500 MHz, CDCl_3_) δ 7.66 (d, *J* = 8.3 Hz, 2H), 7.27 (d, *J* = 8.0 Hz, 2H), 5.64 (ddt, *J* = 17.1, 10.1, 7.0 Hz, 1H), 4.95 (dd, *J* = 17.0, 1.6 Hz, 1H), 4.88 (dd, *J* = 10.1, 1.6 Hz, 1H), 3.74 (t, *J* = 6.8 Hz, 1H), 3.66 (td, *J* = 12.1, 3.5 Hz, 1H), 3.52 (dd, *J* = 11.8, 3.9 Hz, 1H), 3.40 (dd, *J* = 13.3, 3.4 Hz, 1H), 3.04 (td, *J* = 12.8, 4.0 Hz, 1H), 2.50–2.42 (m, 2H), 2.41 (s, 3H), 2.35–2.24 (m, 2H), 2.19 (ddd, *J* = 15.3, 10.6, 5.0 Hz, 1H), 2.13 (s, 3H), 1.79 (ddd, *J* = 14.4, 10.4, 5.5 Hz, 1H), 1.00 (s, 3H). ^13^C{^1^H} NMR (126 MHz, CDCl_3_) δ 207.9, 143.4, 138.0, 135.8, 129.7, 127.3, 117.1, 75.2, 59.9, 58.5, 39.3, 37.8, 31.6, 30.0, 27.7, 23.2, 21.6. HRMS (ESI) *m*/*z* [M + H]^+^ calcd for C_19_H_28_NO_4_S: 366.1734, found 366.1728.

## Data Availability

The data underlying this study are available in the published article and its Supporting Information.

## Supporting Information Available

Reaction optimizations, ^1^H and ^13^C{^1^H} NMR spectra for all new compounds, as well as XRD data for **3c**, **7** (PDF)
FAIR data, including the primary NMR FID files, for compounds **1**, **2a**, **2d**, **2f**,**g**, **2i**–**l**, **3a′**, **3a″**, **3b**–**d**, **3e′**, **3e″**, **3f**–**h**, **3i′**, **3i″**, **3j′**, **3j″**, **3k**,**l**, **5a**–**f**, **6**–**9**, **10′**, **10″**, **12**, **14**, **15′**, **15″**, **16** (ZIP)

## References

[ref1] aArshad F.; Khan M. F.; Akhtar W.; Alam M. M.; Nainwal L. M.; Kaushik S. K.; Akhter M.; Parvez S.; Hasan S. M.; Shaquiquzzaman M. Revealing quinquennial journey of morpholine: a SAR based review. Eur. J. Med. Chem. 2019, 167, 324–356. 10.1016/j.ejmech.2019.02.015. 30776694

[ref2] Lenci E.; Calugi L.; Trabocchi A. Occurrence of Morpholine in Central Nervous System Drug Discovery. ACS Chem. Neurosci. 2021, 12, 378–390. 10.1021/acschemneuro.0c00729. 33459557 PMC7877733

[ref3] aPal’chikov V. Morpholine. Synthesis and Biological Activity. Russ. J. Org. Chem. 2013, 49, 787–814. 10.1134/S1070428013060018.

[ref4] aYar M.; McGarrigle E. M.; Aggarwal V. K. An Annulation Reaction for the Synthesis of Morpholines, Thiomorpholines, and Piperazines from β-Heteroatom Amino Compounds and Vinyl Sulfonium Salts. Angew. Chem., Int. Ed. 2008, 47, 3784–3786. 10.1002/anie.200800373. 18404756

[ref5] aDugar S.; Sharma A.; Kuila B.; Mahajan D.; Dwivedi S.; Tripathi V. A Concise and Efficient Synthesis of Substituted Morpholines. Synthesis 2015, 47, 712–720. 10.1055/s-0034-1379641.

[ref6] aJavorskis T.; Sriubaitė S.; Bagdžiu̅nas G.; Orentas E. N-Protected 1,2-Oxazetidines as a Source of Electrophilic Oxygen: Straightforward Access to Benzomorpholines and Related Heterocycles by Using a Reactive Tether. Chem. Eur. J. 2015, 21, 9157–9164. 10.1002/chem.201500731. 25959630

[ref7] aDegorce S. L.; Bodnarchuk M. S.; Cumming I. A.; Scott J. S. Lowering Lipophilicity by Adding Carbon: One-Carbon Bridges of Morpholines and Piperazines. J. Med. Chem. 2018, 61, 8934–8943. 10.1021/acs.jmedchem.8b01148. 30189136

[ref8] aNeipp C. E.; Martin S. F. A ring-closing olefin metathesis approach to bridged azabicyclic structures. Tetrahedron Lett. 2002, 43, 1779–1782. 10.1016/S0040-4039(02)00100-4.

[ref9] aJuaristi E.; Bandala Y. Anomeric Effect in Saturated Heterocyclic Ring Systems. Adv. Heterocycl. Chem. 2012, 105, 189–222. and references cited therein 10.1016/B978-0-12-396530-1.00002-4.

[ref10] For details, see the Supporting Information.

[ref11] Bandar J. S.; Lambert T. H. Enantioselective Brønsted Base Catalysis with Chiral Cyclopropenimines. J. Am. Chem. Soc. 2012, 134, 5552–5555. 10.1021/ja3015764. 22417078

[ref12] aSi C.-M.; Huang W.; Du Z.-T.; Wei B.-G.; Lin G.-Q. Diastereoconvergent Synthesis of trans-5-Hydroxy-6-Substituted-2-Piperidinones by Addition–Cyclization–Deprotection Process. Org. Lett. 2014, 16, 4328–4331. 10.1021/ol5020812. 25083821

[ref13] aAyala L.; Lucero C. G.; Romero J. A. C.; Tabacco S. A.; Woerpel K. A. Stereochemistry of Nucleophilic Substitution Reactions Depending upon Substituent: Evidence for Electrostatic Stabilization of Pseudoaxial Conformers of Oxocarbenium Ions by Heteroatom Substituents. J. Am. Chem. Soc. 2003, 125, 15521–15528. 10.1021/ja037935a. 14664599

[ref14] aKanegusuku A. L. G.; Roizen J. L. Recent Advances in Photoredox-Mediated Radical Conjugate Addition Reactions: An Expanding Toolkit for the Giese Reaction. Angew. Chem., Int. Ed. 2021, 60, 21116–21149. 10.1002/anie.202016666. PMC8382814 33629454

[ref15] aSchnermann M. J.; Overman L. E. A Concise Synthesis of (−)-Aplyviolene Facilitated by a Strategic Tertiary Radical Conjugate Addition. Angew. Chem., Int. Ed. 2012, 51, 9576–9580. 10.1002/anie.201204977. PMC3517069 22926995

[ref16] El-Mansy M. F.; Kang J. Y.; Lingampally R.; Carter R. G. Proline Sulfonamide-Catalyzed, -Domino Process for Asymmetric Synthesis of Amino- and Hydroxy-Substituted Bicyclo[2.2.2]octanes. Eur. J. Org. Chem. 2016, 2016, 150–157. 10.1002/ejoc.201501302.

[ref17] Shimada N.; Stewart C.; Bow W. F.; Jolit A.; Wong K.; Zhou Z.; Tius M. A. Neutral Nazarov-Type Cyclization Catalyzed by Palladium(0). Angew. Chem., Int. Ed. 2012, 51, 5727–5729. 10.1002/anie.201201724. PMC3445627 22539448

[ref18] Nakatsuji H.; Nishikado H.; Ueno K.; Tanabe Y. General, Robust, and Stereocomplementary Preparation of α,β-Disubstituted α,β-Unsaturated Esters. Org. Lett. 2009, 11 (19), 4258–4261. 10.1021/ol9013359. 19715286

[ref19] Drouin M.; Tremblay S.; Paquin J. F. Palladium-catalyzed synthesis of monofluoroalkenes from 3,3-difluoropropenes using dimethylmalonate and derivatives as nucleophiles. Org. Biomol. Chem. 2017, 15 (11), 2376–2384. 10.1039/C7OB00376E. 28244537

[ref20] Kido F.; Noda Y.; Yoshikoshi A. New access to dl-paniculide using α-phenylthio-β-vinylbutenolide as synthetic block. Tetrahedron 1987, 43 (23), 5467–5474. 10.1016/S0040-4020(01)87730-9.

[ref21] Croxall W. J.; van Hook J. O. Transetherification Reactions. Preparation and Rearrangement of β-Alloxyacrylates. J. Am. Chem. Soc. 1950, 72 (2), 803–808. 10.1021/ja01158a040.

[ref22] Francis B.; Martin D.; Robin L. G.; Huifen C.; Daniel S.; Elisia V.; Matthew V.; Baihua H.; Aijun L.; Andrew C.; Stuart W. Oxadiazolones as transient receptor potential channel inhibitors. WO2018096159A1, 2018.

