# Supplementary material for: Syntheses of 2- and 3-Substituted Morpholine Congeners via Ring Opening of 2-Tosyl-1,2-Oxazetidine
Source: J Org Chem. 2023 Apr 26;88(9):6182–91. doi: 10.1021/acs.joc.3c00207 (PMC10167689; doi:10.1021/acs.joc.3c00207)
Supplement: Supplementary file 1 — jo3c00207_si_001.pdf [file jo3c00207_si_001.pdf]

Supporting Information

Syntheses of 2- and 3-Substituted Morpholine Congeners via Ring Opening of 2-Tosyl-1,2-Oxazetidine

Bálint Kőnig,[†,‡] Gábor Sztanó,[†,‡] Tamás Holczbauer,[†,#] Tibor Soós[†,*]

[†] Institute of Organic Chemistry, Research Centre for Natural Sciences, 2 Magyar tudósok krt 2., H-1117 Budapest, Hungary

[‡] Hevesy György PhD School of Chemistry, Eötvös Loránd University, 1/A Pázmány Péter sétány, H-1117 Budapest, Hungary

Centre for Structural Science, Research Centre for Natural Sciences, 2 Magyar tudósok krt, H-1117 Budapest, Hungary

Email: soos.tibor@ttk.hu

Content

General information S3
Optimization of solvent, reaction temperature and concentration S4
Details of the light source........ S5
NMR spectra S6
X-ray data........ S54

General information

All starting materials were used without further purification unless stated otherwise. Anhydrous THF and toluene were distilled from sodium/benzophenone. Melting points were determined with SRS MPA100 apparatus and are uncorrected. Exact mass measurements were performed on a high-resolution hybrid quadrupole-time-of-flight mass spectrometer (Waters Select Series IMS, Waters Corp., Wilmslow, U.K.) equipped with Z-spray electrospray ionization source. Samples were dissolved in acetonitrile-water 1:1 (V/V) solvent mixture containing 0.1% (V/V) formic acid. Solutions were directly introduced into the ion source using a syringe pump. Under the applied conditions, the compounds form protonated, $[M+H]^+$ and sodiated, $[M+Na]^+$ molecules in positive ionization ESI. X-ray crystallography was performed on a Rigaku RAXIS-RAPID II diffractometer. NMR spectra were recorded on Varian 300 and Varian 500 spectrometer. ^{13}C NMR spectra were acquired in broad-band continuous decoupled mode. The chemical shifts (δ) are given in parts per million (ppm) using solvent residual as an internal reference. Abbreviations: singlet (s), doublet (d), triplet (t), quartet (q), heptet (hept), doublet of doublets (dd), triplet of doublets (td), doublet of triplets (dt), double doublet of doublets (ddd), double triplet of doublets (dtd), double doublet of triplets (ddt), doublet of doublet of doublet of doublets (dddd), multiplet (m) and broad (br). Flash column chromatography was carried out with a Teledyne ISCO CombiFlash Rf200 and Teledyne ISCO CombiFlash Nextgen 300+ systems, using RediSep® Rf Gold columns and gradient elution program with hexane/EtOAc. Thin-layer chromatography (TLC) was carried out using Merck TLC Silica gel 60 F_{254} precoated plates.

Optimization of solvent, reaction temperature and concentration

Representative procedure

2-tosyl-1,2-oxazetidine (**1**, 53 mg, 0.25 mmol, 1.0 equiv.), methyl 2-methyl-3-oxopropanoate (**2a**, 29 mg, 0.25 mmol, 1 equiv.), DBU (46 mg, 45 µl, 0.30 mmol, 1.2 equiv.) and 1,3,5-trimethoxybenzene internal standard (13 mg, 0.075 mmol, 0.3 equiv.) were dissolved in 0.5 ml of solvent. The reaction mixture was stirred for 18 hours at the indicated temperature, then evaporated to dryness The conversion was determined by qNMR using 1,3,5-trimethoxy benzene internal standard.

Table S1

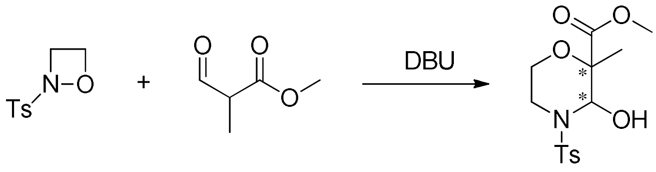

#	Solvent	Temp.	Conc.	Total conv.	d.r.
1	Toluene	r.t.	0.5 M	67%	3.24
2	Chloroform	r.t.	0.5 M	48%	3.04
3	DCE	r.t.	0.5 M	53%	3.00
4	DCM	r.t.	0.5 M	55%	2.59
5	Hexane	r.t.	0.5 M	57%	2.78
6	MTBE	r.t.	0.5 M	69%	3.02
7	THF	r.t.	0.5 M	53%	3.15
8	THF:H_2O 3:2	r.t.	0.5 M	24%	2.87
9	Pyridine	r.t.	0.5 M	60%	2.79
10	1,4-Dioxane	r.t.	0.5 M	59%	3.23
11	MeCN	r.t.	0.5 M	47%	3.41
12	2-Propanol	r.t.	0.5 M	62%	1.22
13	EtOAc	r.t.	0.5 M	55%	3.26
14	Toluene	50	0.5 M	66%	3.33
15	Toluene	110	0.5 M	3%	2.17
16	Toluene	r.t.	2.5 M	65%	3.60

Details of the light source

Manufacturer: Shenzhen Weili Optical Co., Ltd
Model number: G-10P45V140A1-BV
LED power: 10 W
Peak wavelength: 439 nm
Peak width at half-heigth: 20 nm

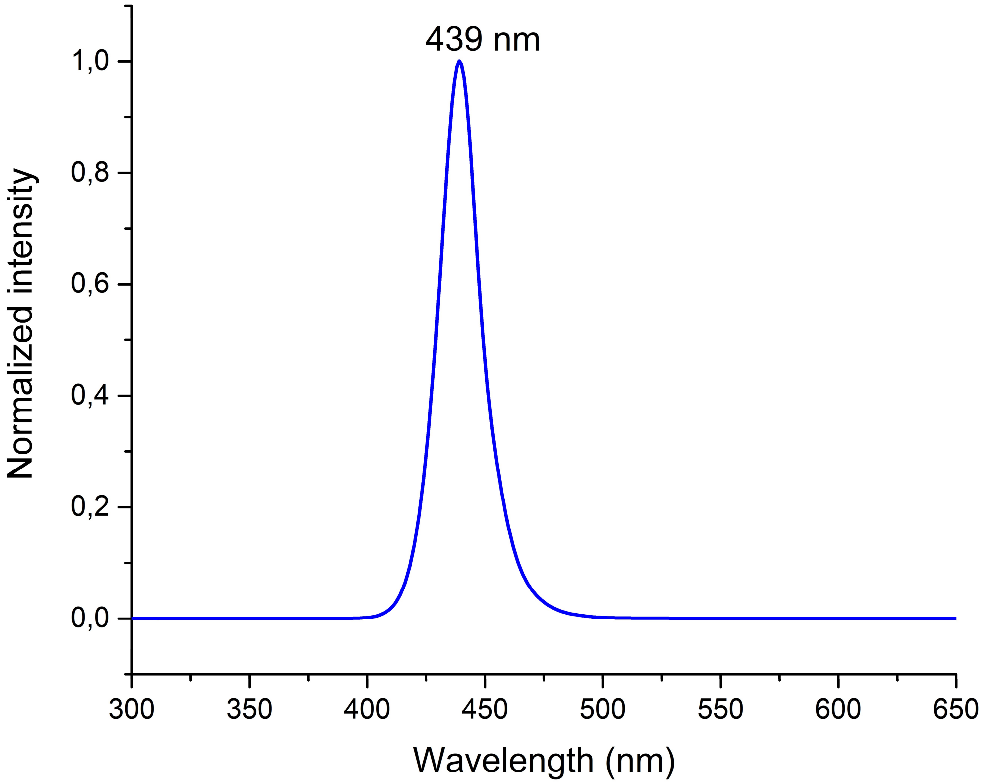

NMR spectra

^{1}H NMR of **1** (300 MHz, $CDCl_3$)

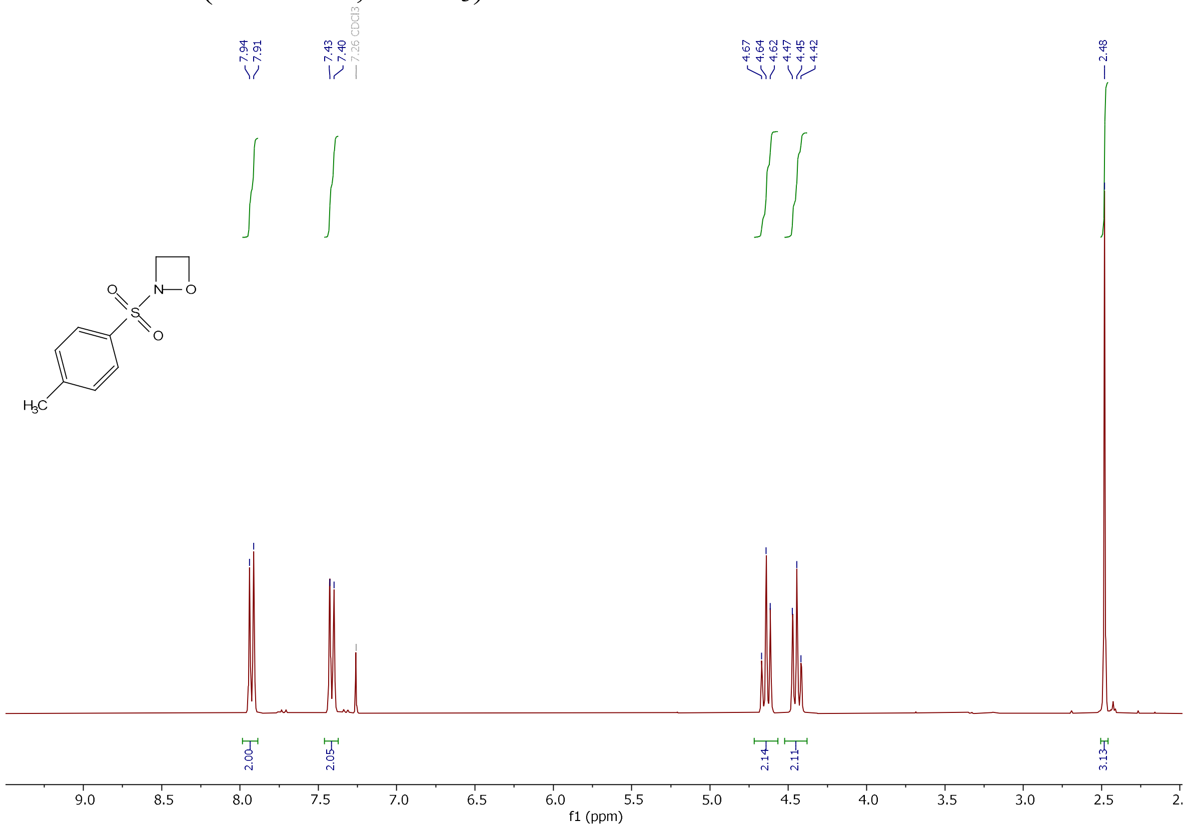

^{13}C{^{1}H} NMR of **1** (75 MHz, $CDCl_3$)

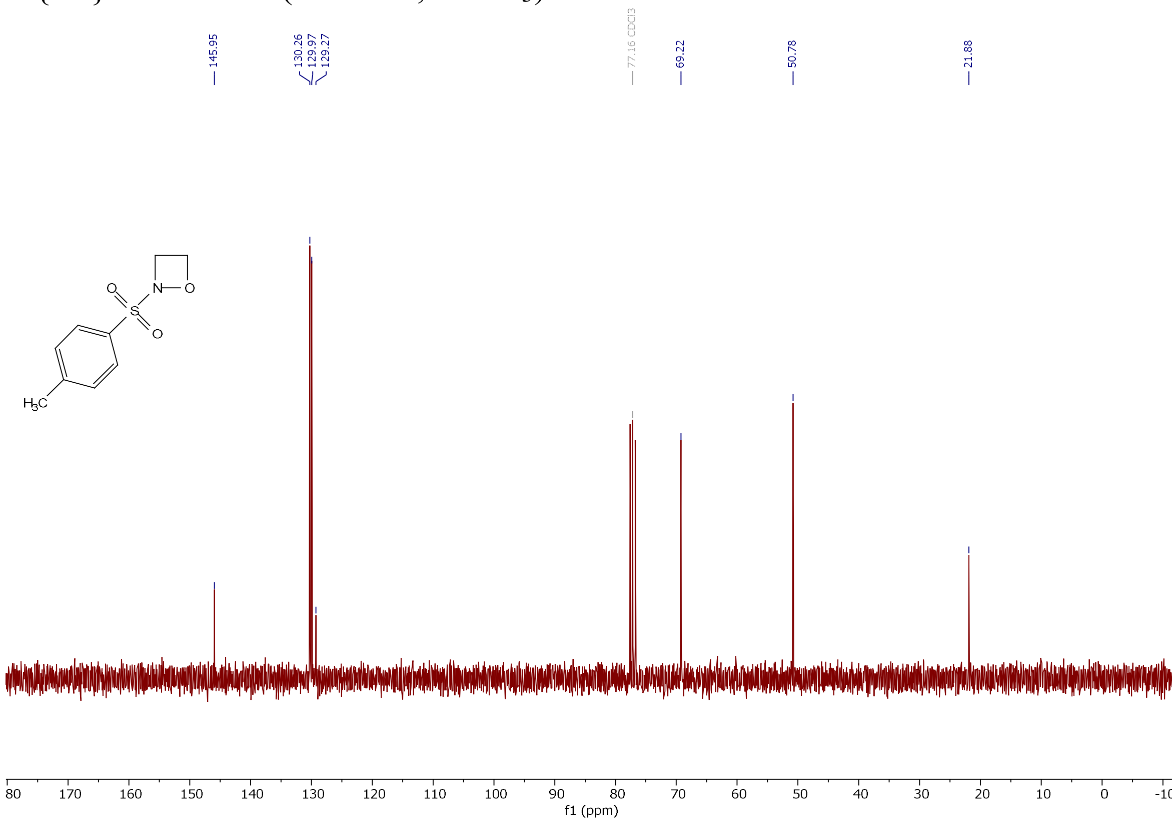

^{1}H NMR of **2a** (300 MHz, $CDCl_3$)

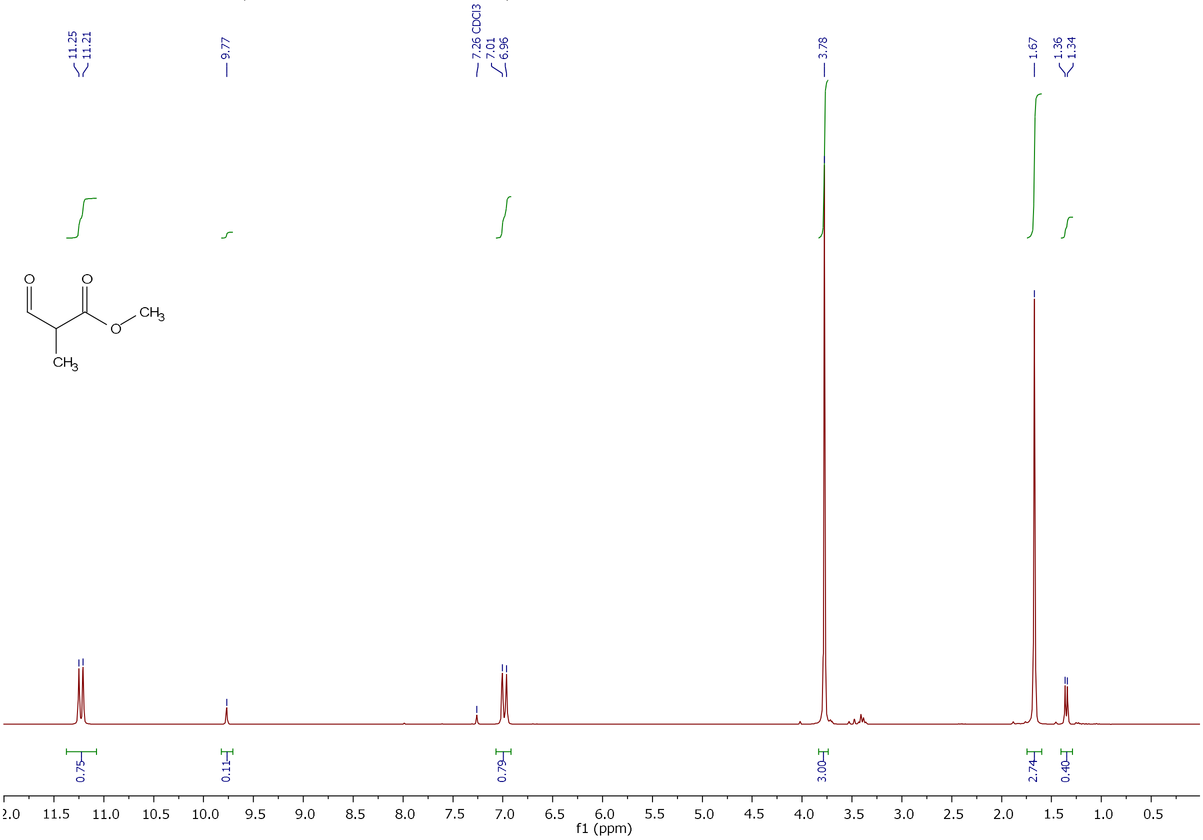

^{13}C{^{1}H} NMR of **2a** (75 MHz, $CDCl_3$)

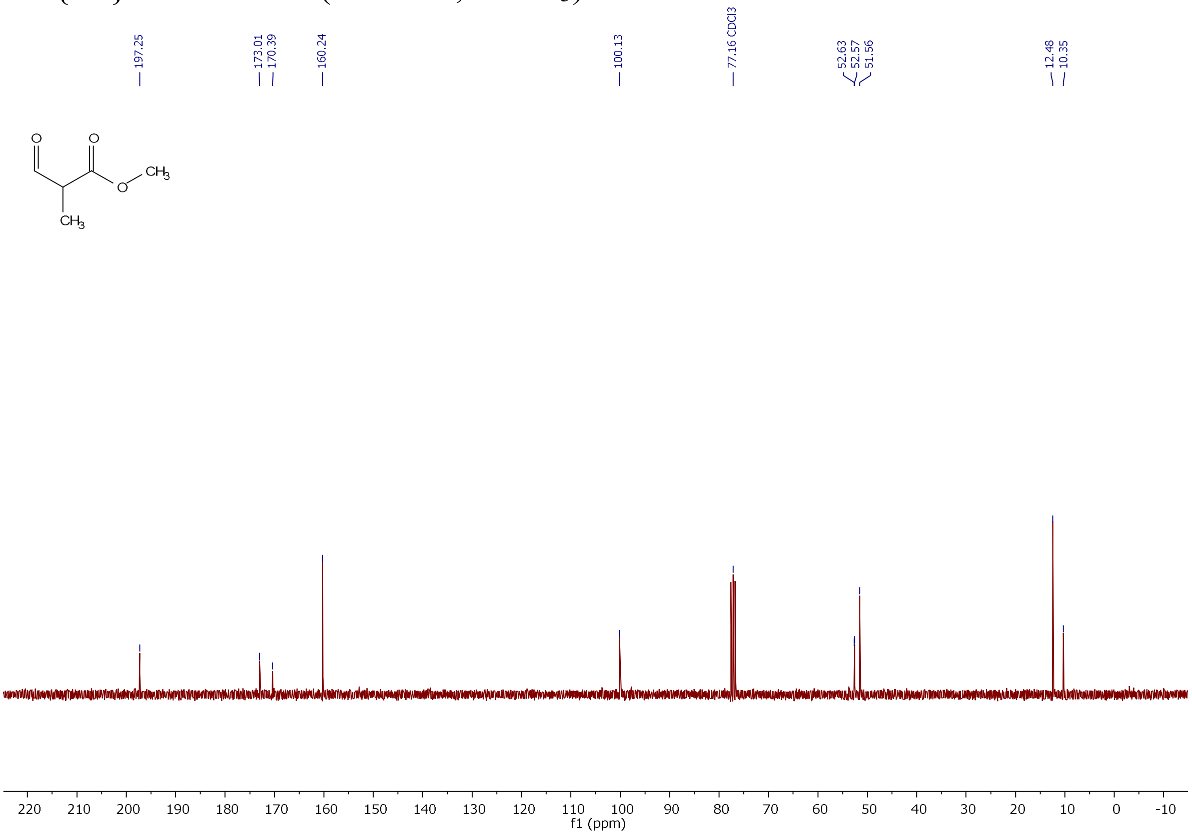

^{1}H NMR of **2d** (500 MHz, DMSO-d_6)

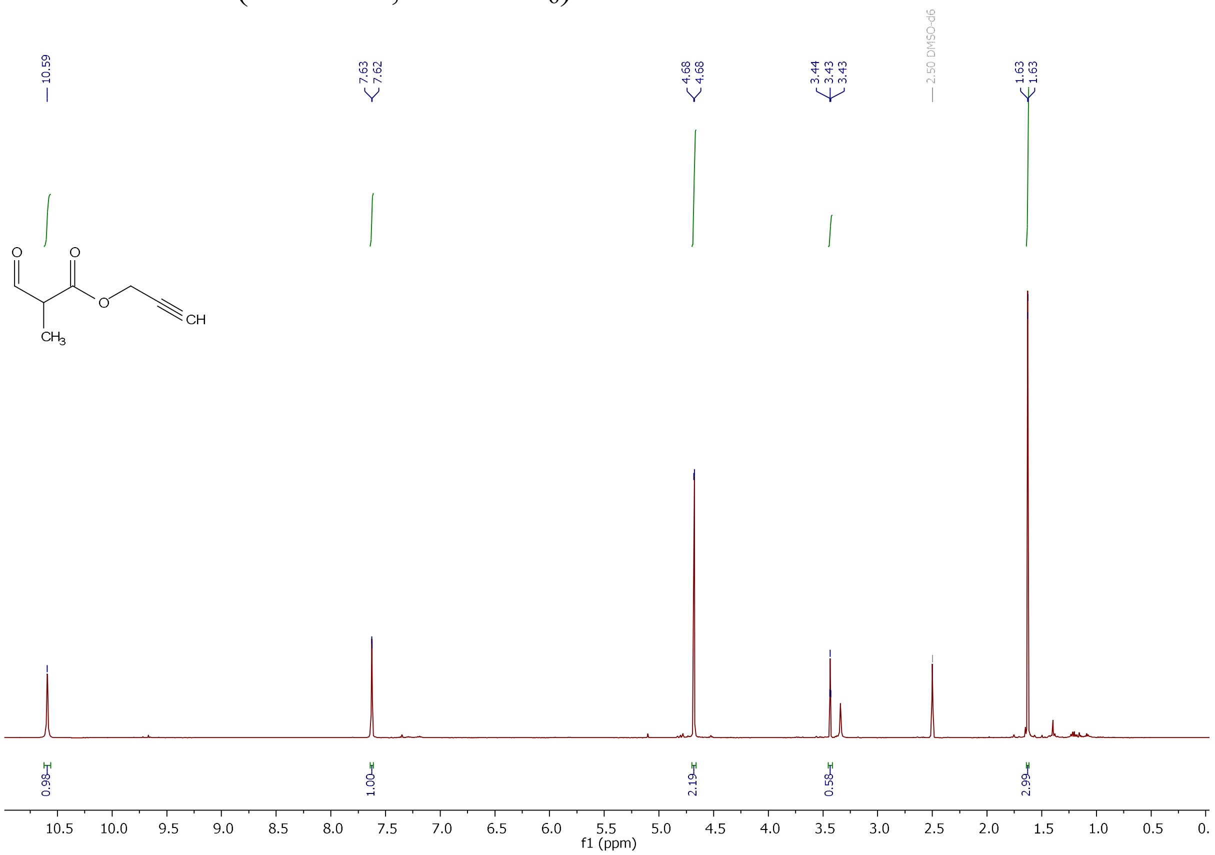

^{13}C{^{1}H} NMR of **2d** (126 MHz, DMSO-d_6)

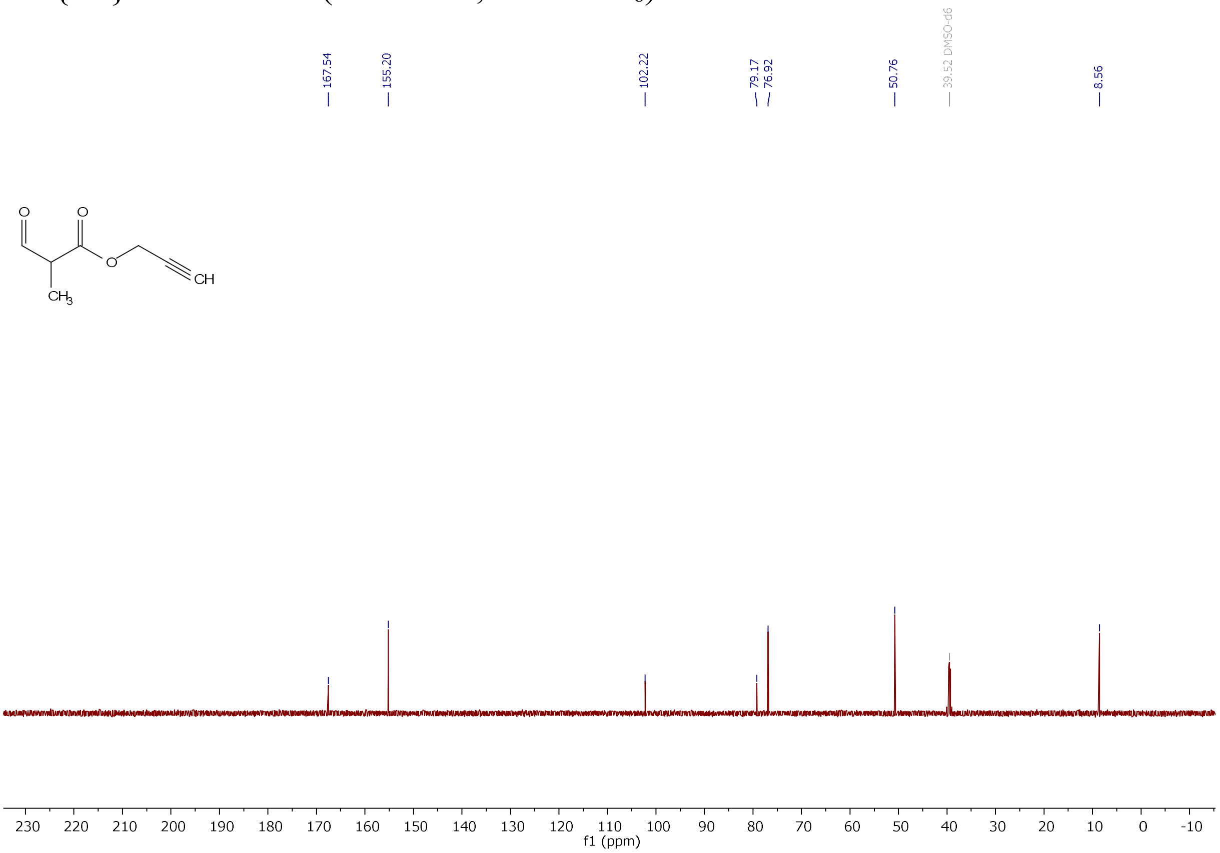

^{1}H NMR of **2f** (500 MHz, DMSO-d_6)

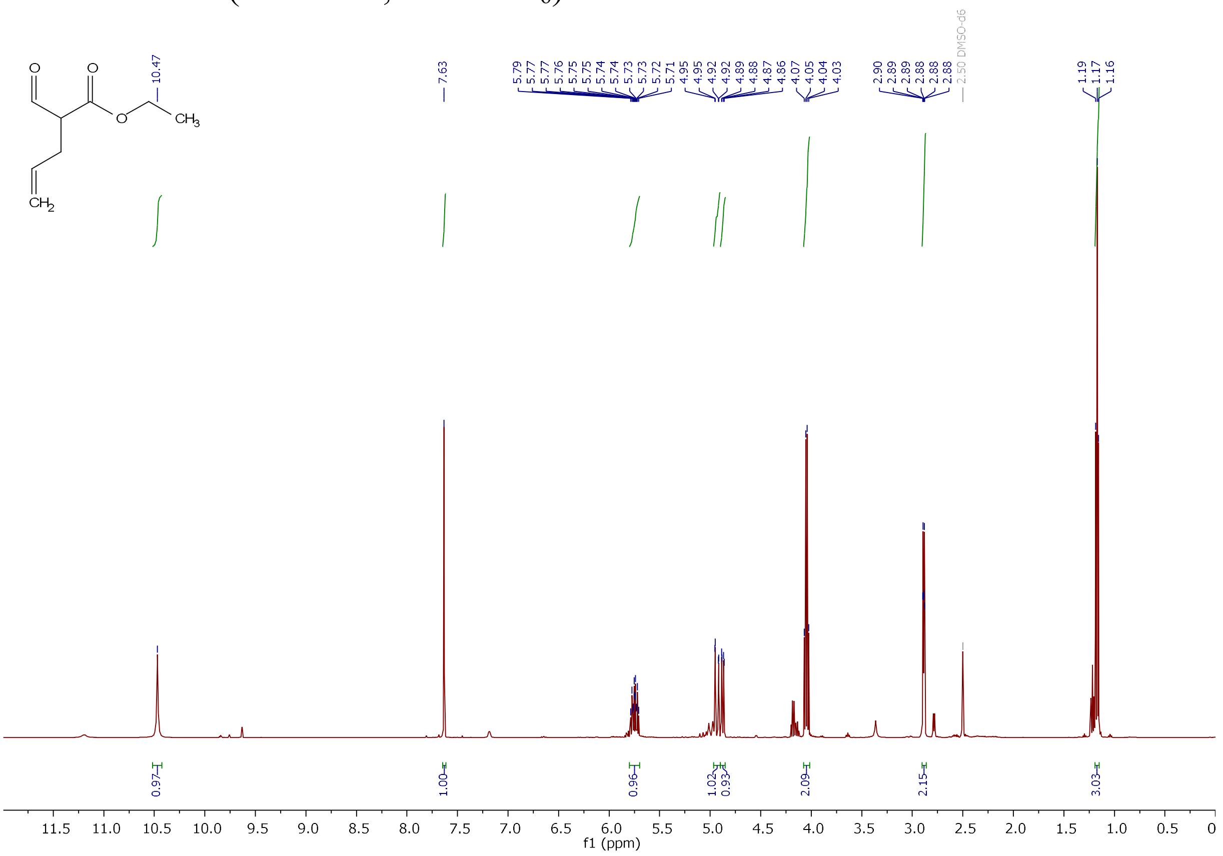

^{13}C{^{1}H} NMR of **2f** (126 MHz, DMSO-d_6)

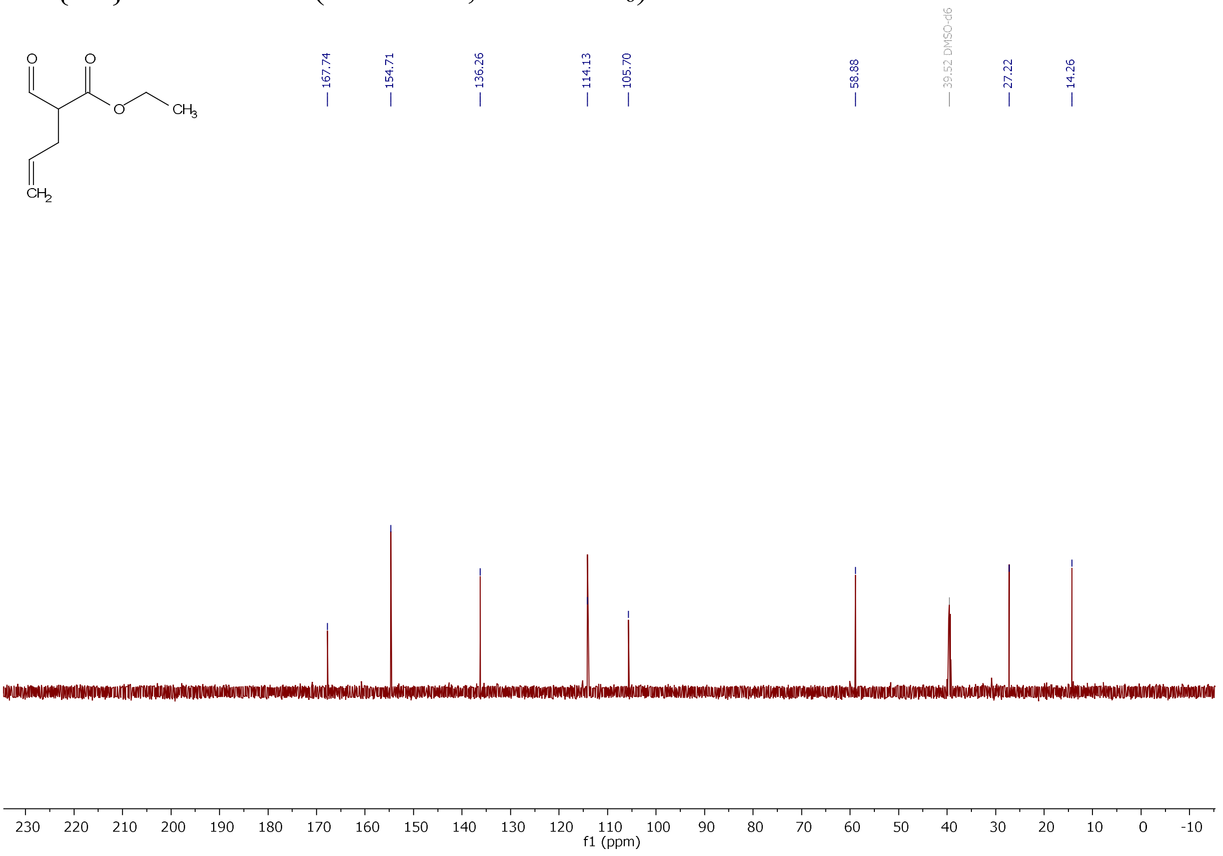

^{1}H NMR of **2g** (500 MHz, $CDCl_3$)

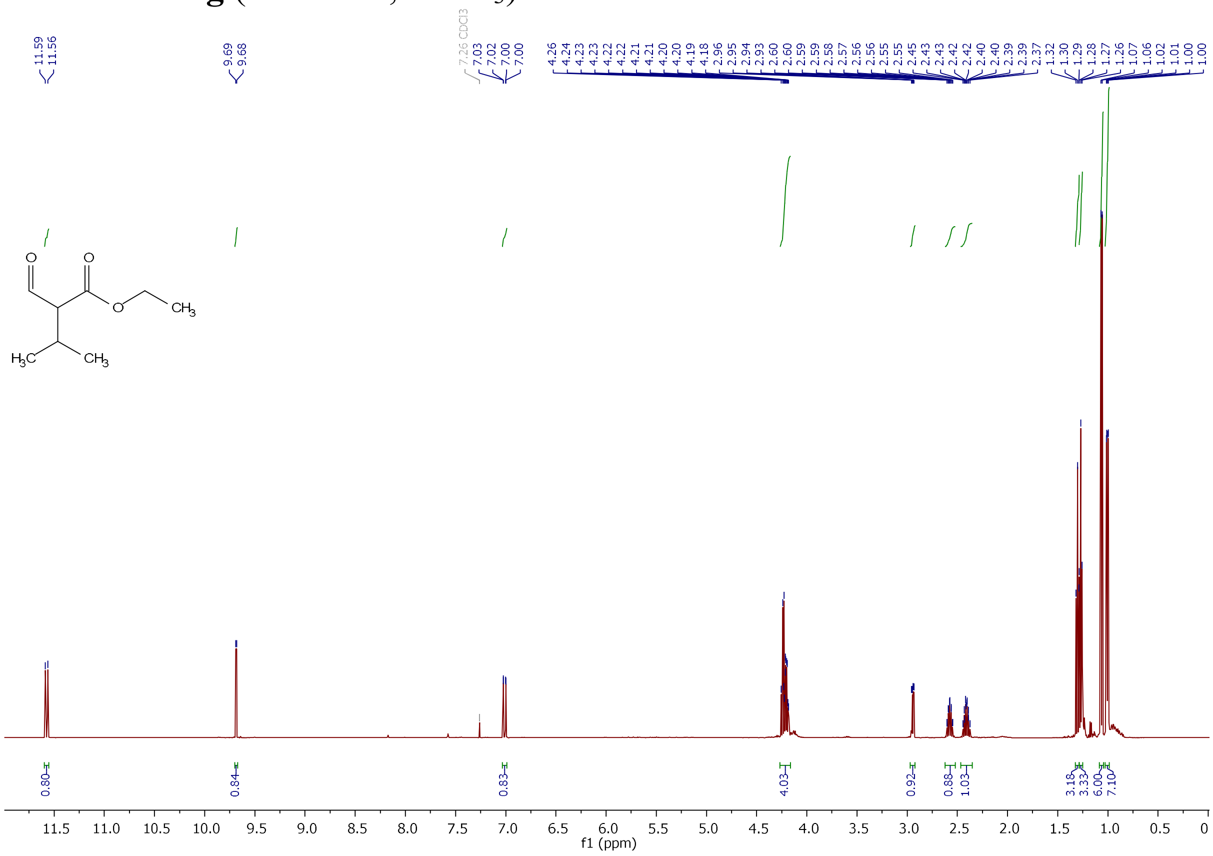

^{13}C{^{1}H} NMR of **2g** (126 MHz, $CDCl_3$)

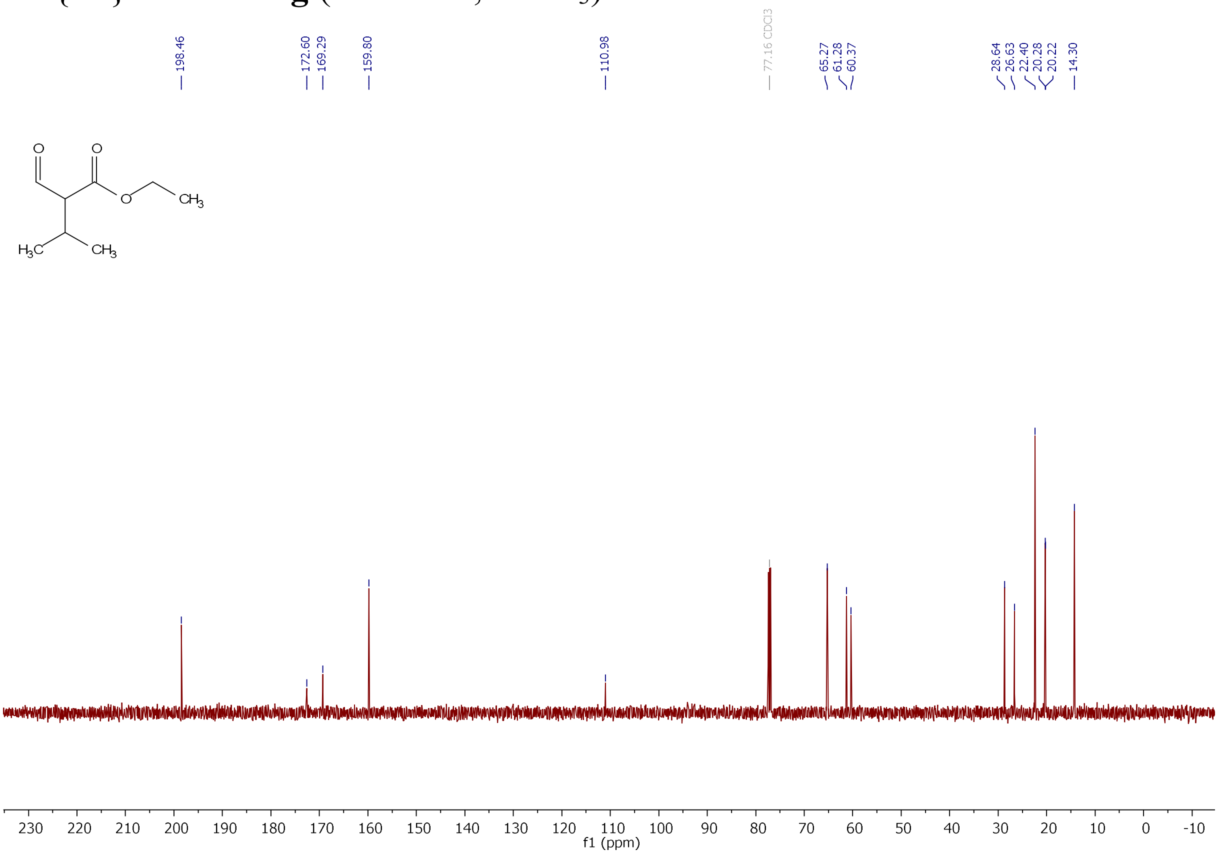

^{1}H NMR of **2i** (500 MHz, DMSO-d_6)

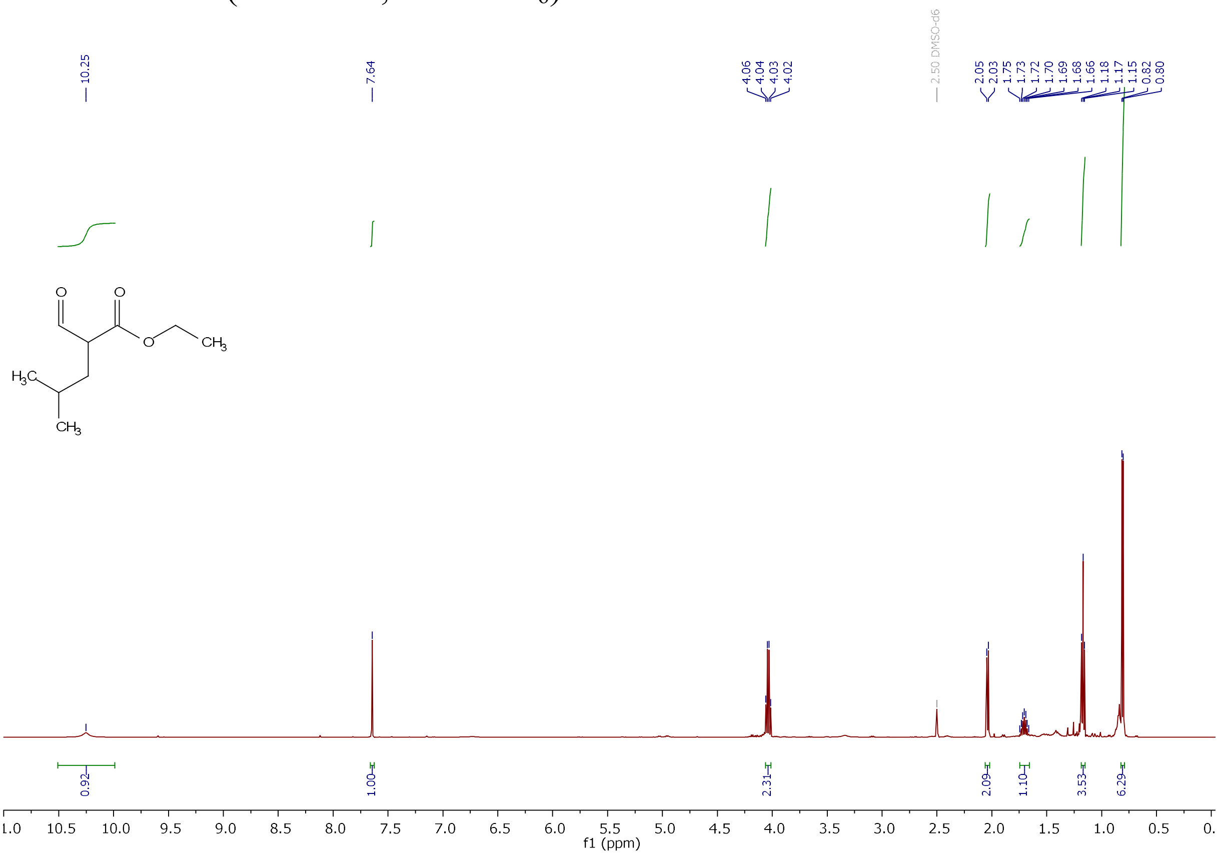

^{13}C{^{1}H} NMR of **2i** (126 MHz, DMSO-d_6)

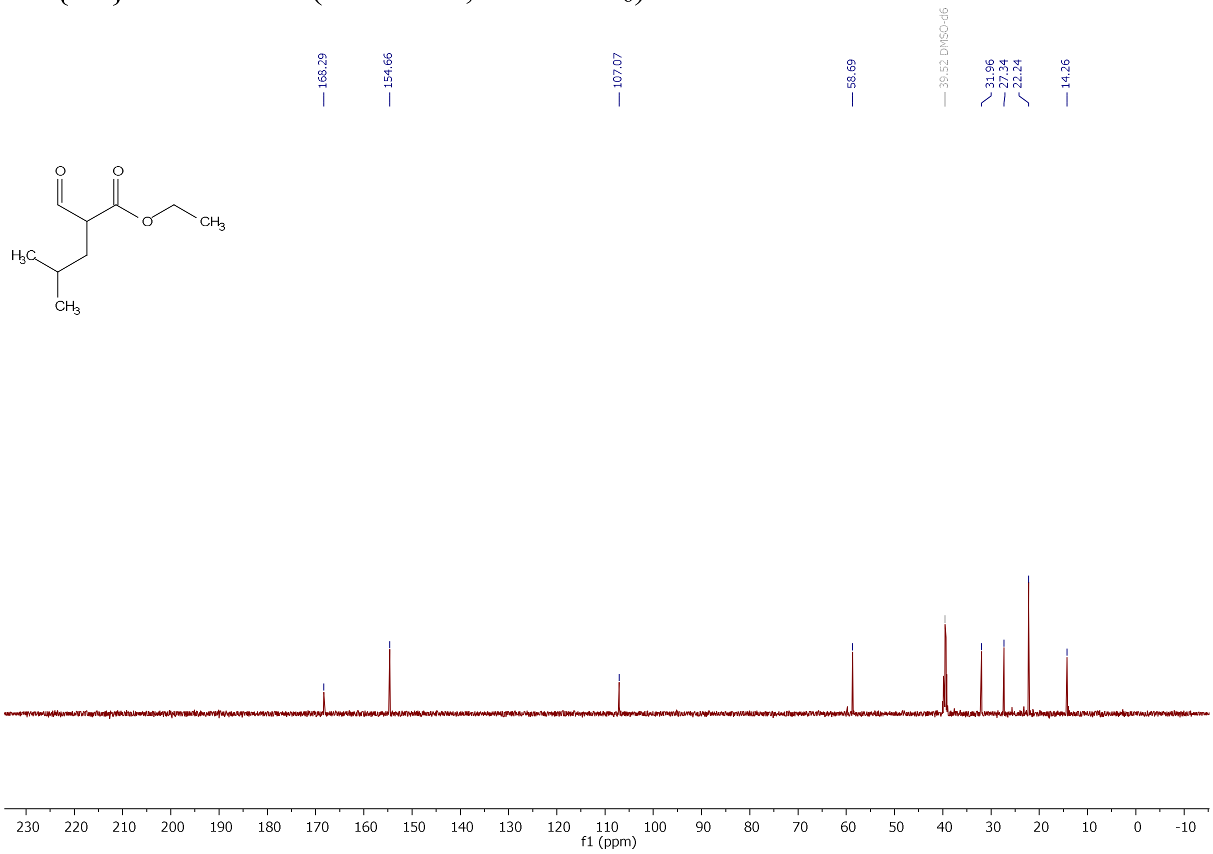

^{1}H NMR of **2j** (500 MHz, DMSO-d_6)

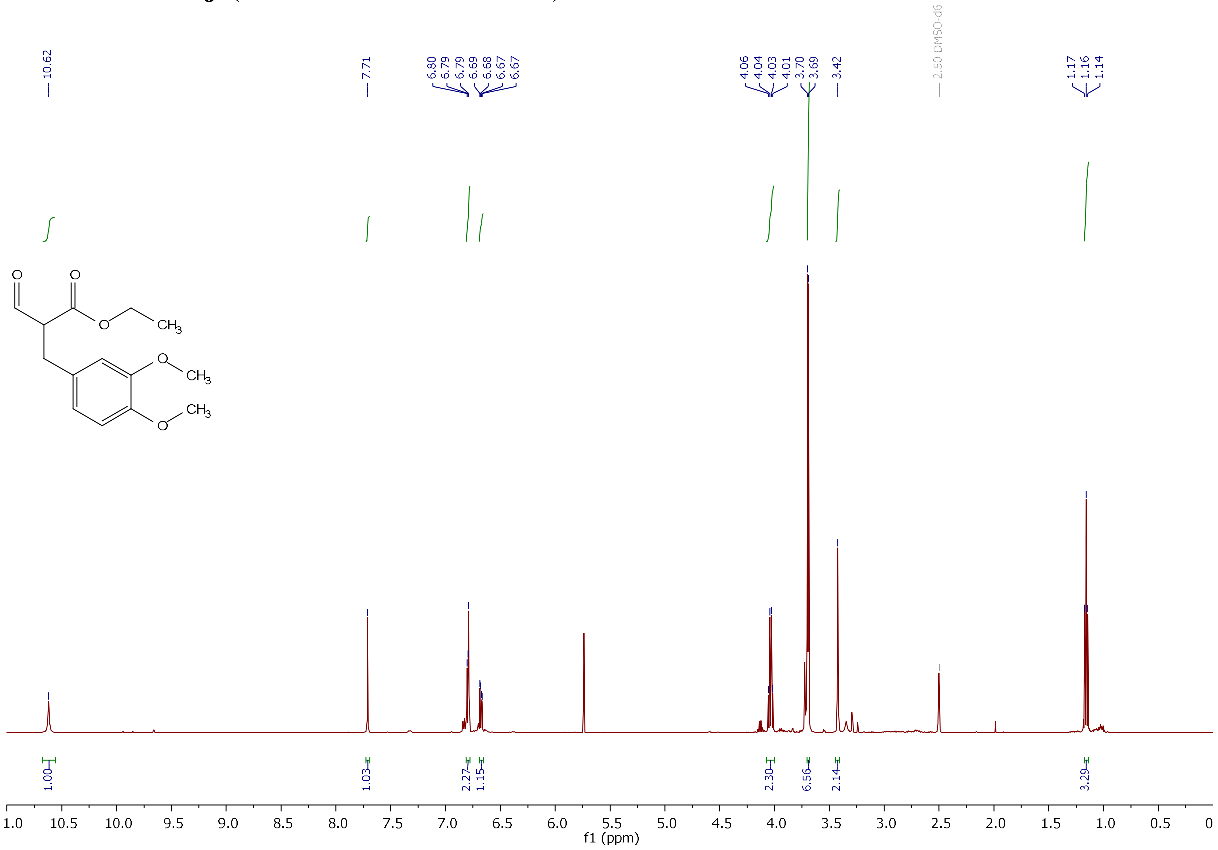

$^{13}C\{^{1}H\}$ NMR of **2j** (126 MHz, DMSO-d_6)

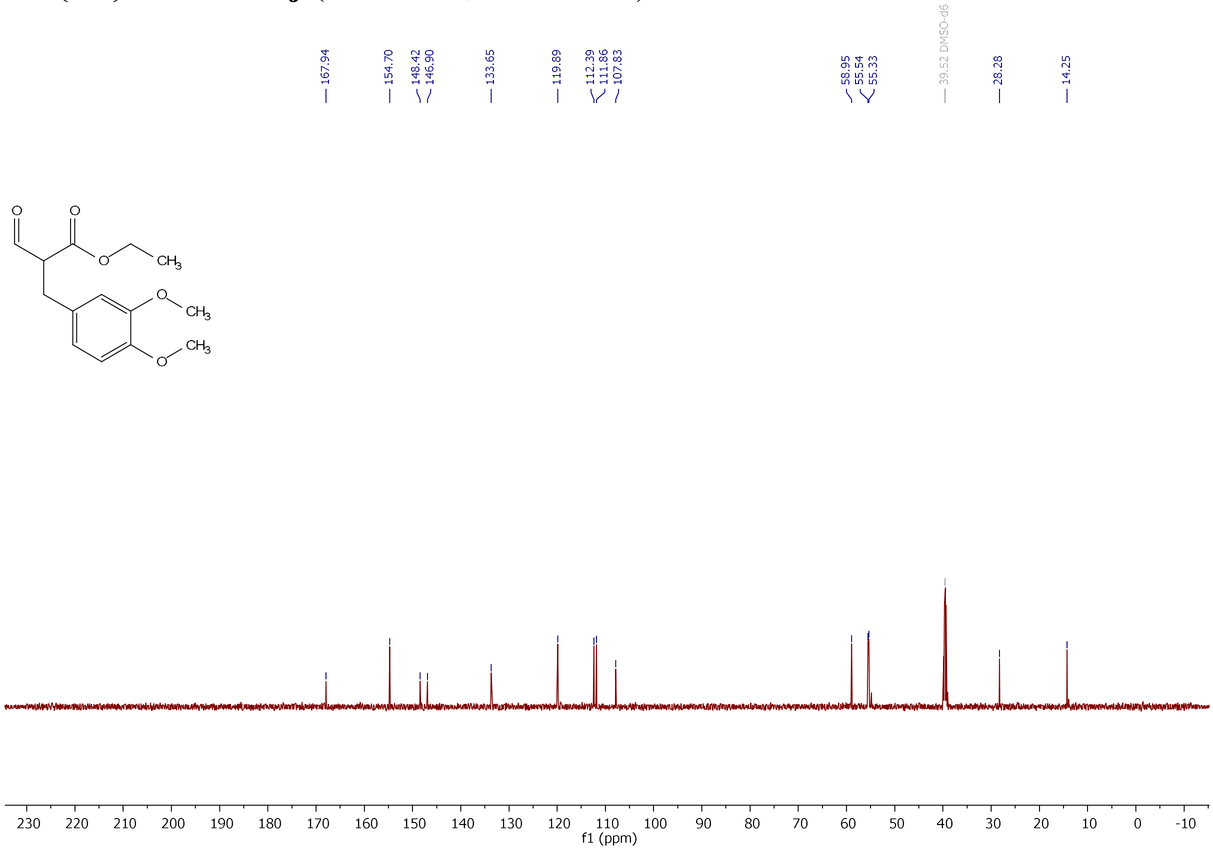

^{1}H NMR of **2k** (500 MHz, $CDCl_3$)

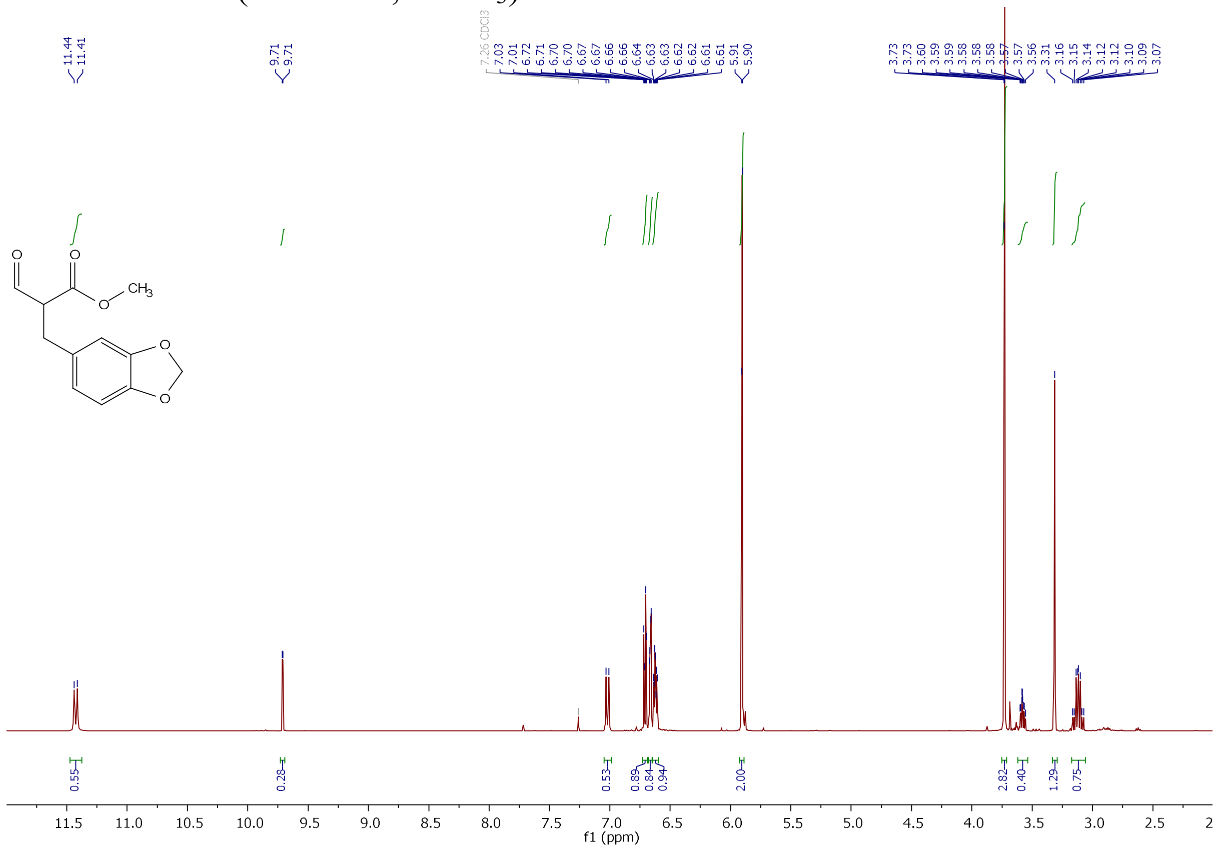

$^{13}C\{^{1}H\}$ NMR of **2k** (126 MHz, $CDCl_3$)

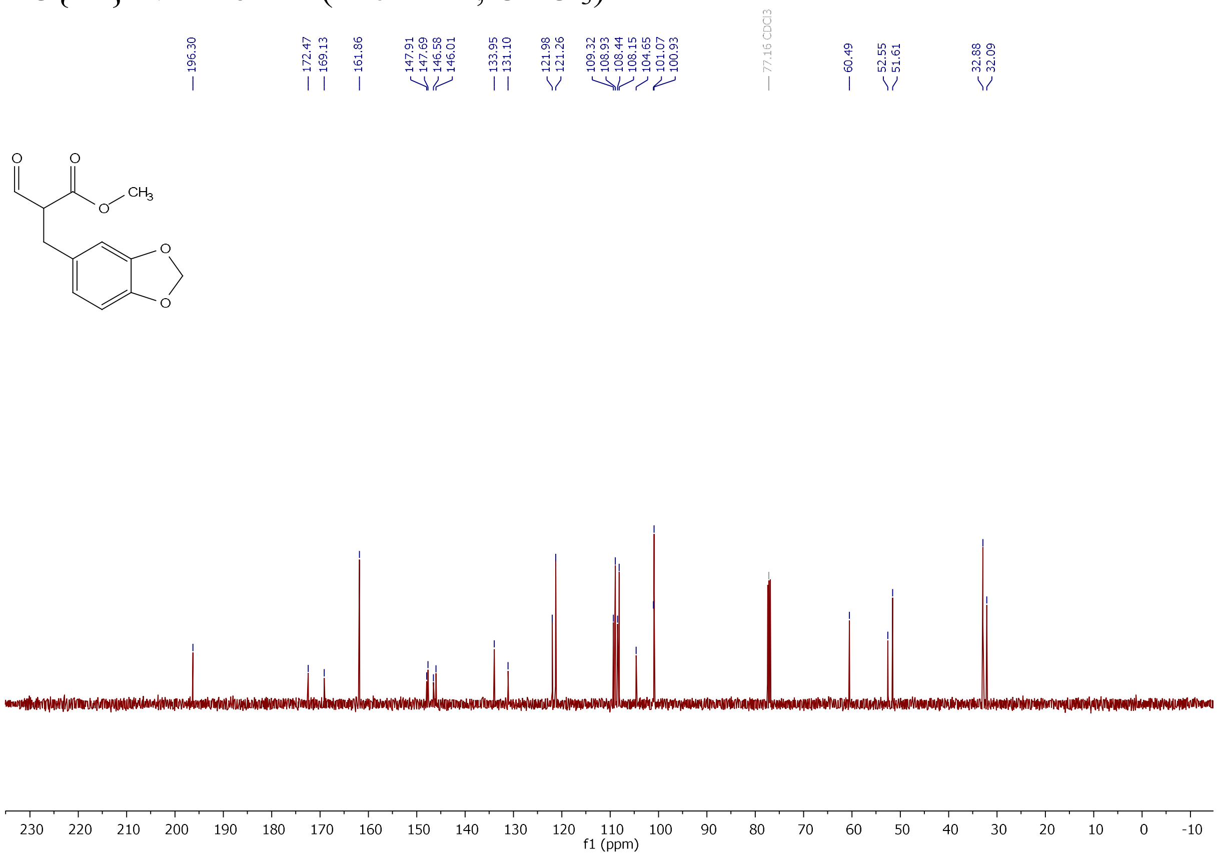

^{1}H NMR of **2l** (500 MHz, DMSO-d_6)

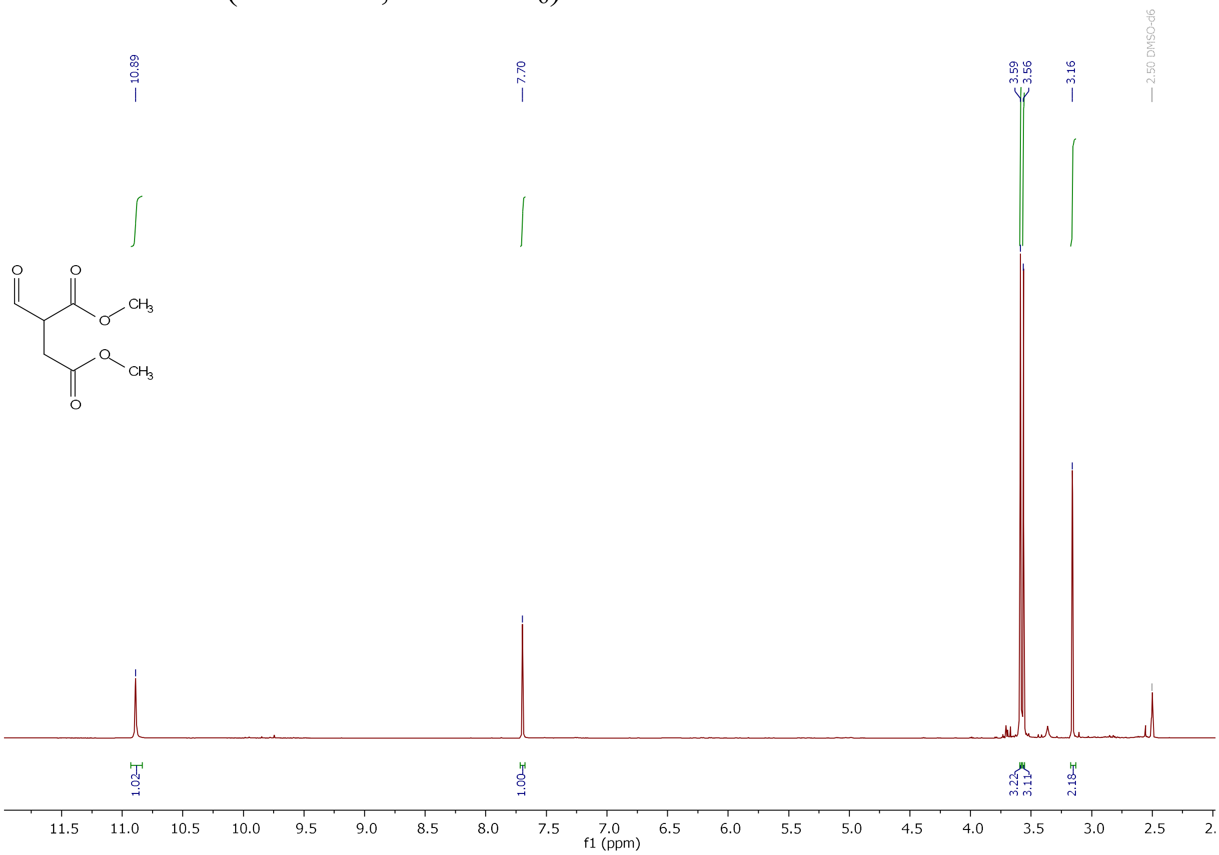

$^{13}C\{^{1}H\}$ NMR of **2l** (126 MHz, DMSO-d_6)

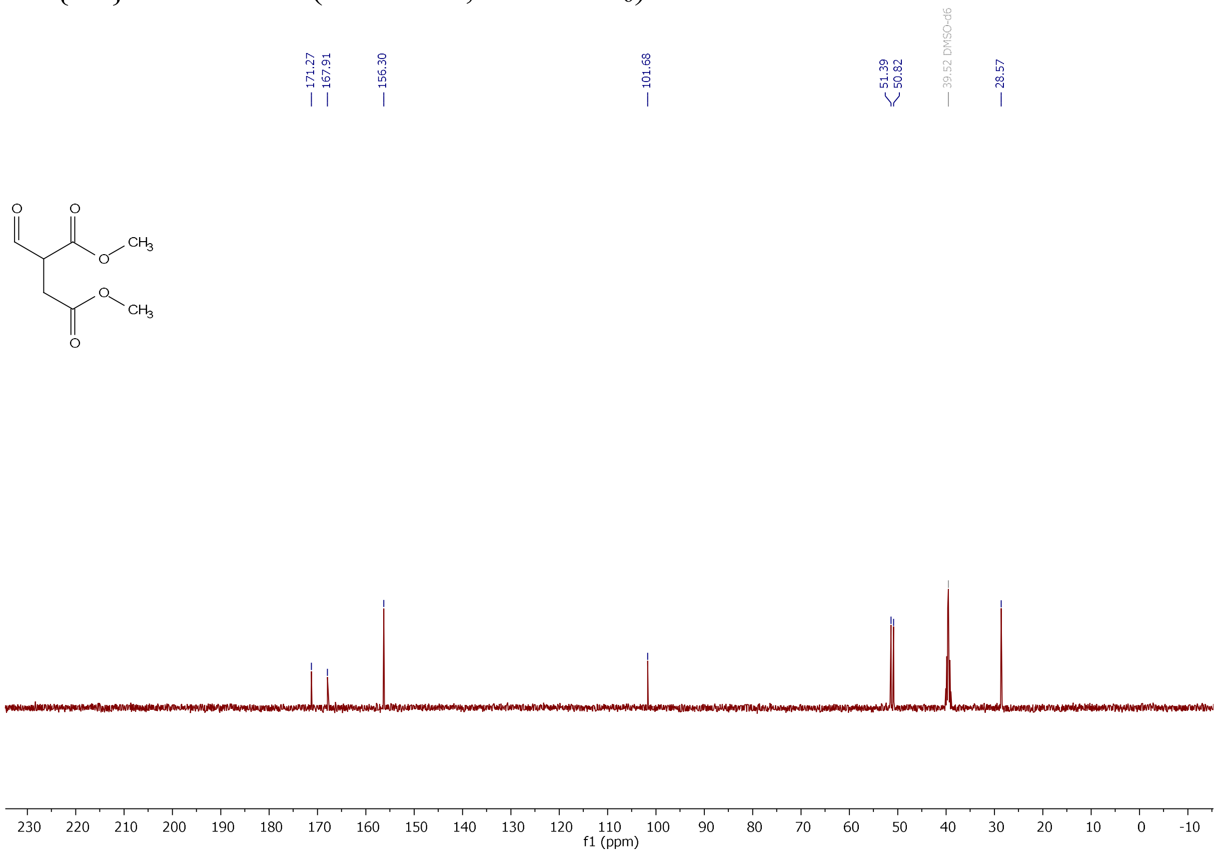

^{1}H NMR of **3a’** (500 MHz, $CDCl_3$)

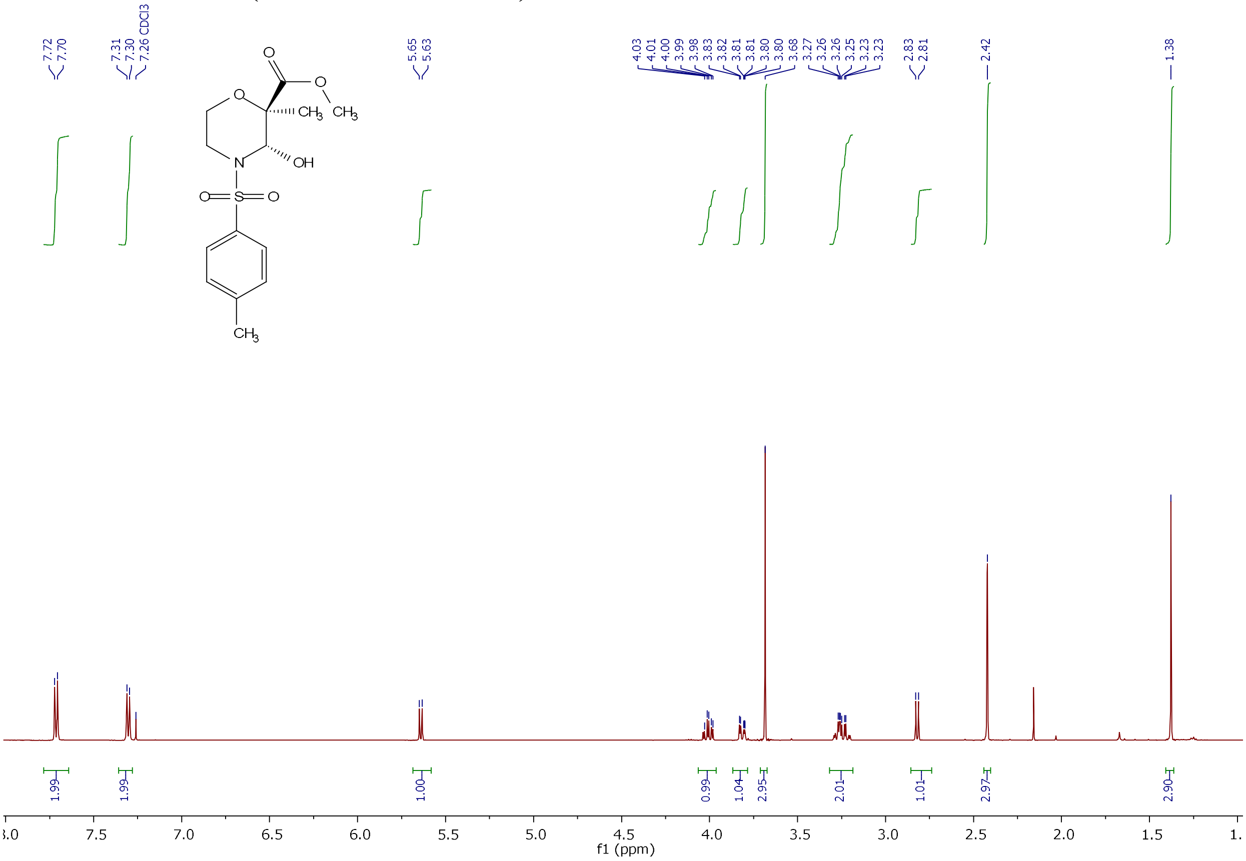

^{13}C{^{1}H} NMR of **3a’** (126 MHz, $CDCl_3$)

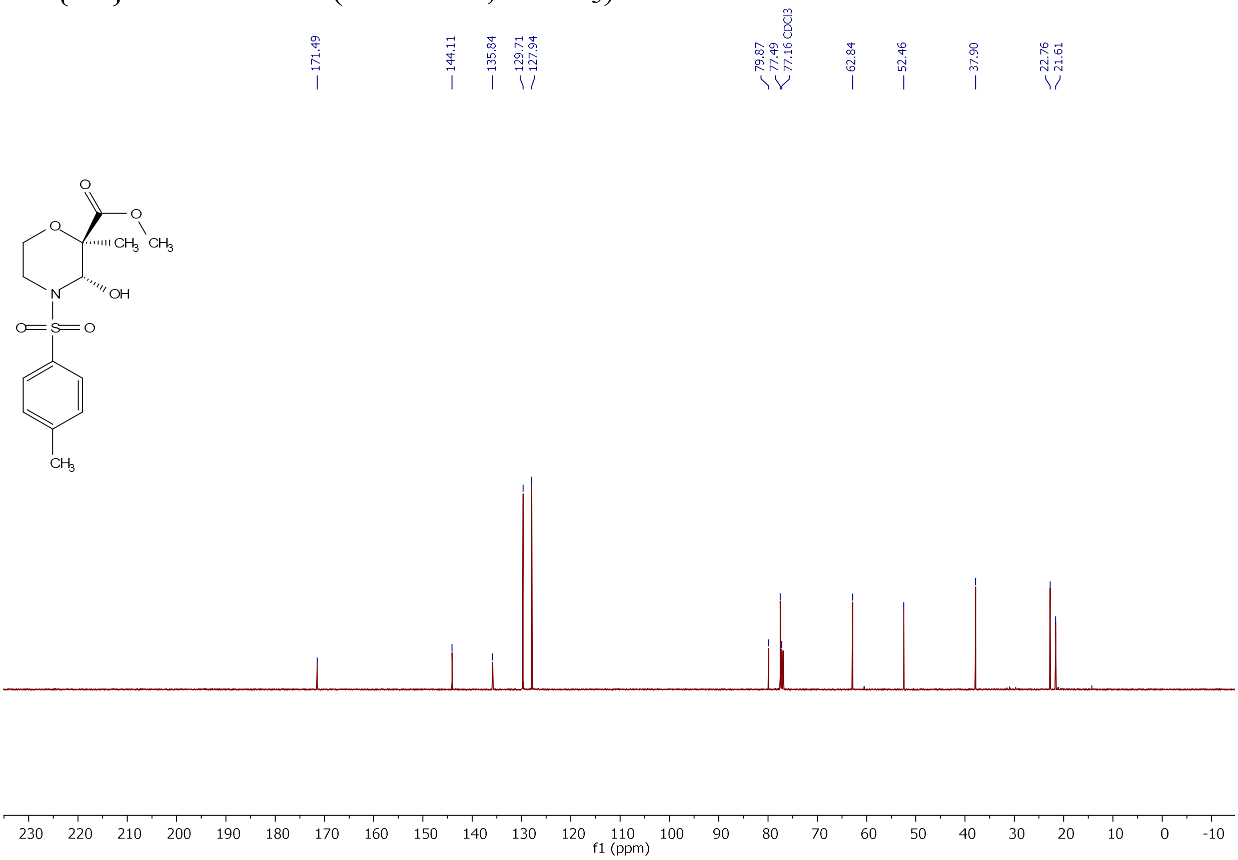

Key NOESY correlations of compound **3a'**

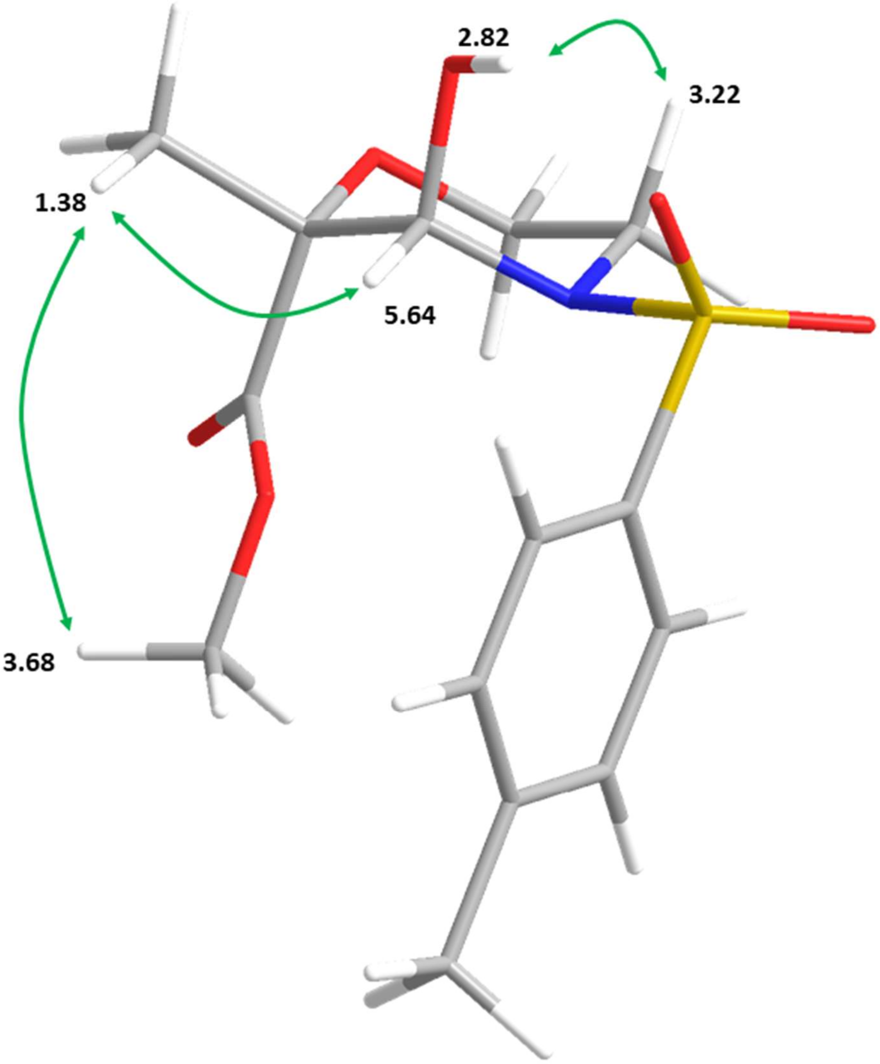

^{1}H NMR of **3a''** (500 MHz, $CDCl_3$)

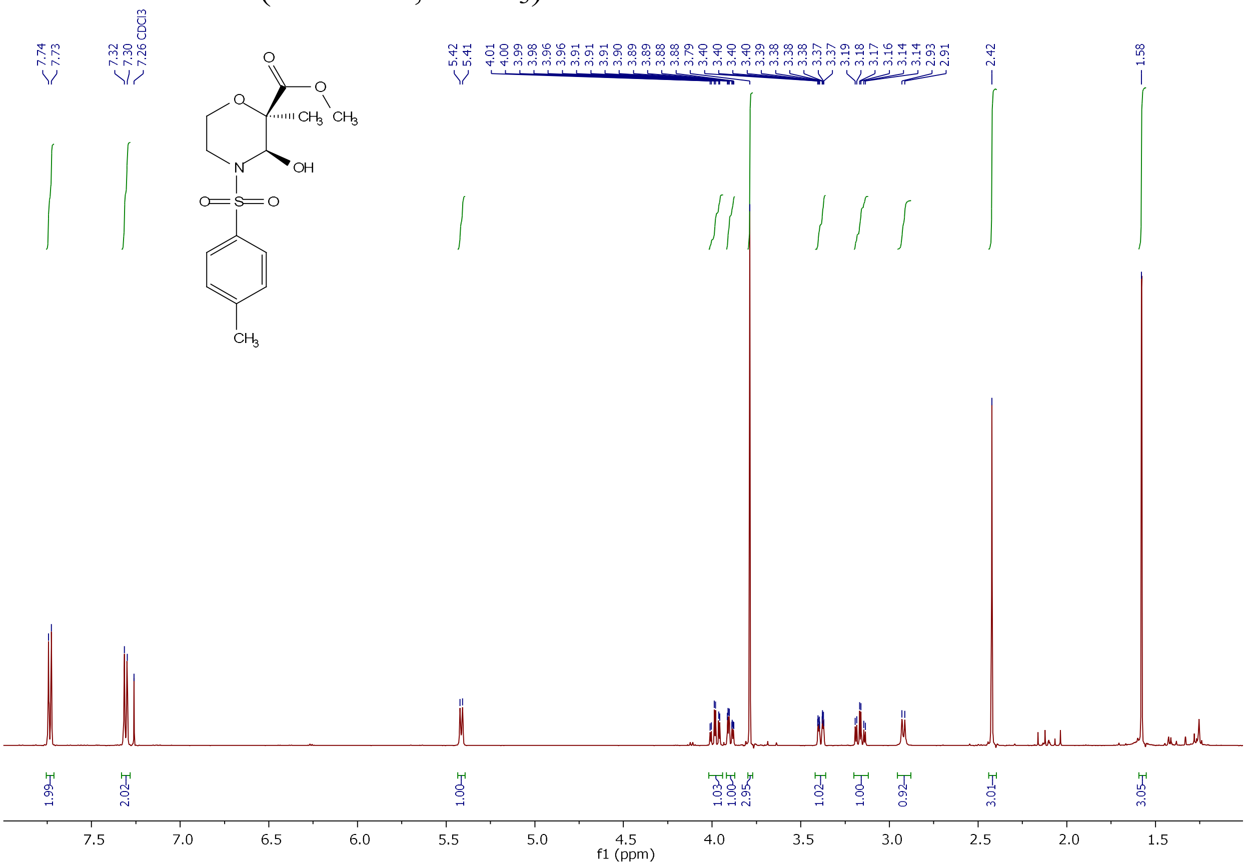

$^{13}C\{^1H\}$ NMR of **3a''** (126 MHz, $CDCl_3$)

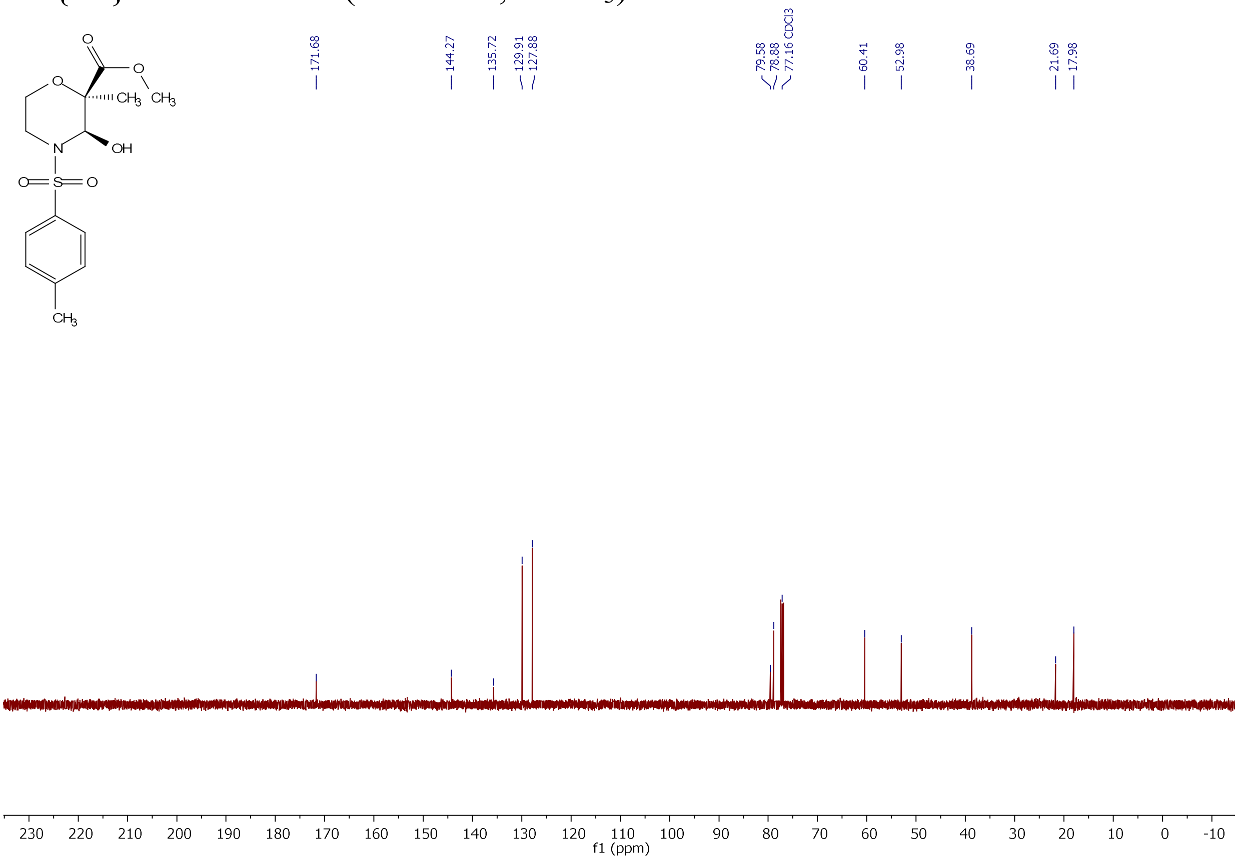

Key NOESY correlations of compound **3a''**

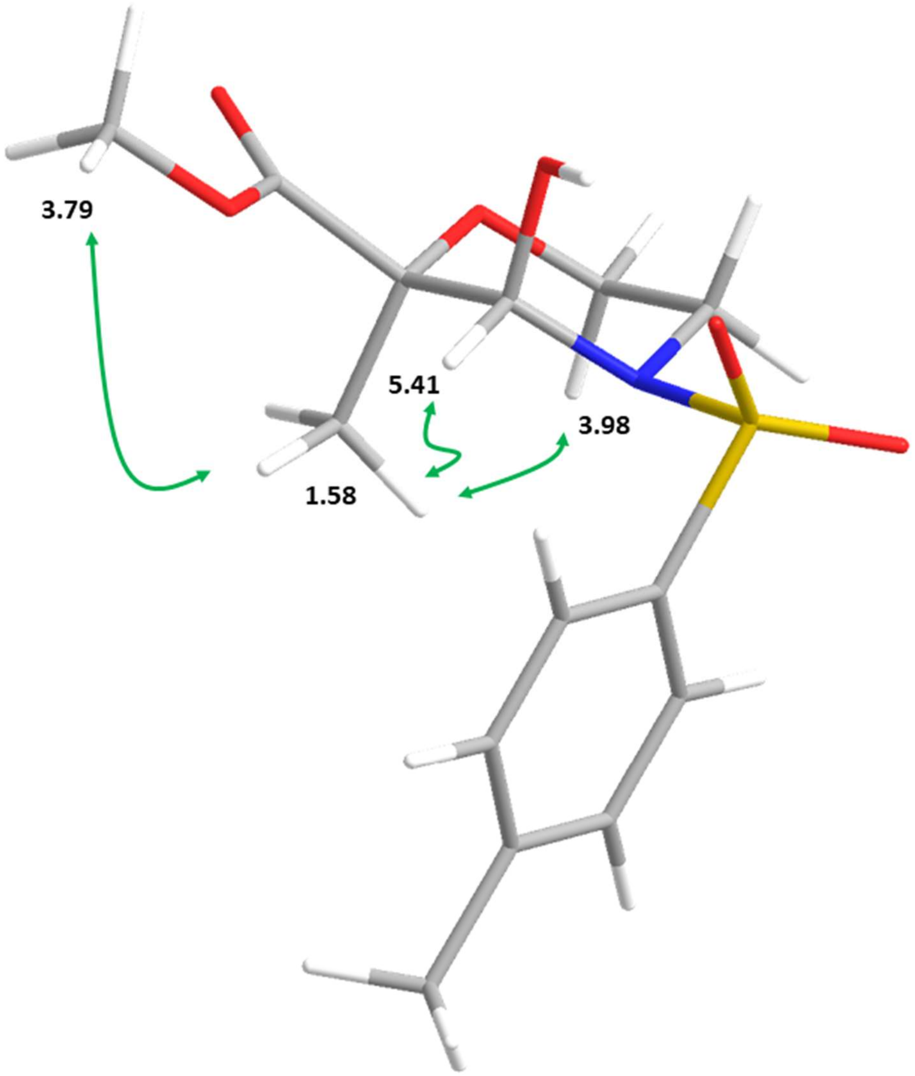

^{1}H NMR of **3b** (500 MHz, $CDCl_3$)

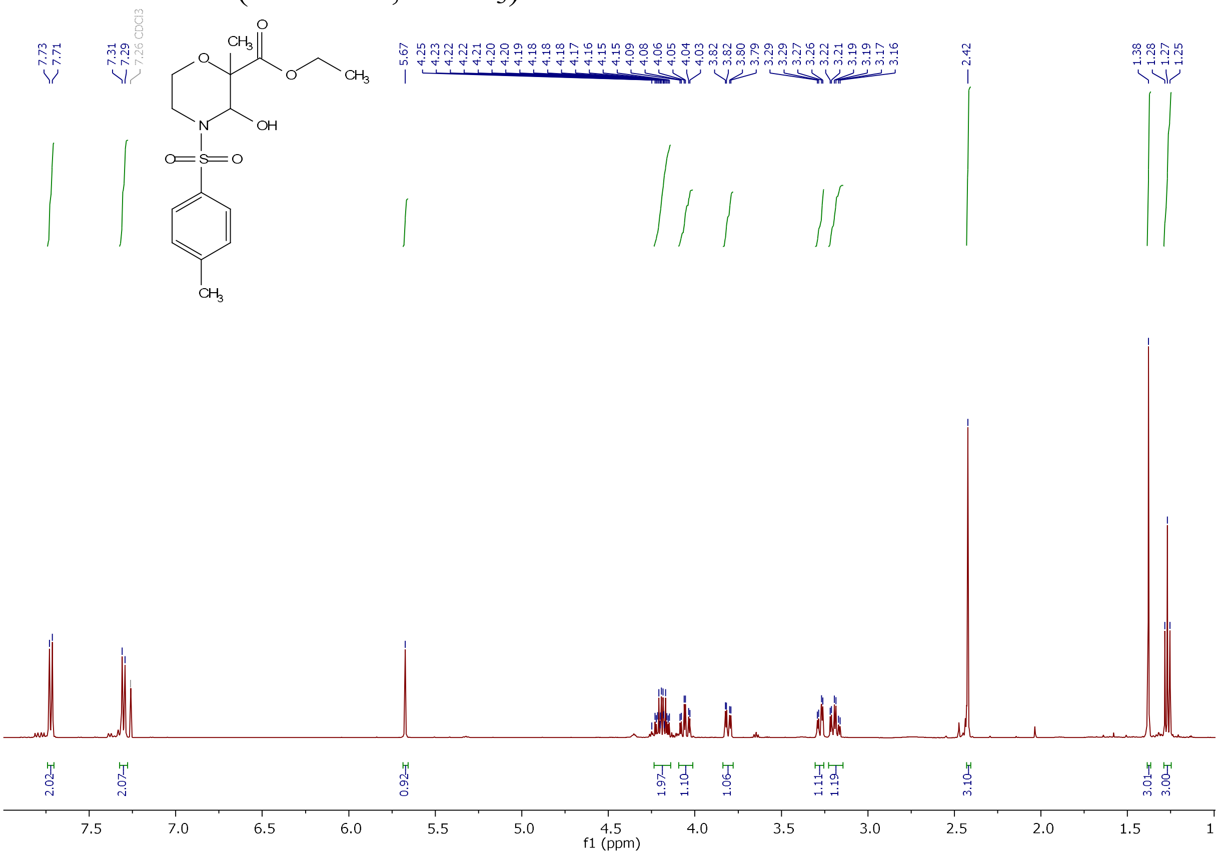

$^{13}C\{^{1}H\}$ NMR of **3b** (126 MHz, $CDCl_3$)

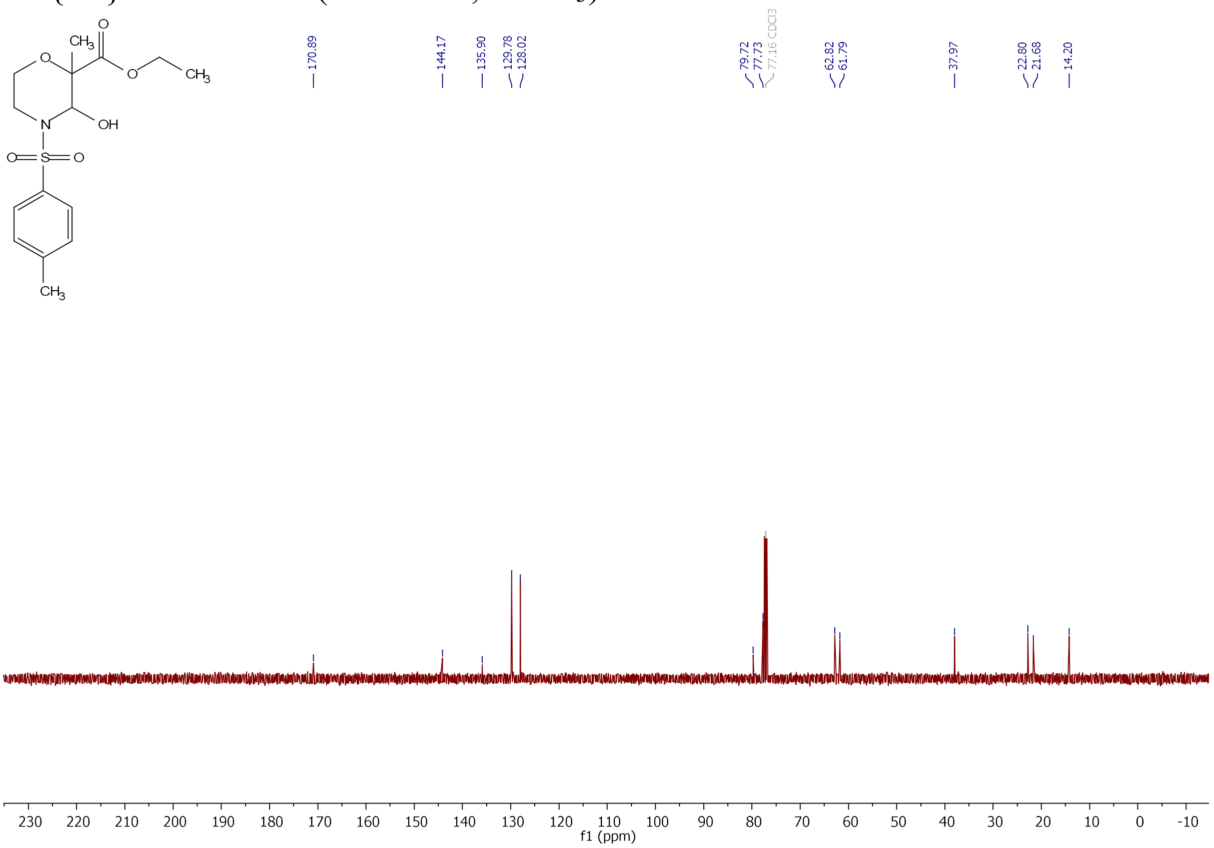

^{1}H NMR of **3c** (500 MHz, $CDCl_3$)

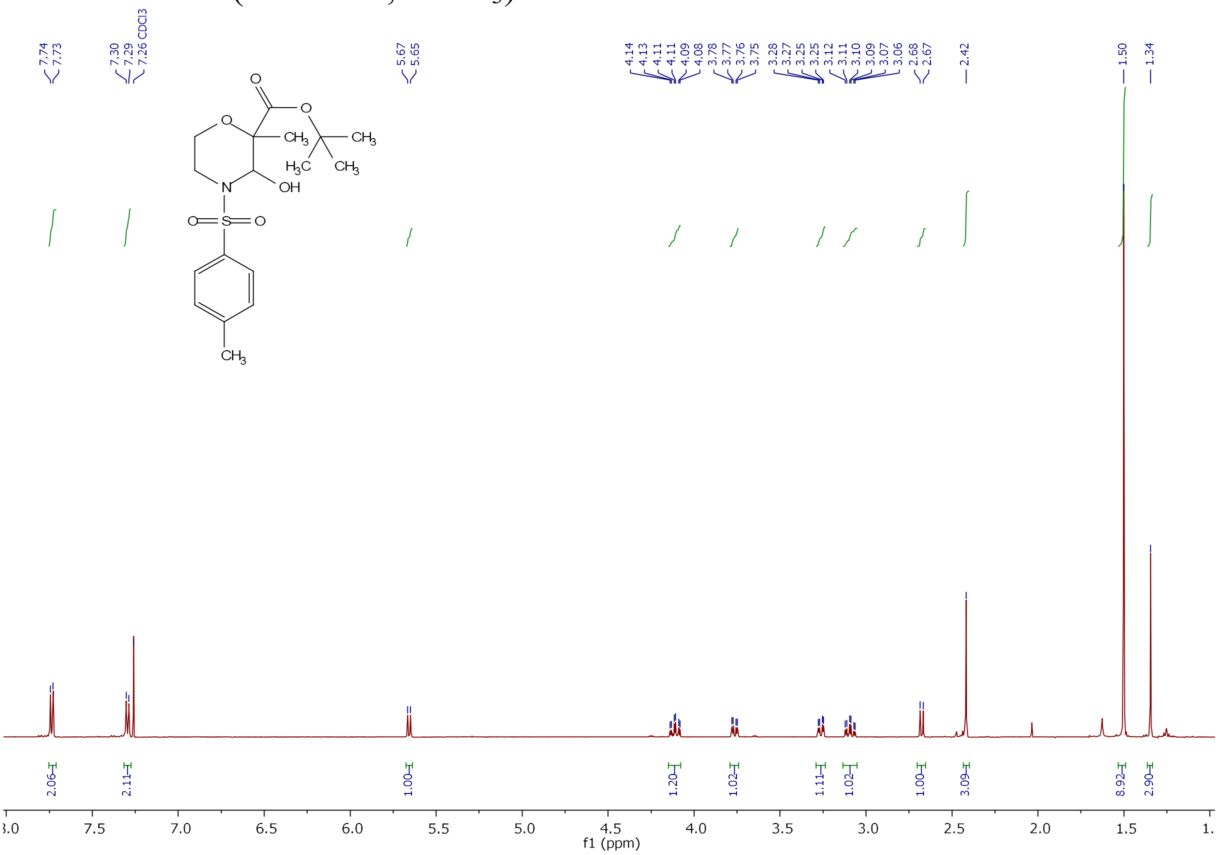

^{13}C{^{1}H} NMR of **3c** (126 MHz, $CDCl_3$)

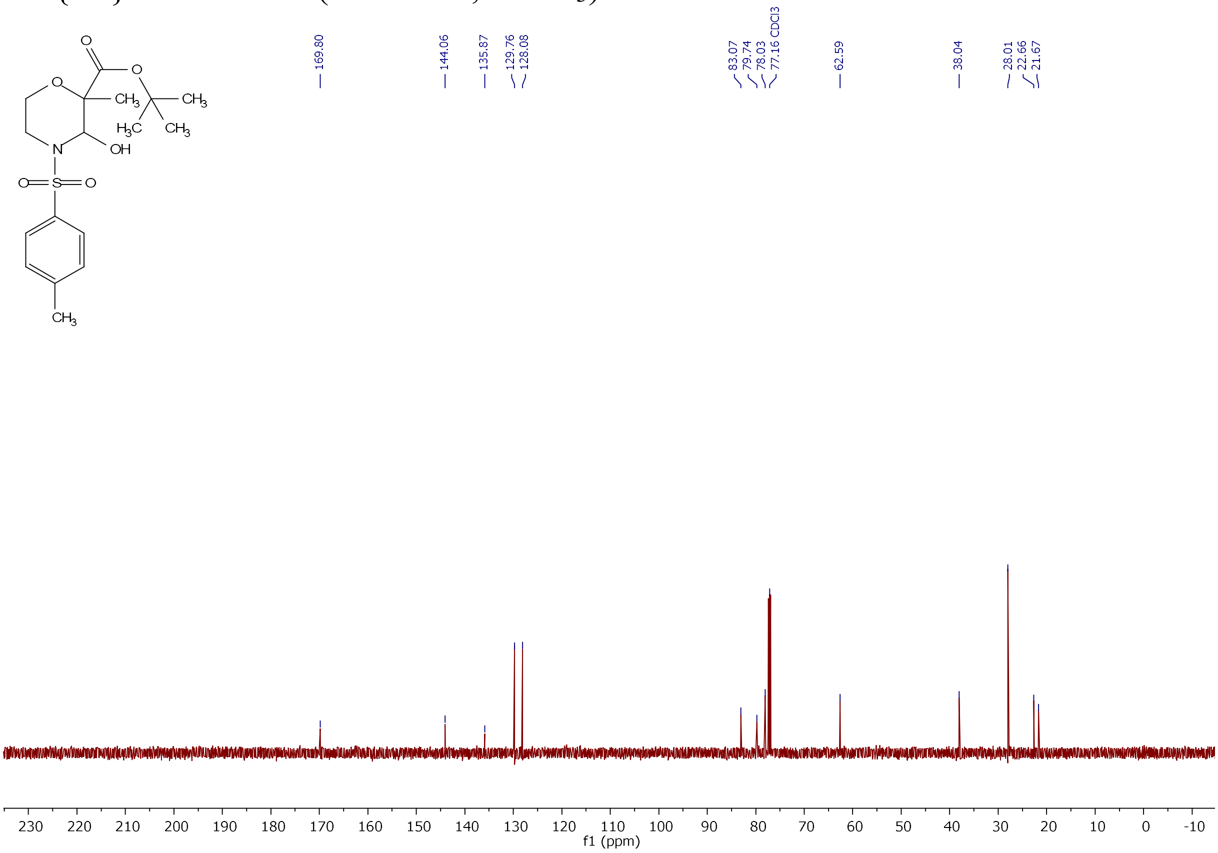

^{1}H NMR of **3d** (500 MHz, $CDCl_3$)

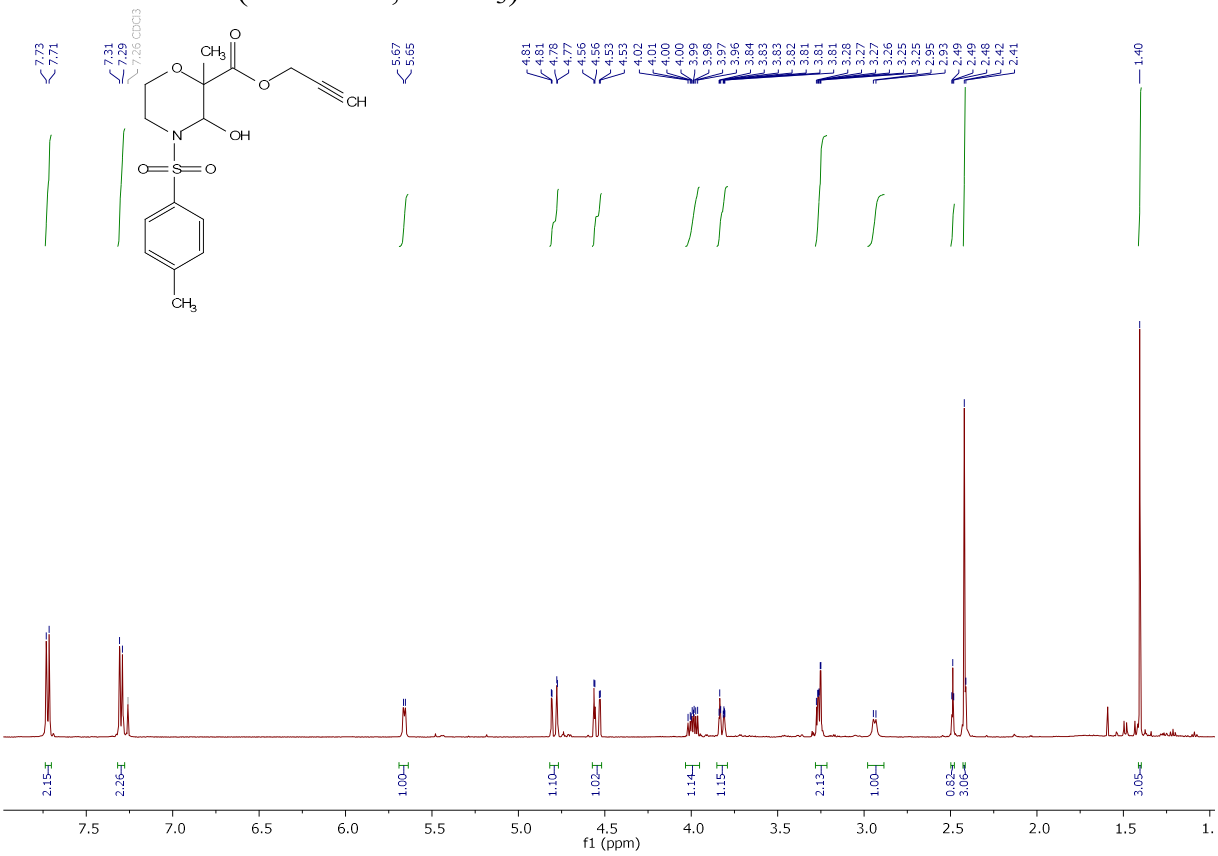

$^{13}C\{^{1}H\}$ NMR of **3d** (126 MHz, $CDCl_3$)

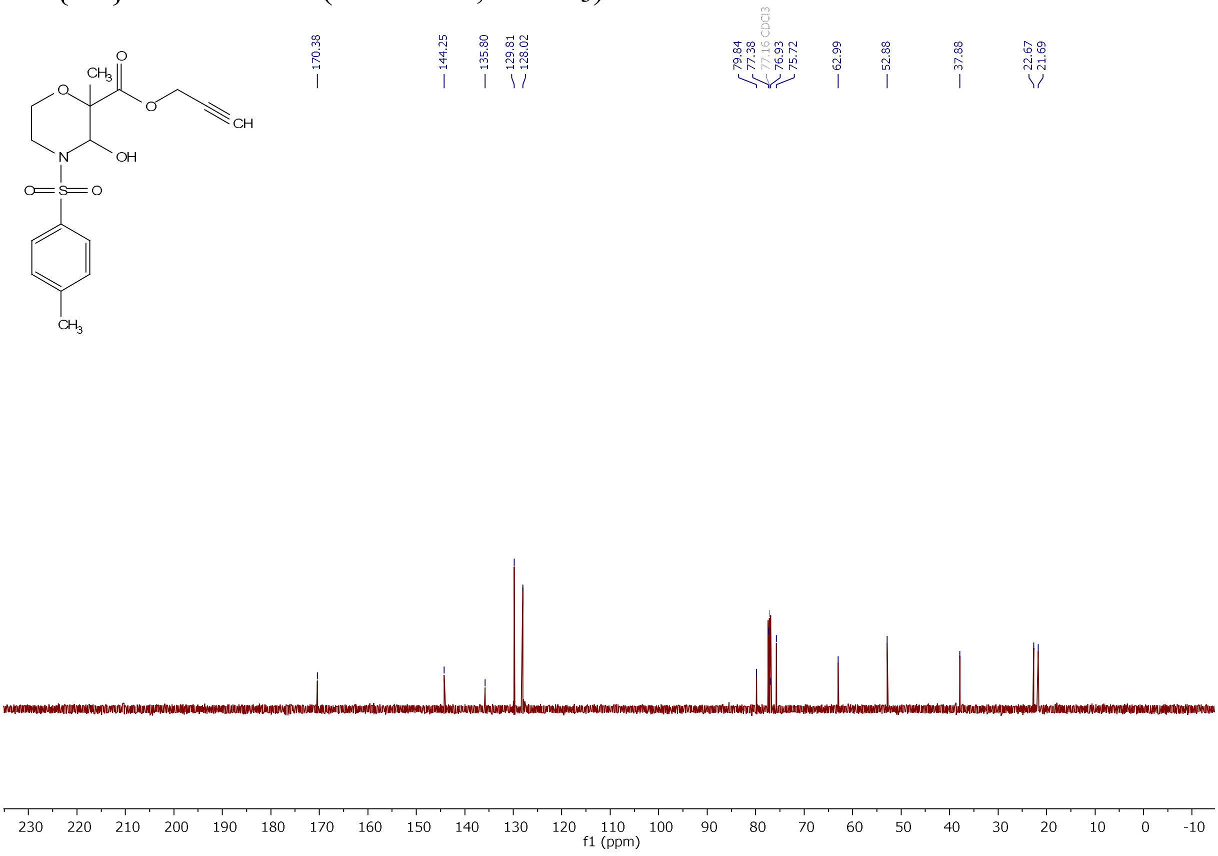

^{1}H NMR of **3e'** (500 MHz, $CDCl_3$)

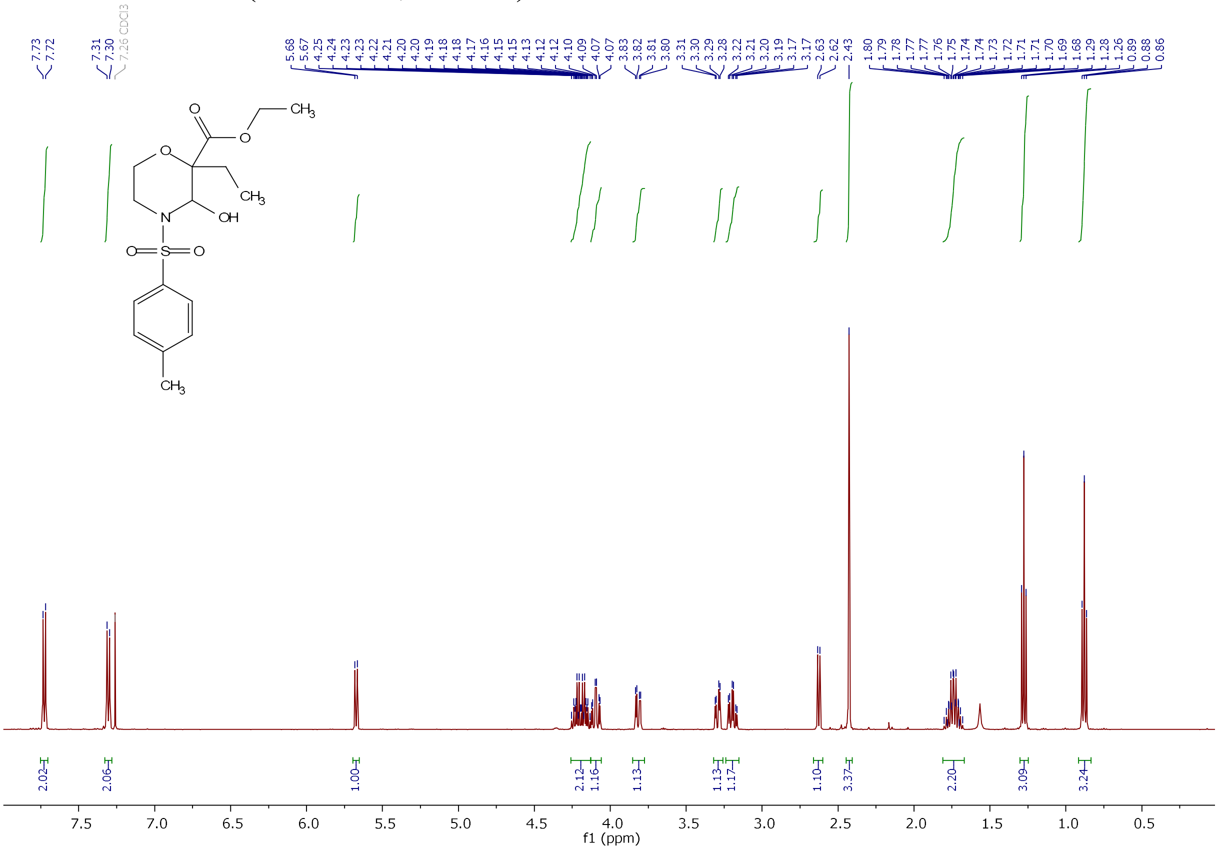

^{13}C{^{1}H} NMR of **3e'** (126 MHz, $CDCl_3$)

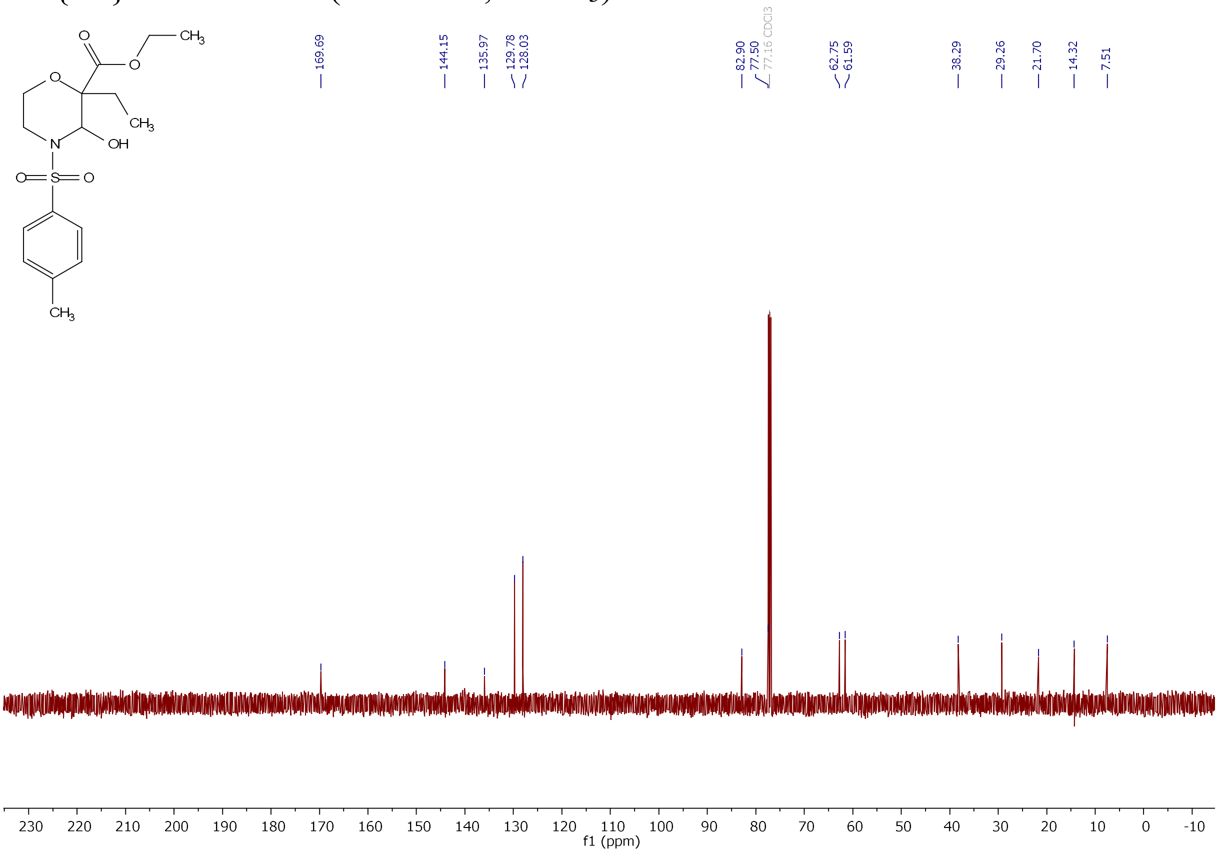

^{1}H NMR of **3e''** (500 MHz, $CDCl_3$)

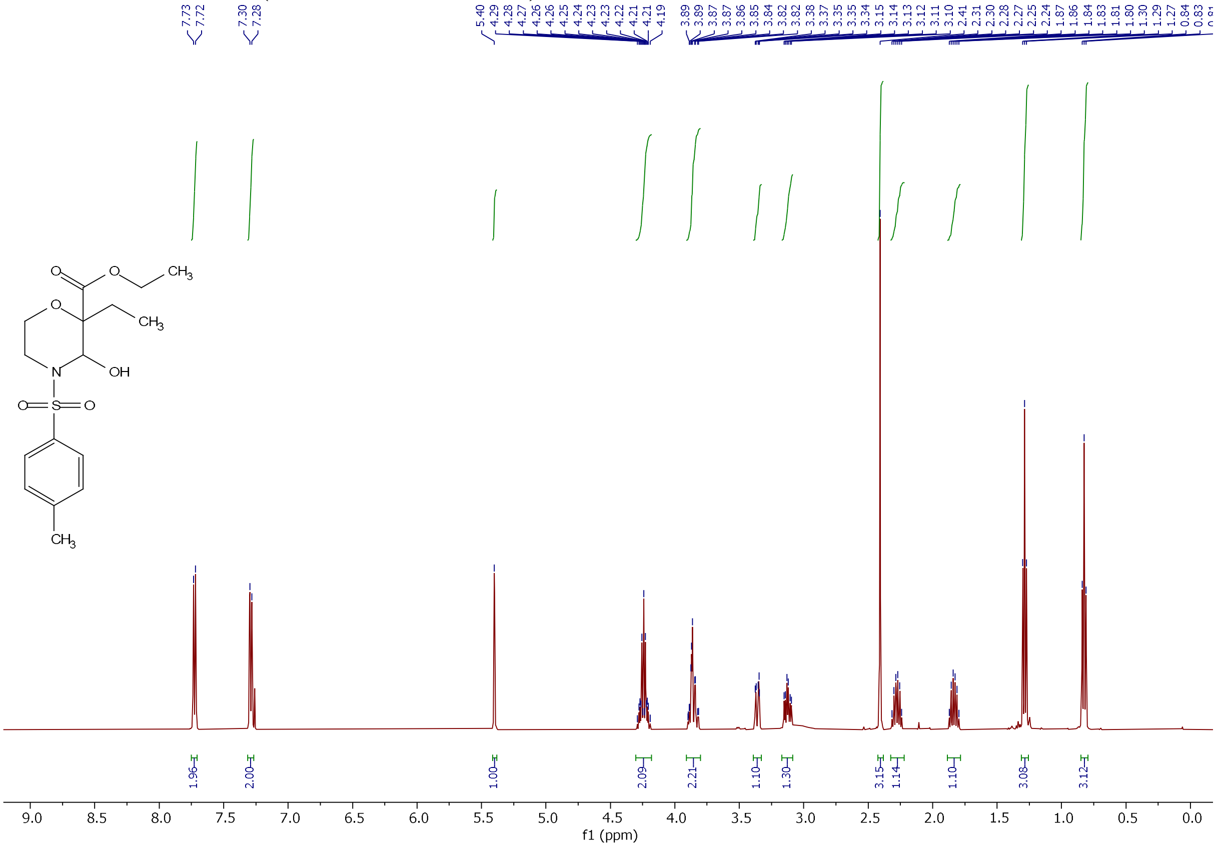

^{13}C{^{1}H} NMR of **3e''** (126 MHz, $CDCl_3$)

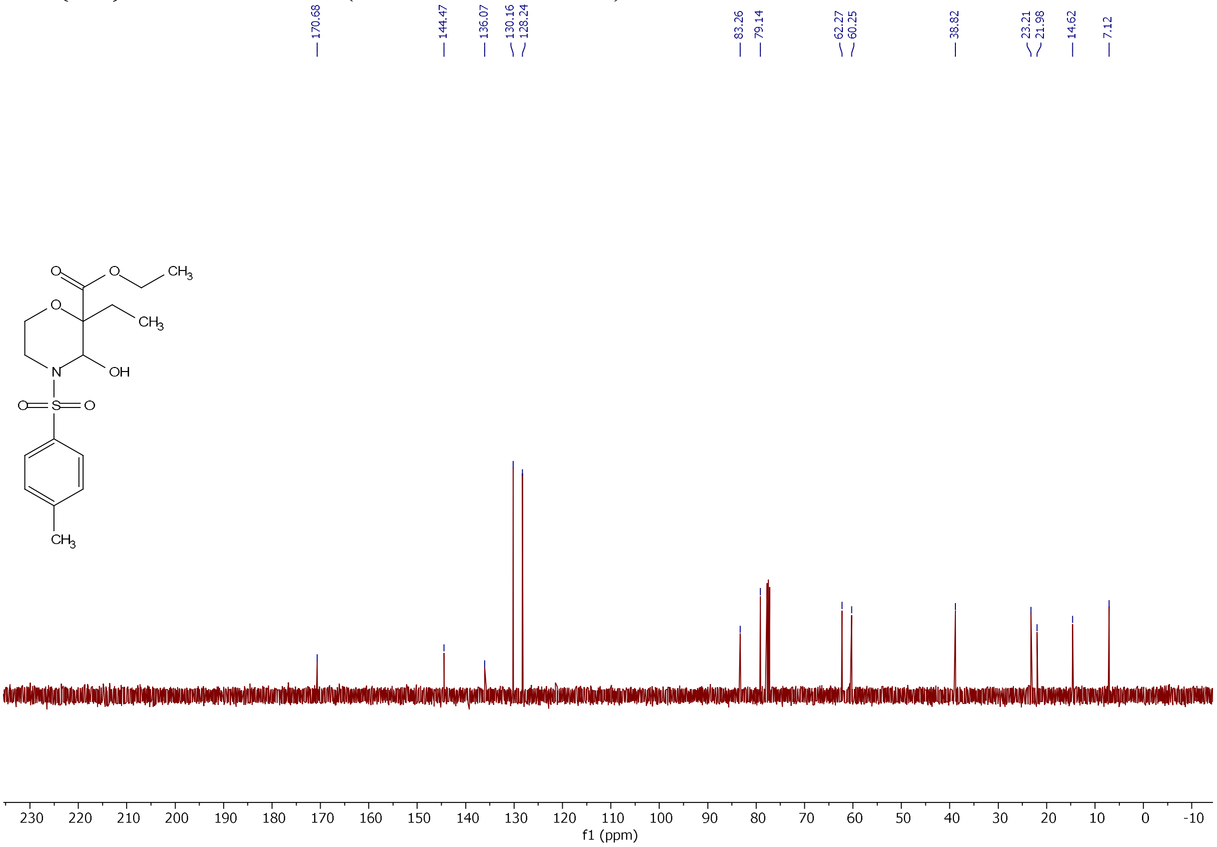

^{1}H NMR of **3f** (500 MHz, $CDCl_3$)

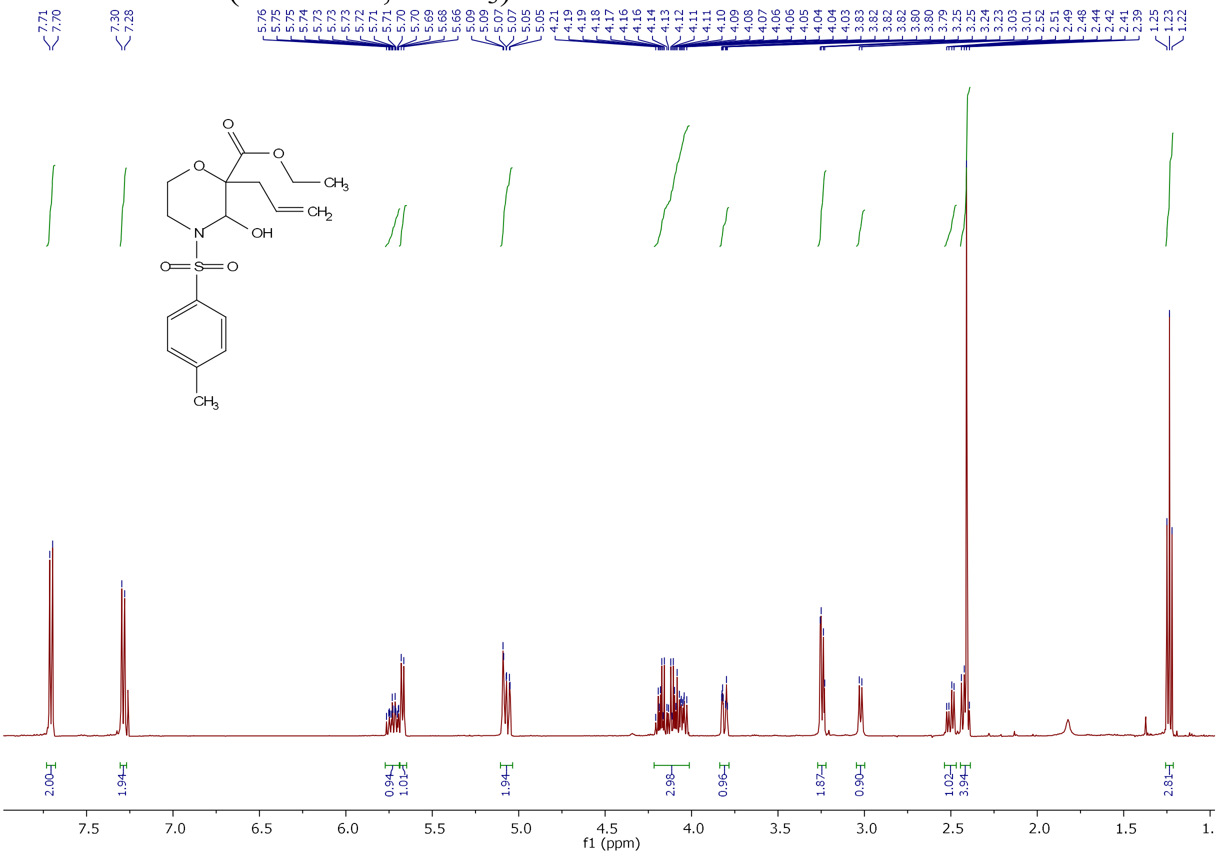

^{13}C{^{1}H} NMR of **3f** (126 MHz, $CDCl_3$)

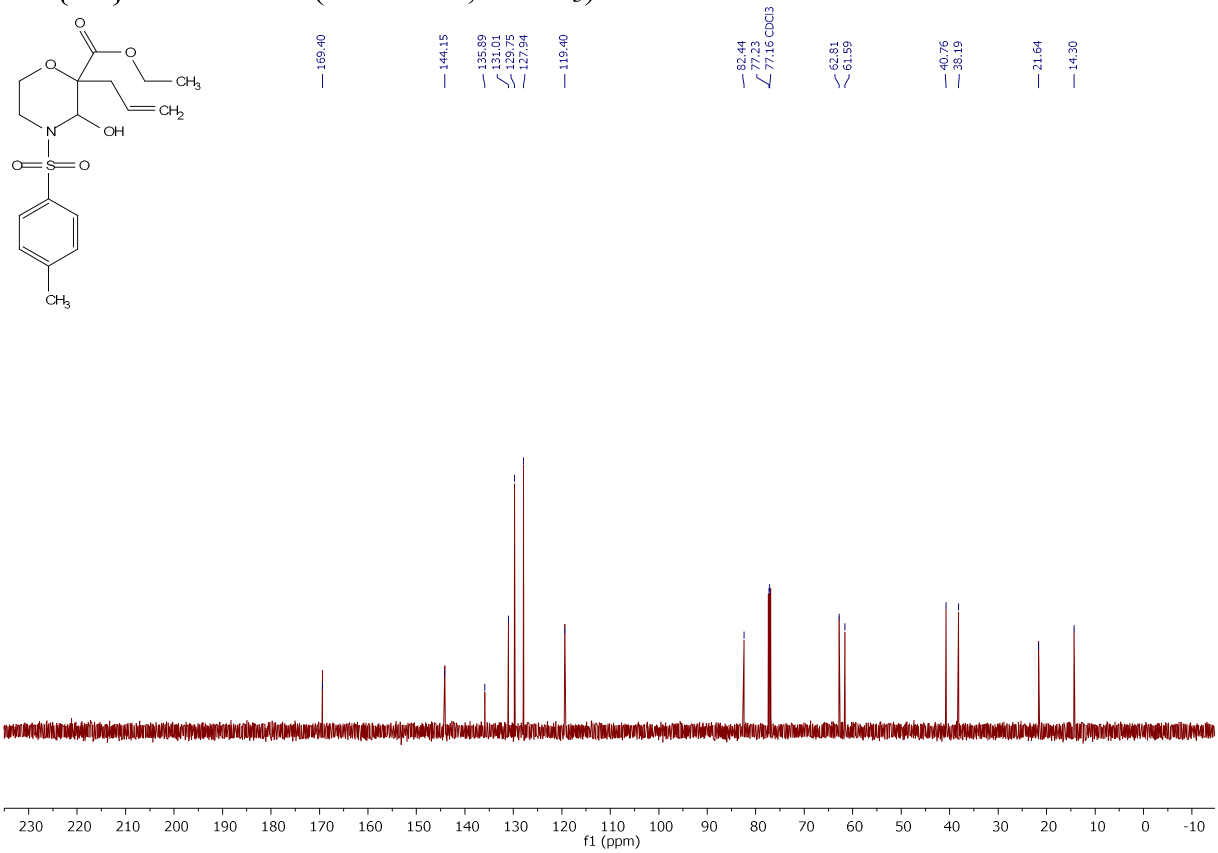

^{1}H NMR of **3g** (500 MHz, $CDCl_3$)

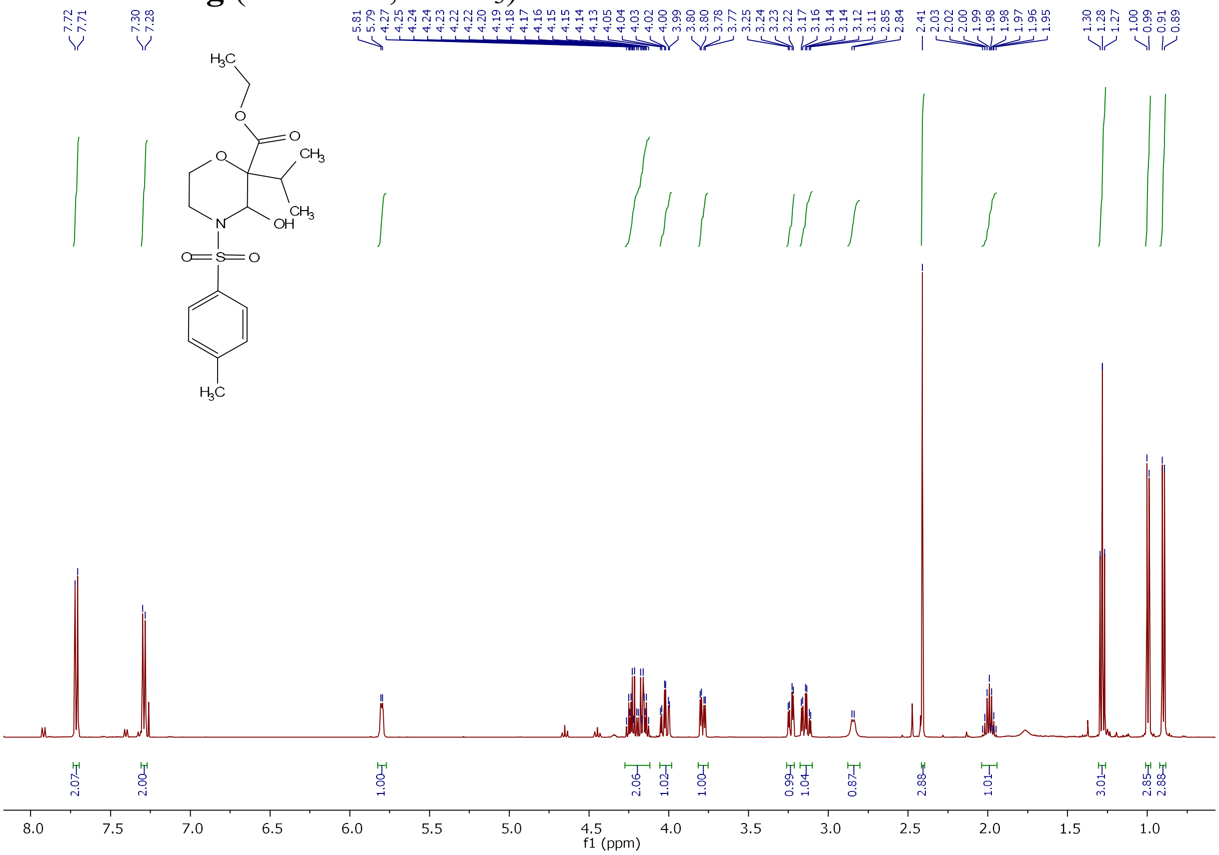

$^{13}C\{^{1}H\}$ NMR of **3g** (126 MHz, $CDCl_3$)

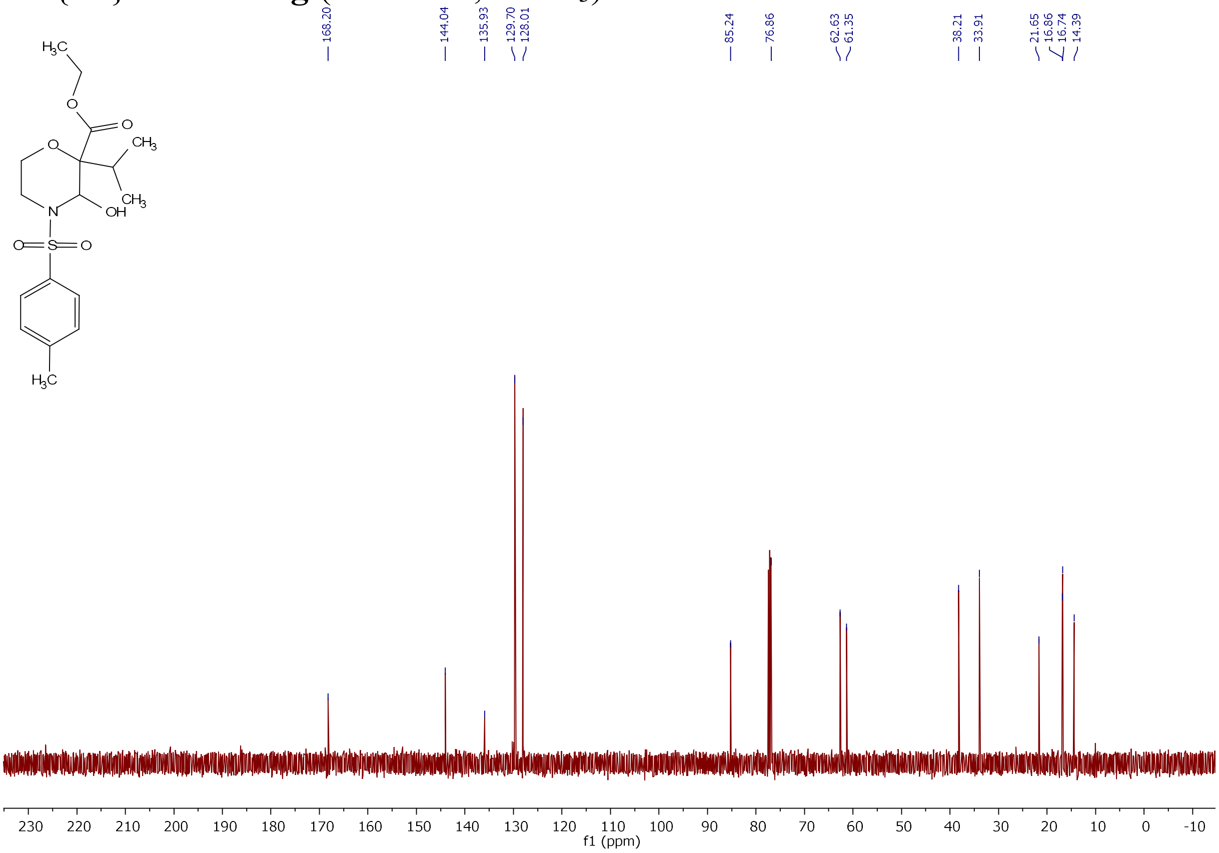

^{1}H NMR of **3h** (500 MHz, $CDCl_3$)

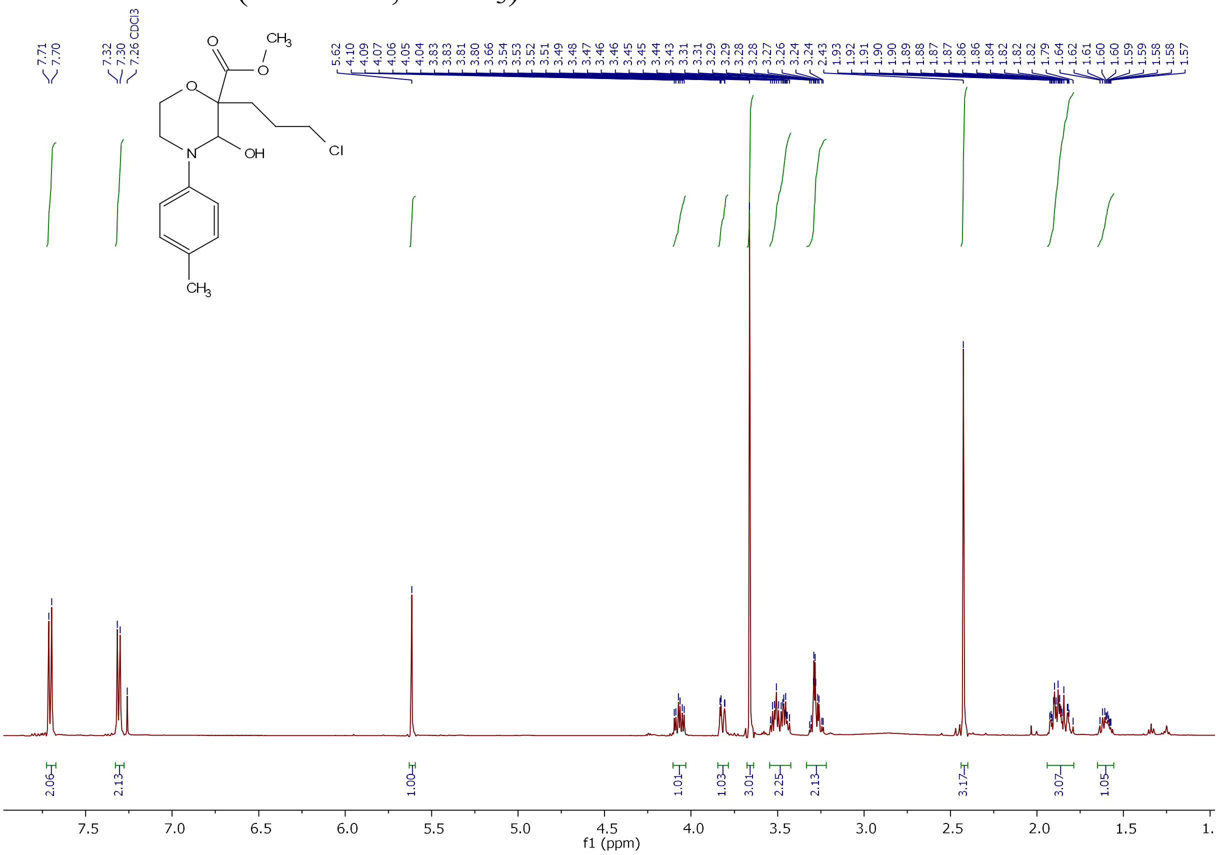

^{13}C{^{1}H} NMR of **3h** (126 MHz, $CDCl_3$)

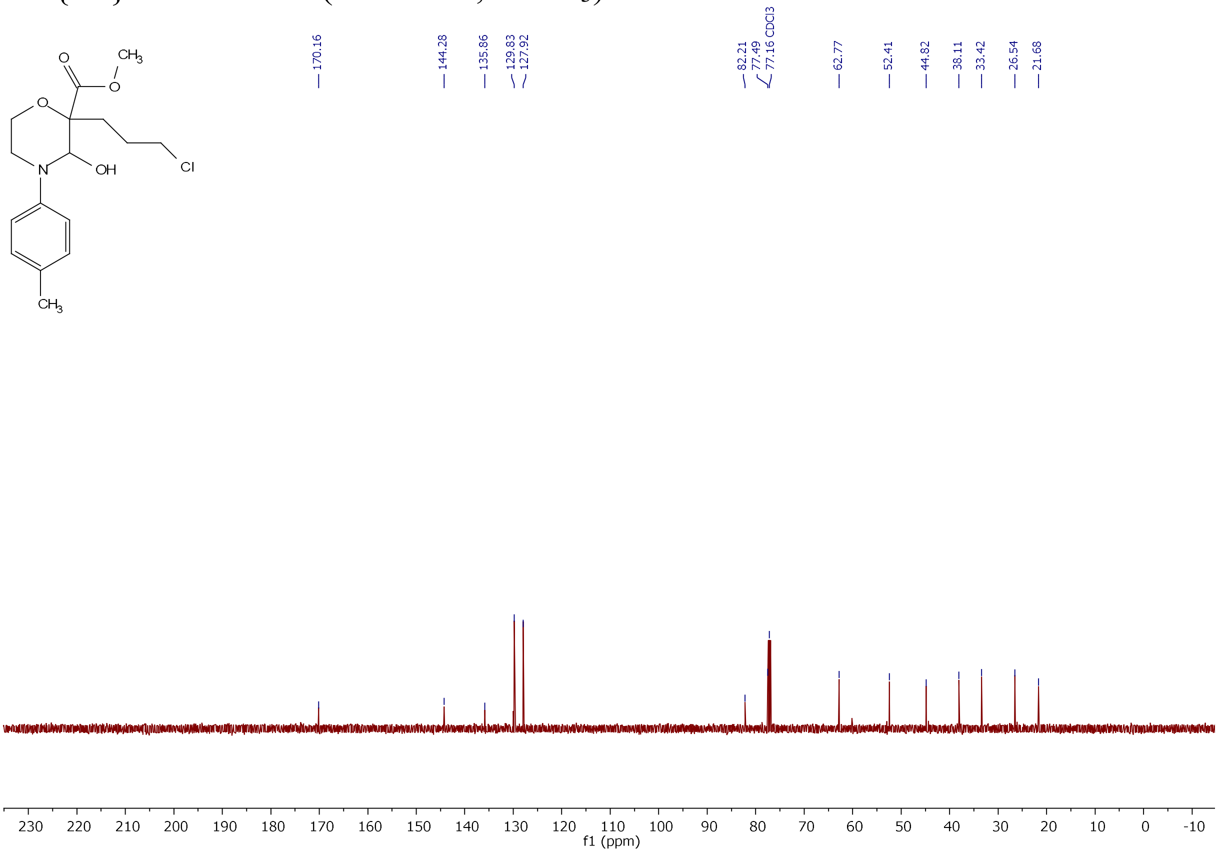

^{1}H NMR of **3i’** (500 MHz, $CDCl_3$)

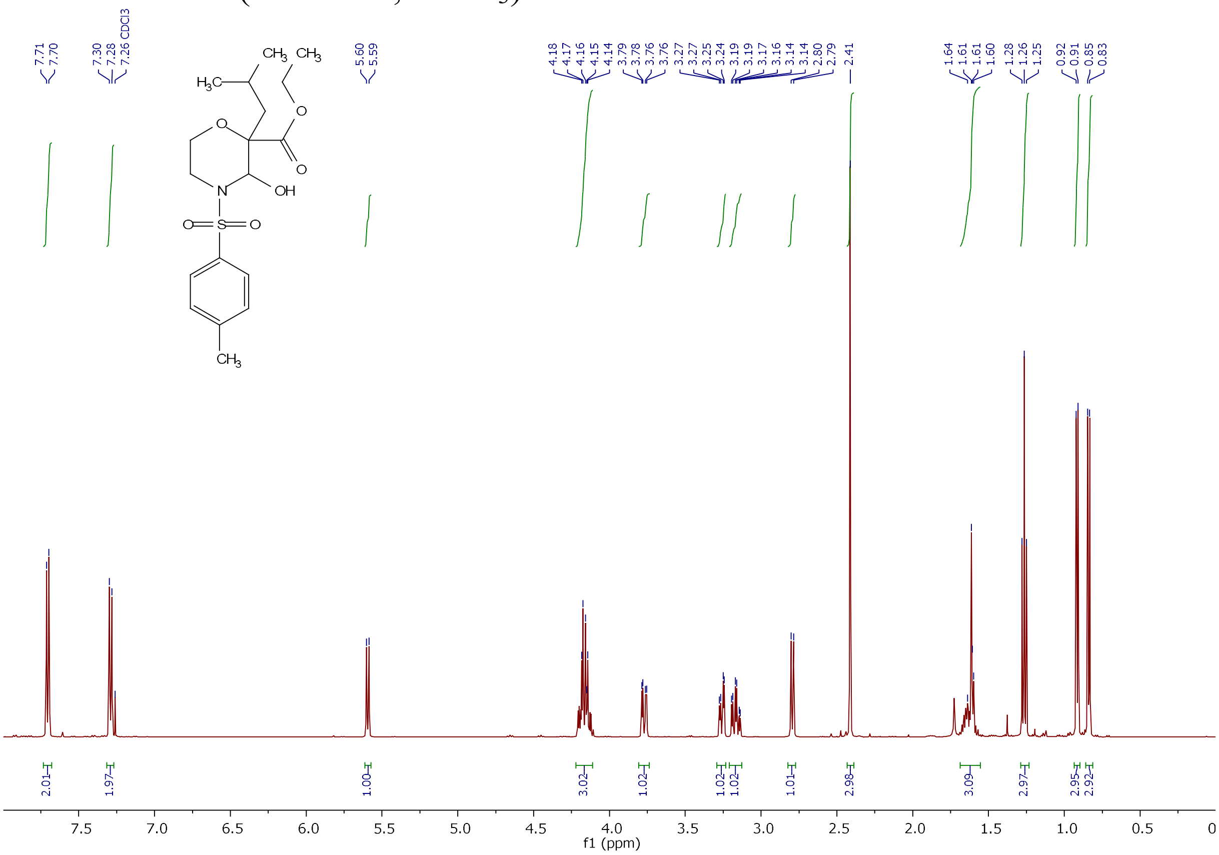

^{13}C{^{1}H} NMR of **3i’** (126 MHz, $CDCl_3$)

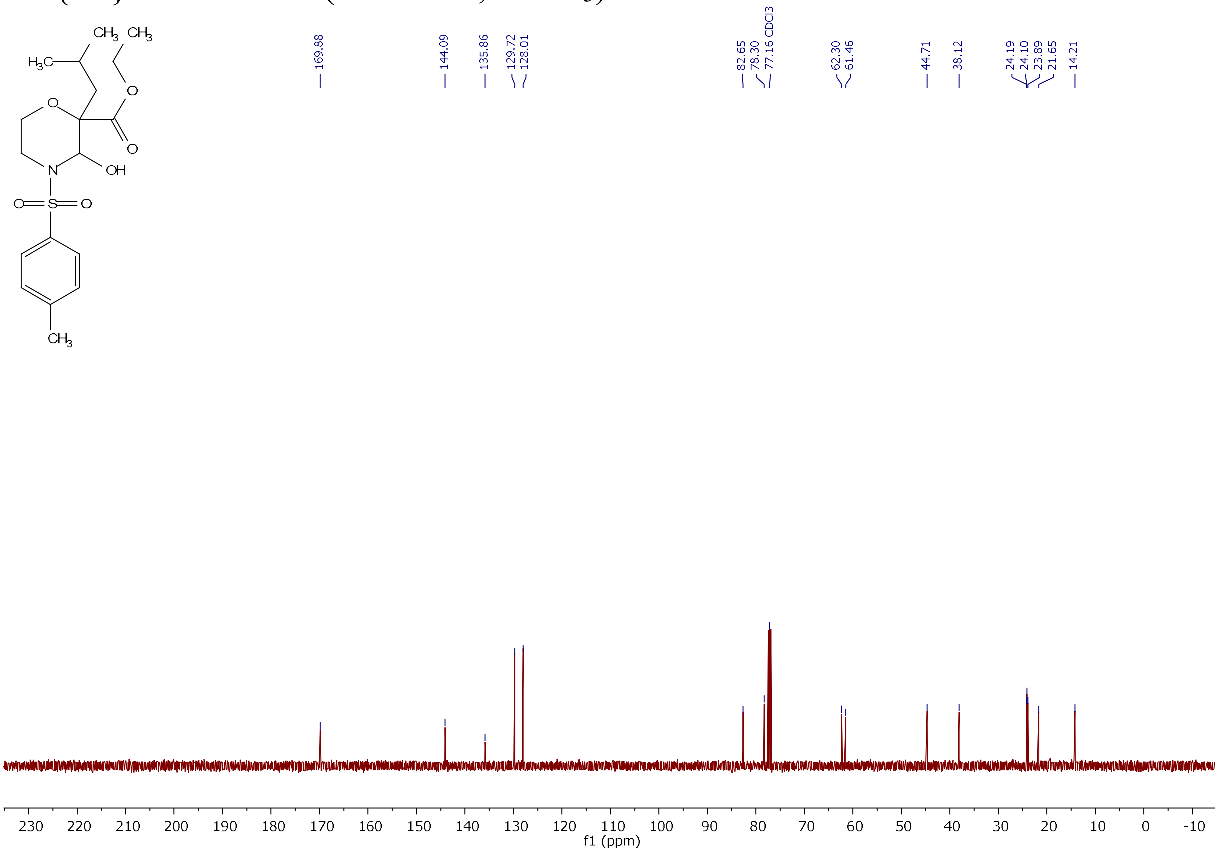

^{1}H NMR of **3i''** (500 MHz, $CDCl_3$)

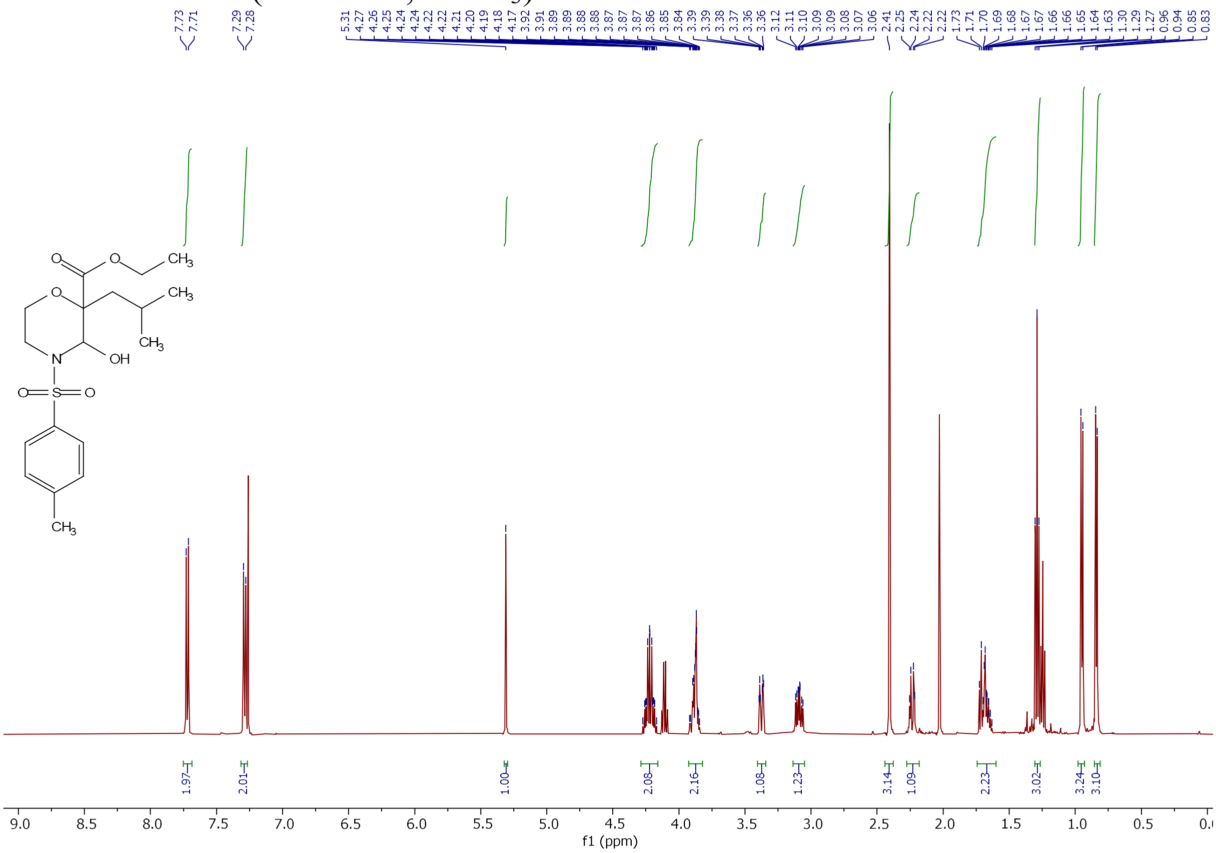

^{13}C{^{1}H} NMR of **3i''** (126 MHz, $CDCl_3$)

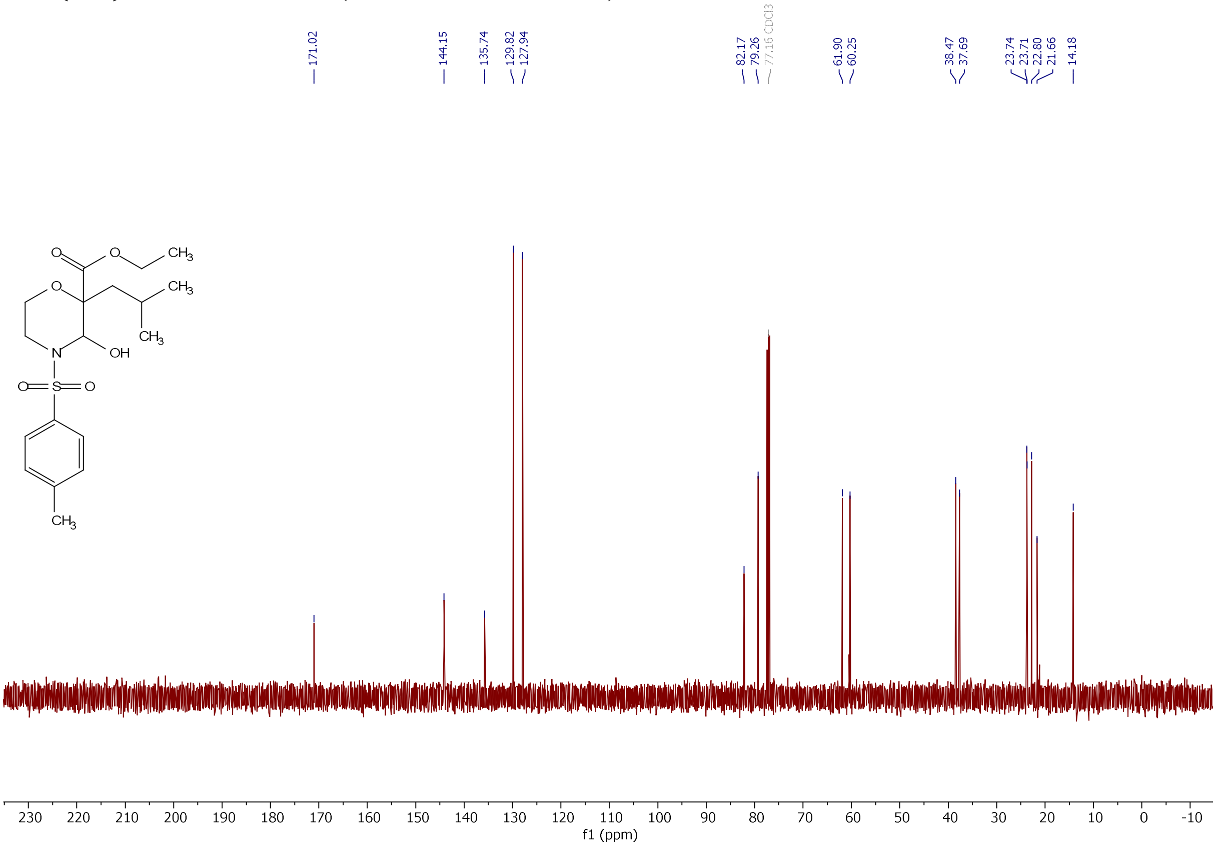

^{1}H NMR of **3j'** (500 MHz, $CDCl_3$)

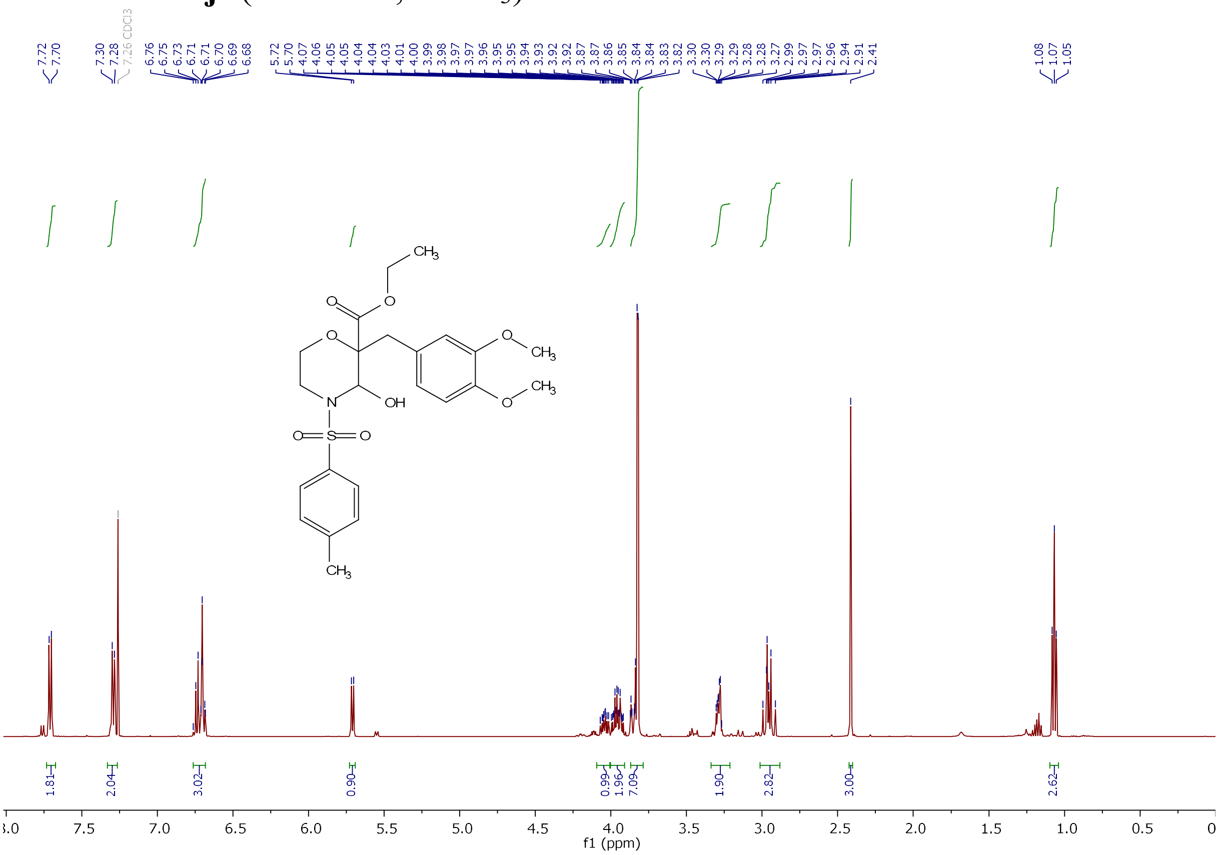

^{13}C{^{1}H} NMR of **3j'** (126 MHz, $CDCl_3$)

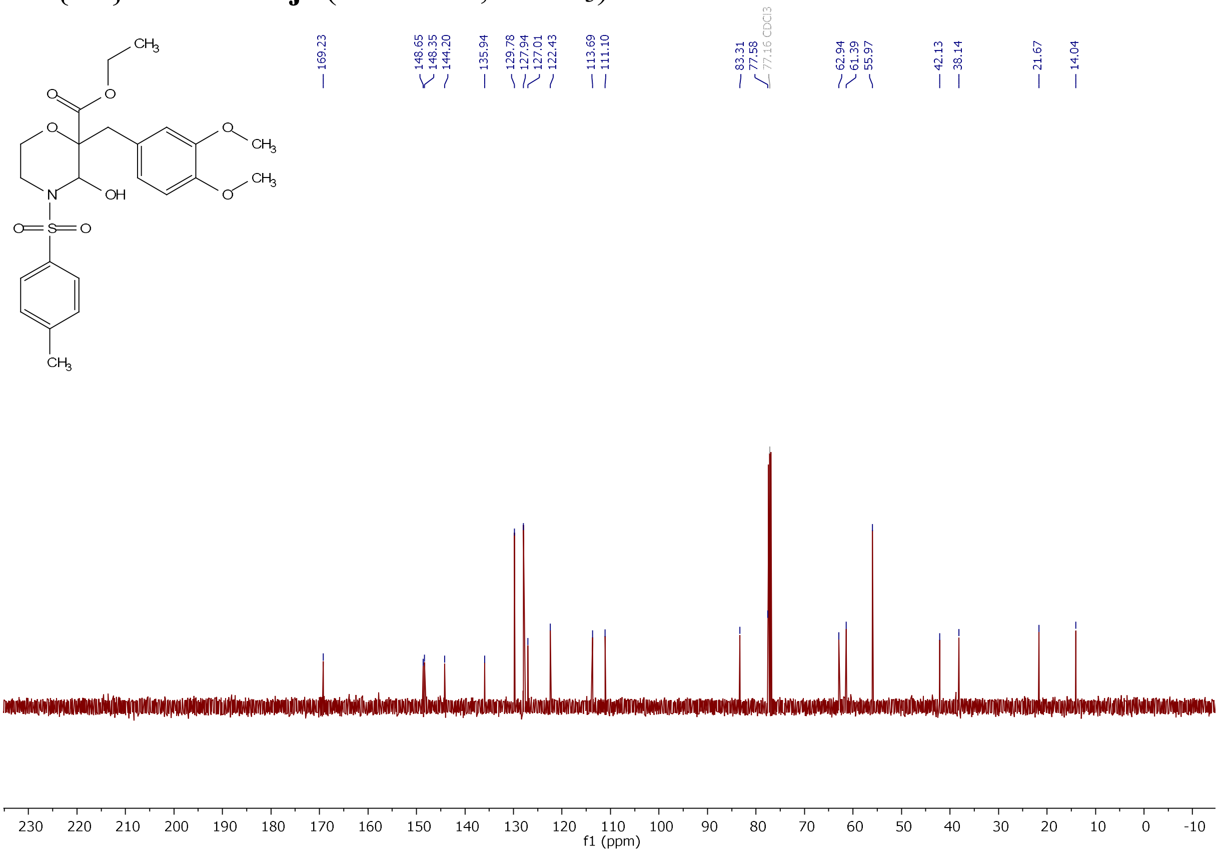

^{1}H NMR of **3j''** (500 MHz, $CDCl_3$)

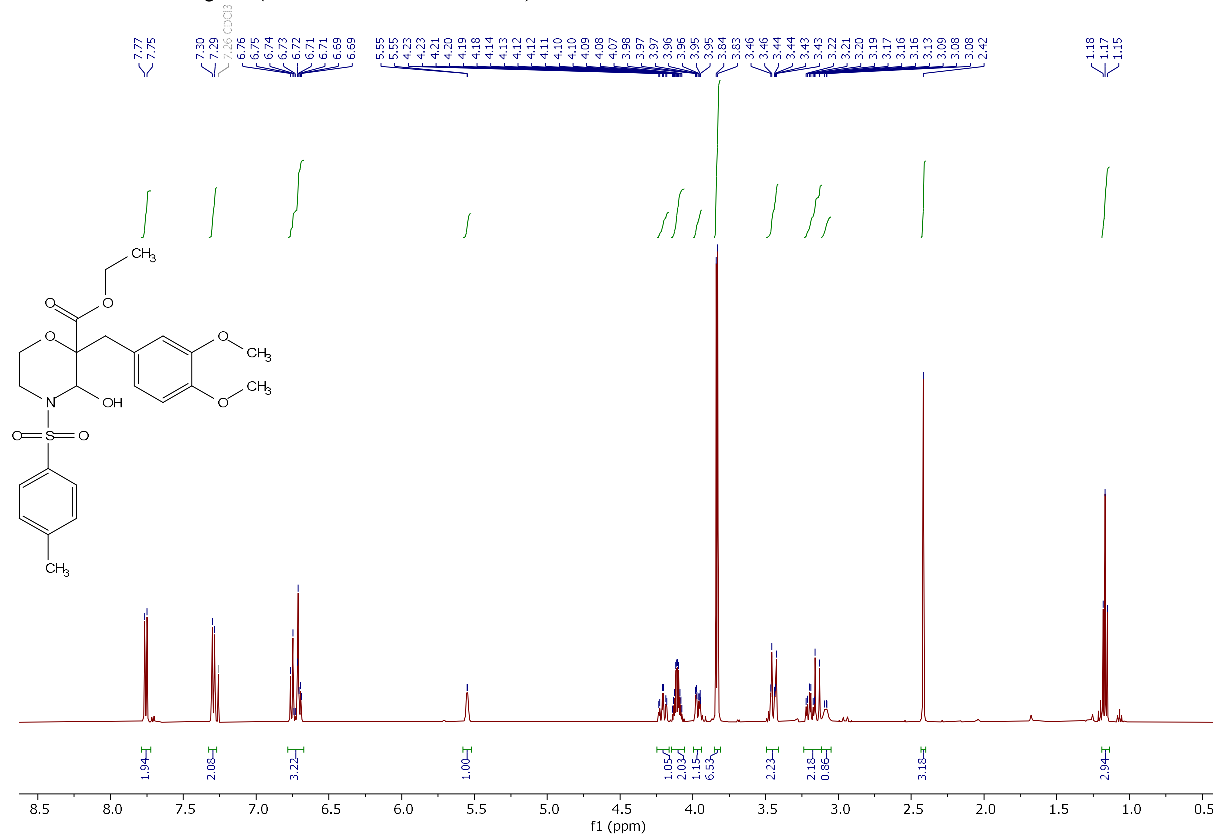

^{13}C{^{1}H} NMR of **3j''** (126 MHz, $CDCl_3$)

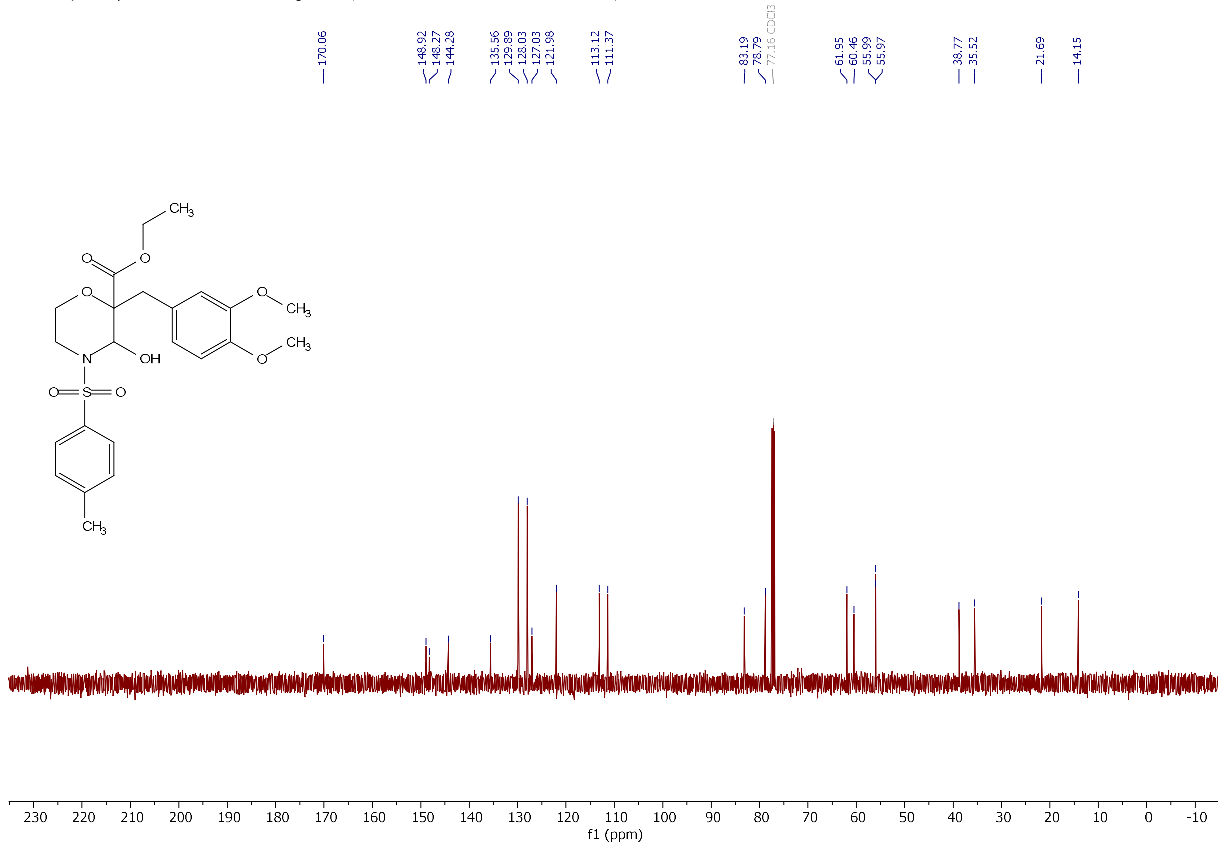

^{1}H NMR of **3k** (500 MHz, $CDCl_3$)

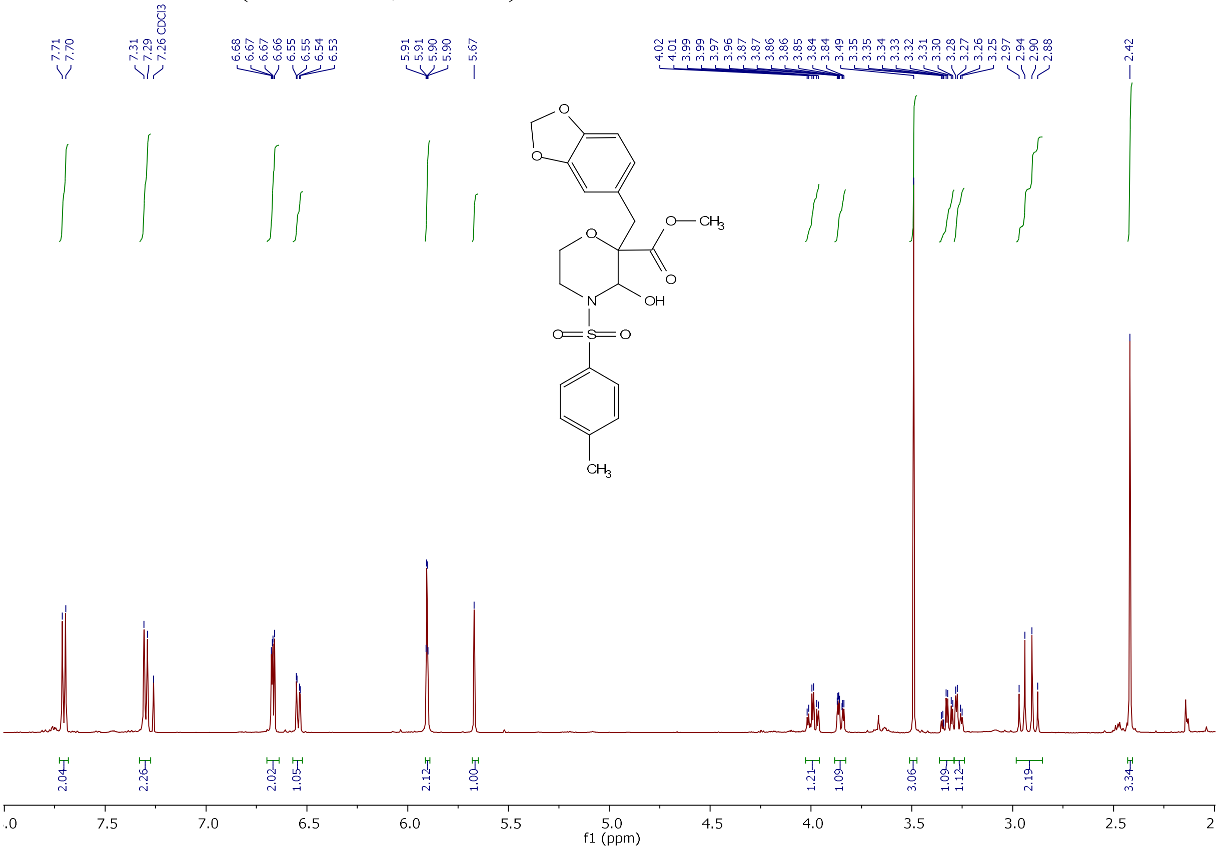

^{13}C{^{1}H} NMR of **3k** (126 MHz, $CDCl_3$)

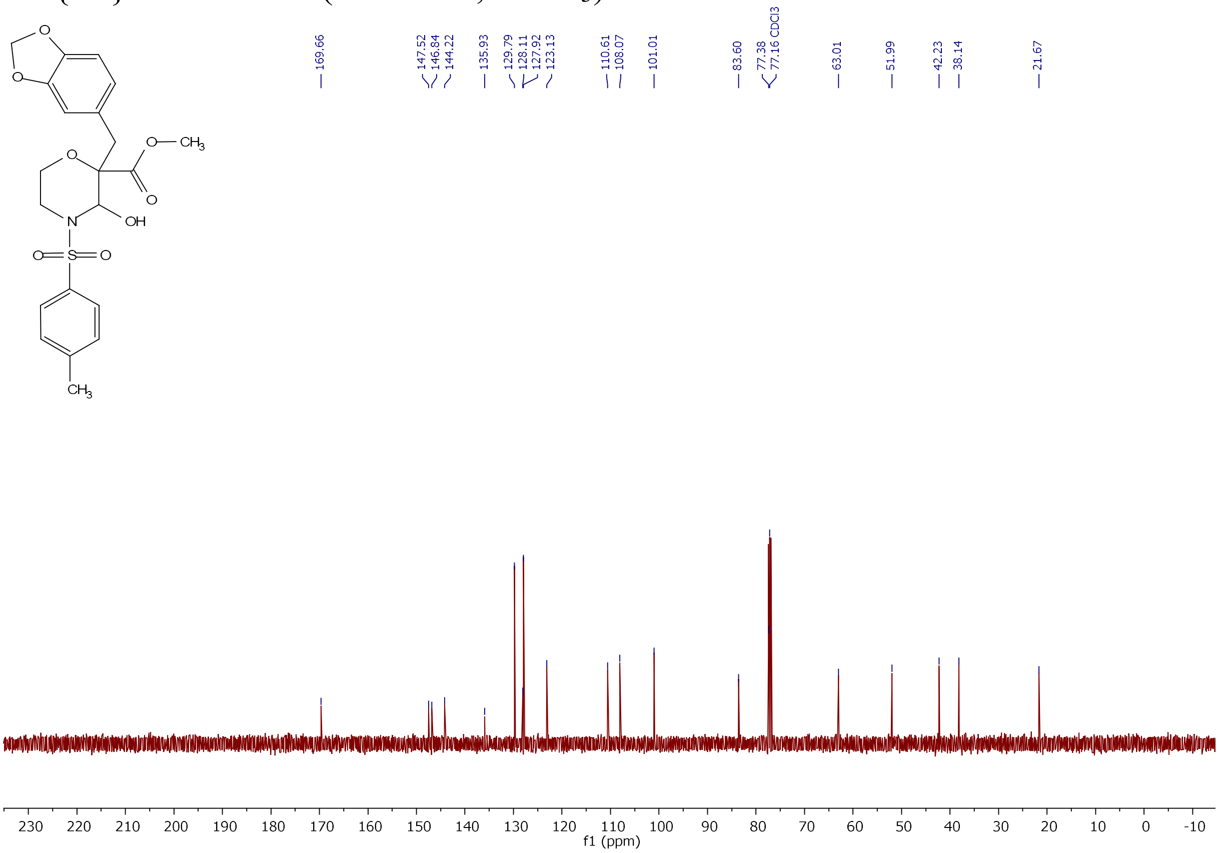

^{1}H NMR of **3l** (500 MHz, $CDCl_3$)

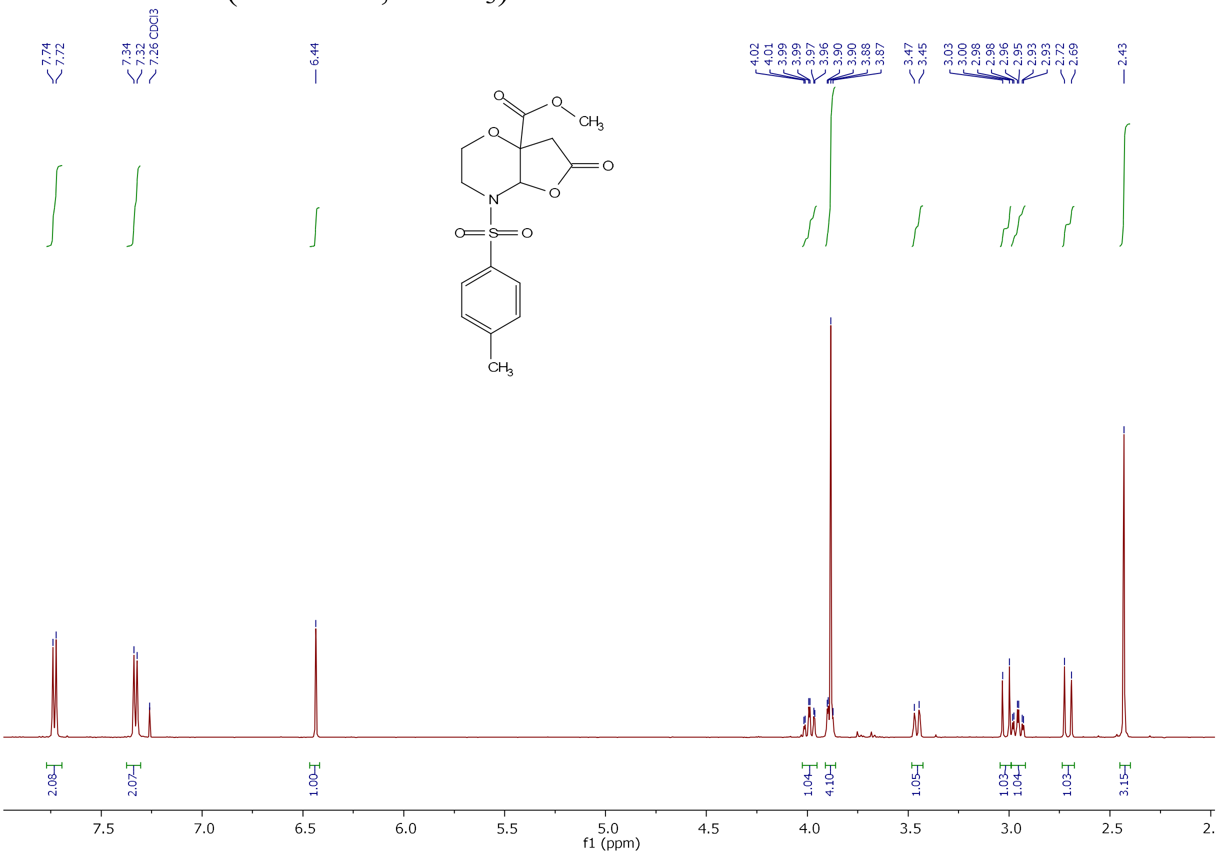

$^{13}C\{^{1}H\}$ NMR of **3l** (126 MHz, $CDCl_3$)

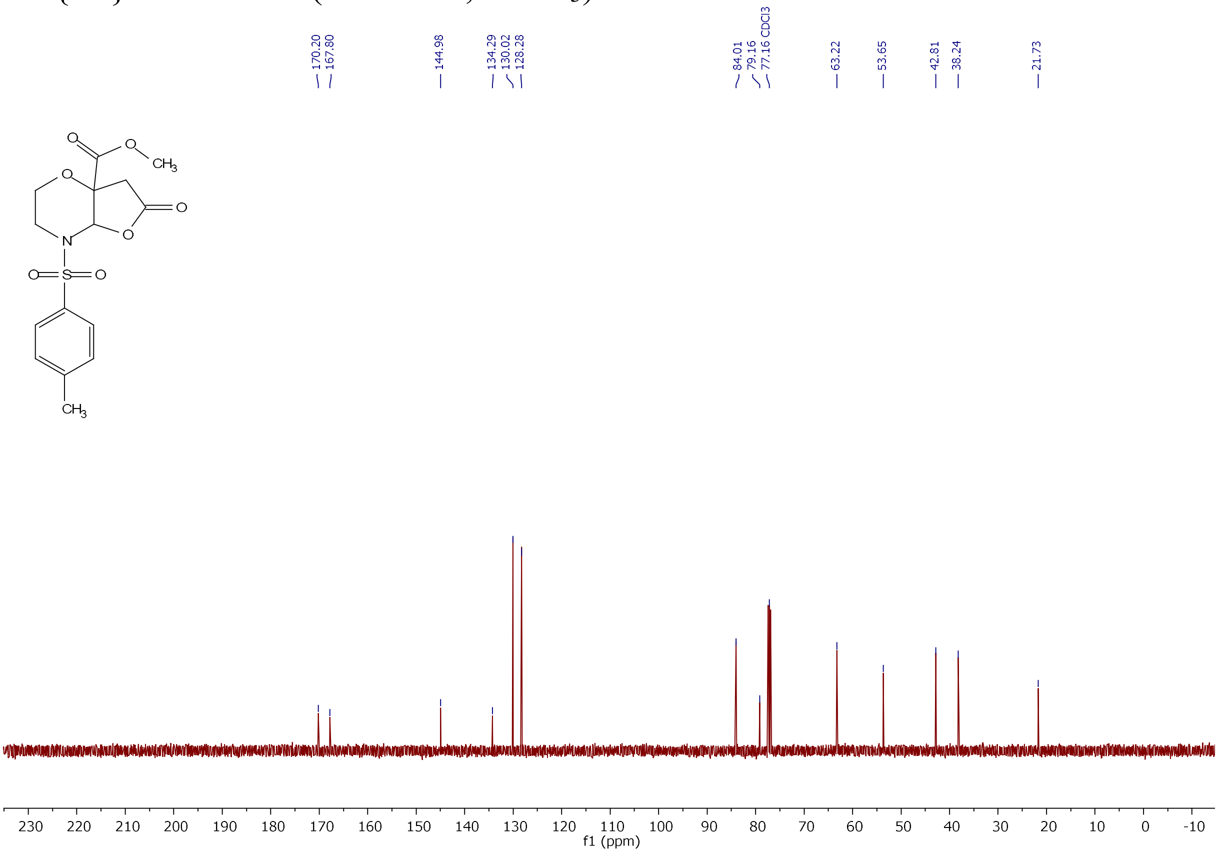

^{1}H NMR of **5a** (500 MHz, $CDCl_3$)

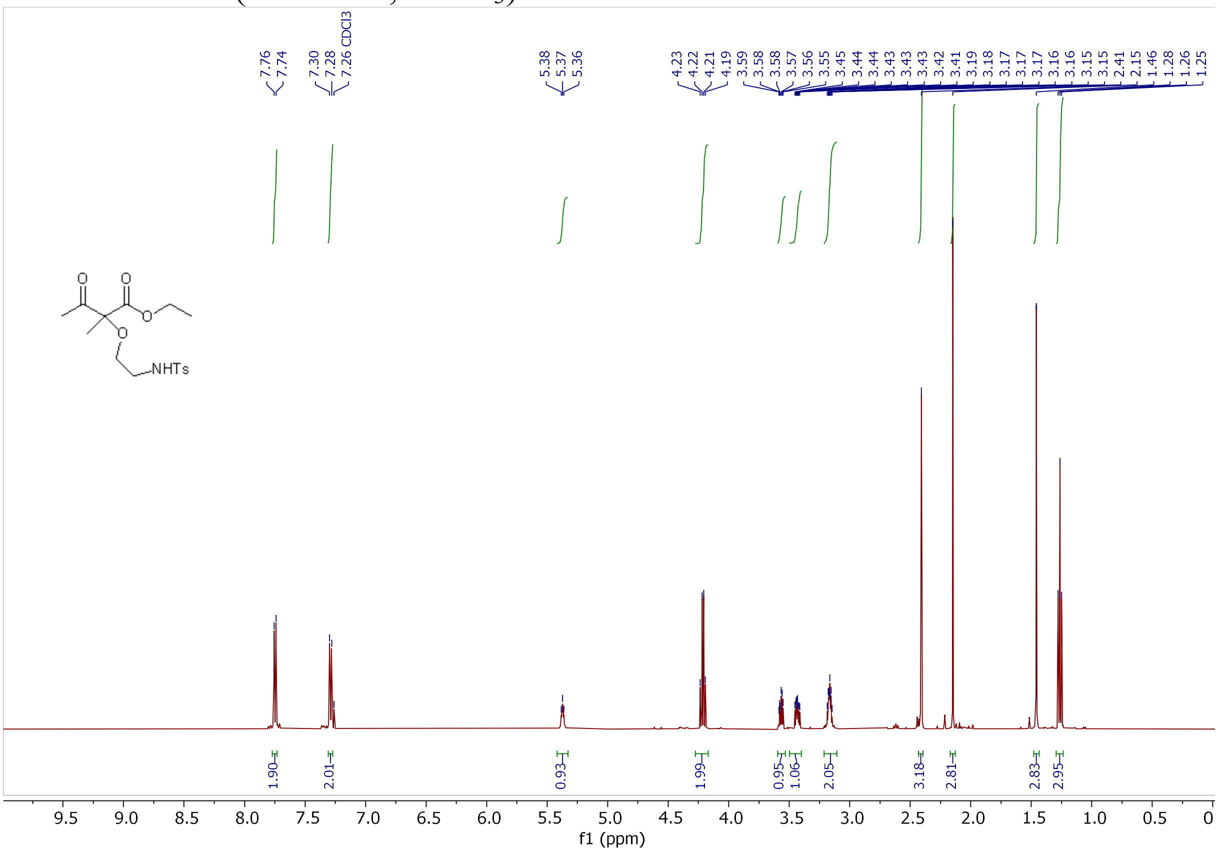

^{13}C{^{1}H} NMR of **5a** (126 MHz, $CDCl_3$)

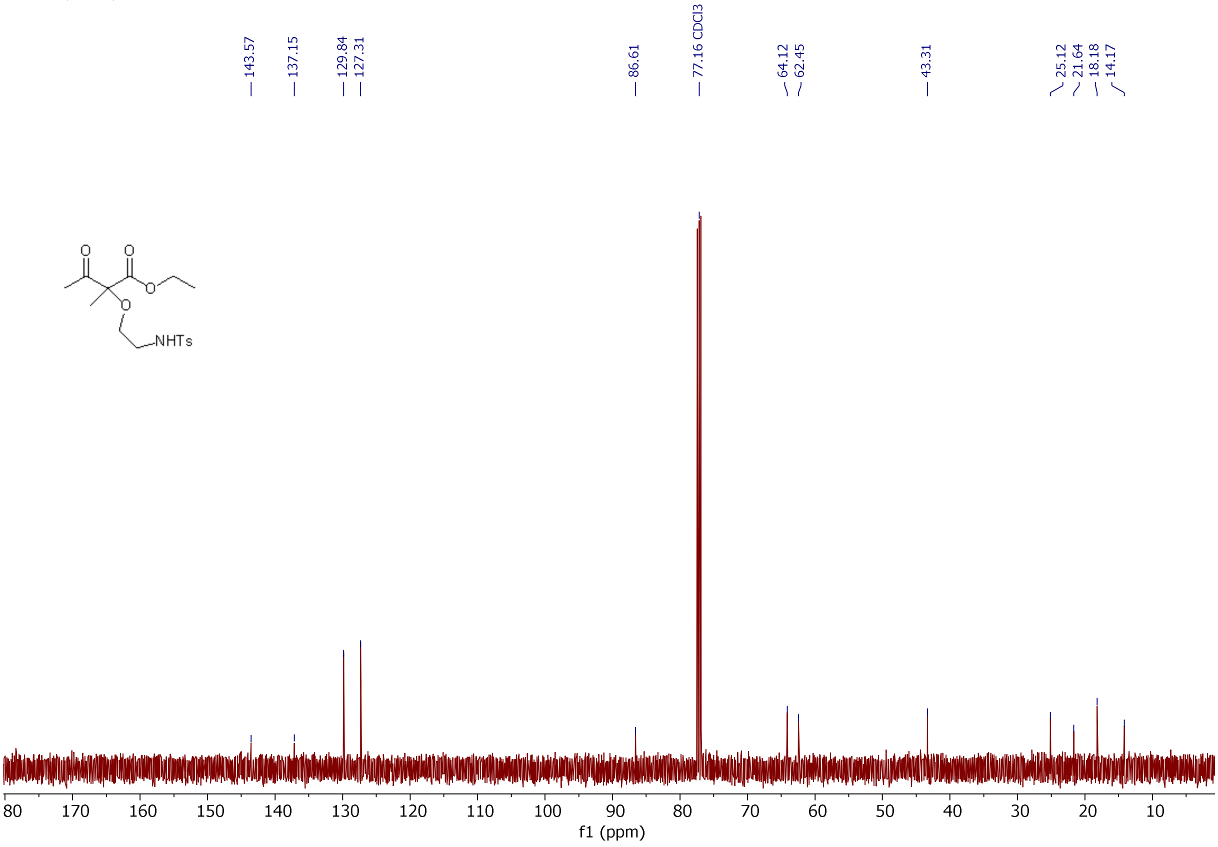

^{1}H NMR of **5b** (500 MHz, $CDCl_3$)

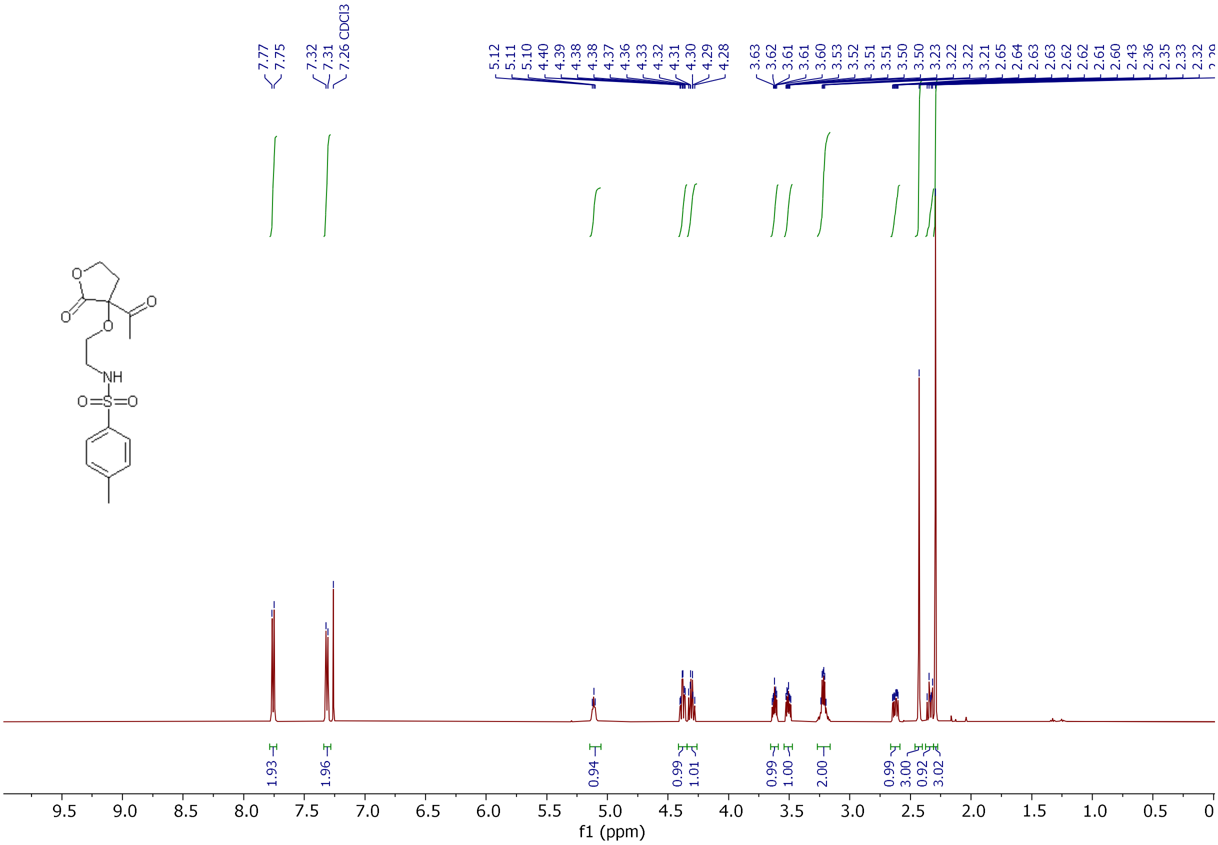

$^{13}C\{^{1}H\}$ NMR of **5b** (126 MHz, $CDCl_3$)

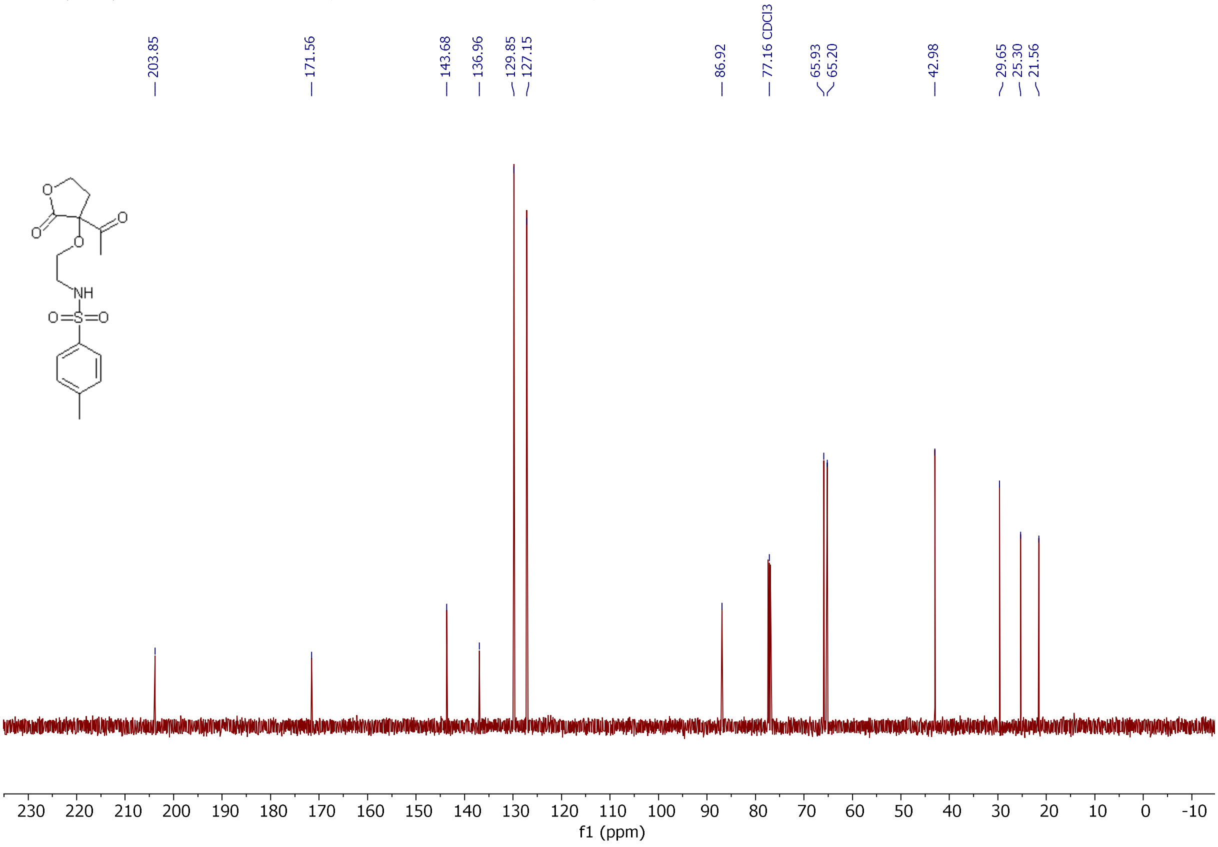

^{1}H NMR of **5c** (500 MHz, $CDCl_3$)

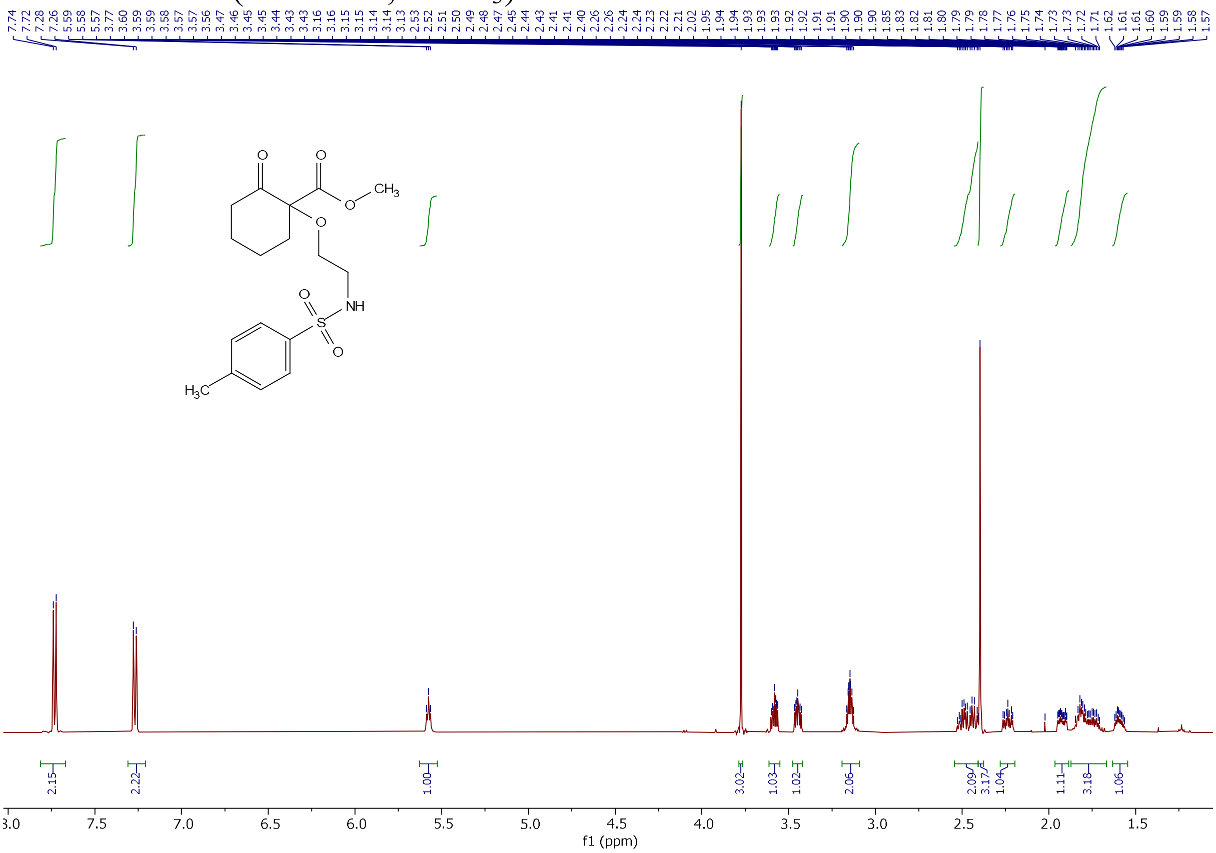

^{13}C{^{1}H} NMR of **5c** (126 MHz, $CDCl_3$)

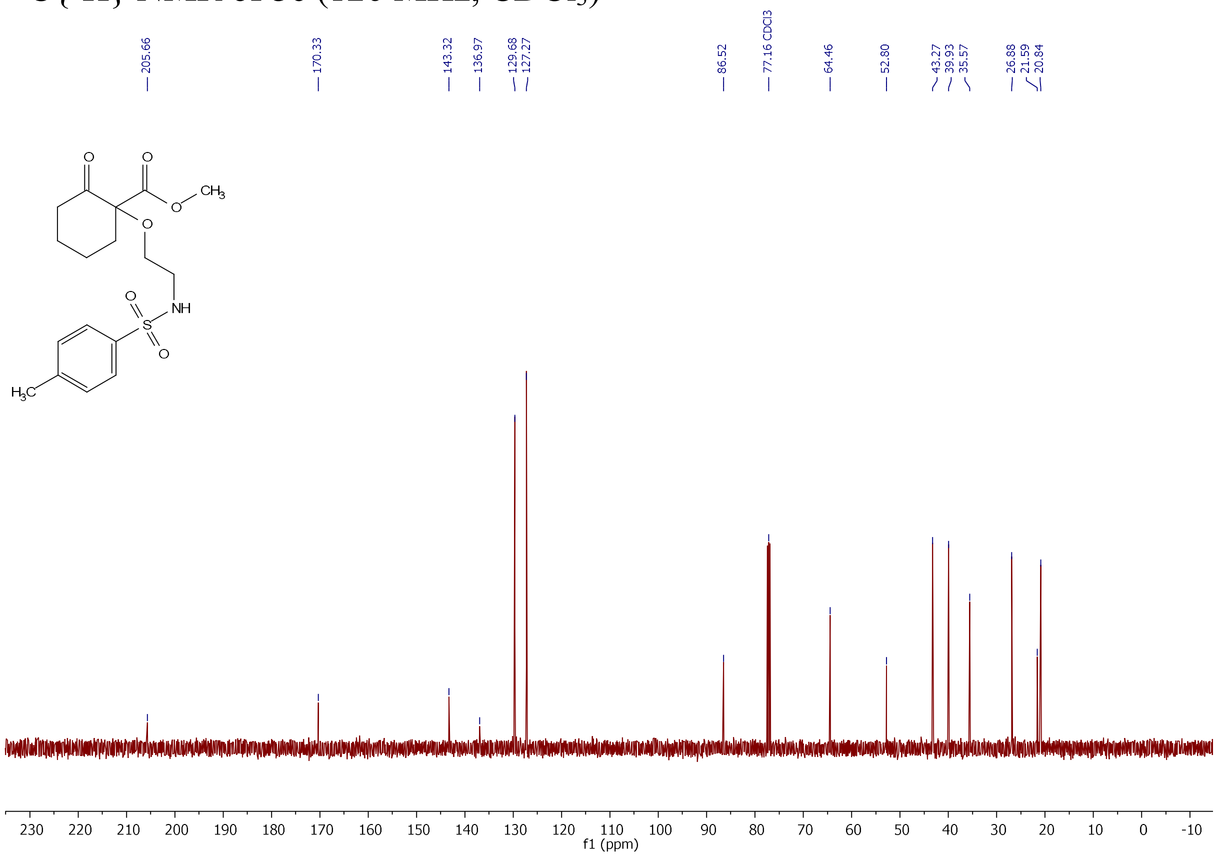

^{1}H NMR of **5d** (500 MHz, $CDCl_3$)

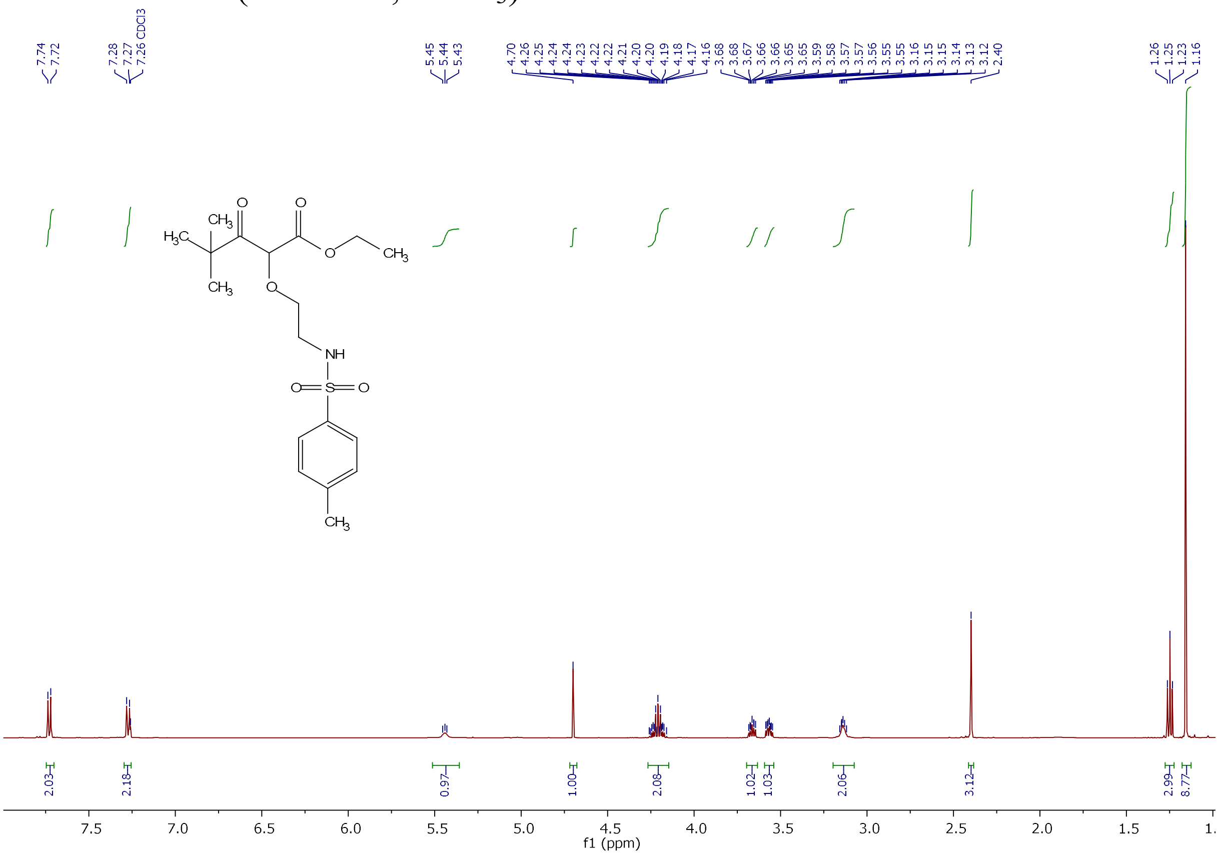

^{13}C{^{1}H} NMR of **5d** (126 MHz, $CDCl_3$)

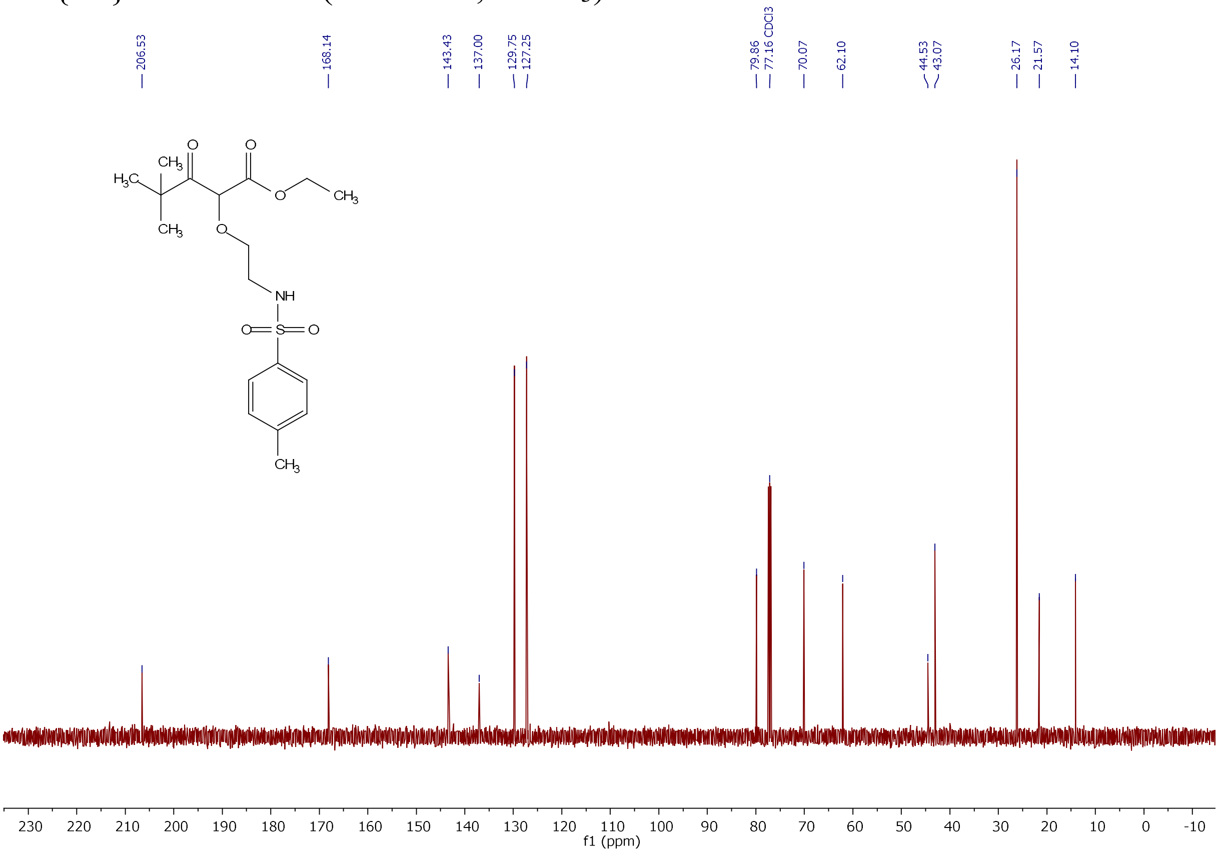

^{1}H NMR of **5e** (500 MHz, $CDCl_3$)

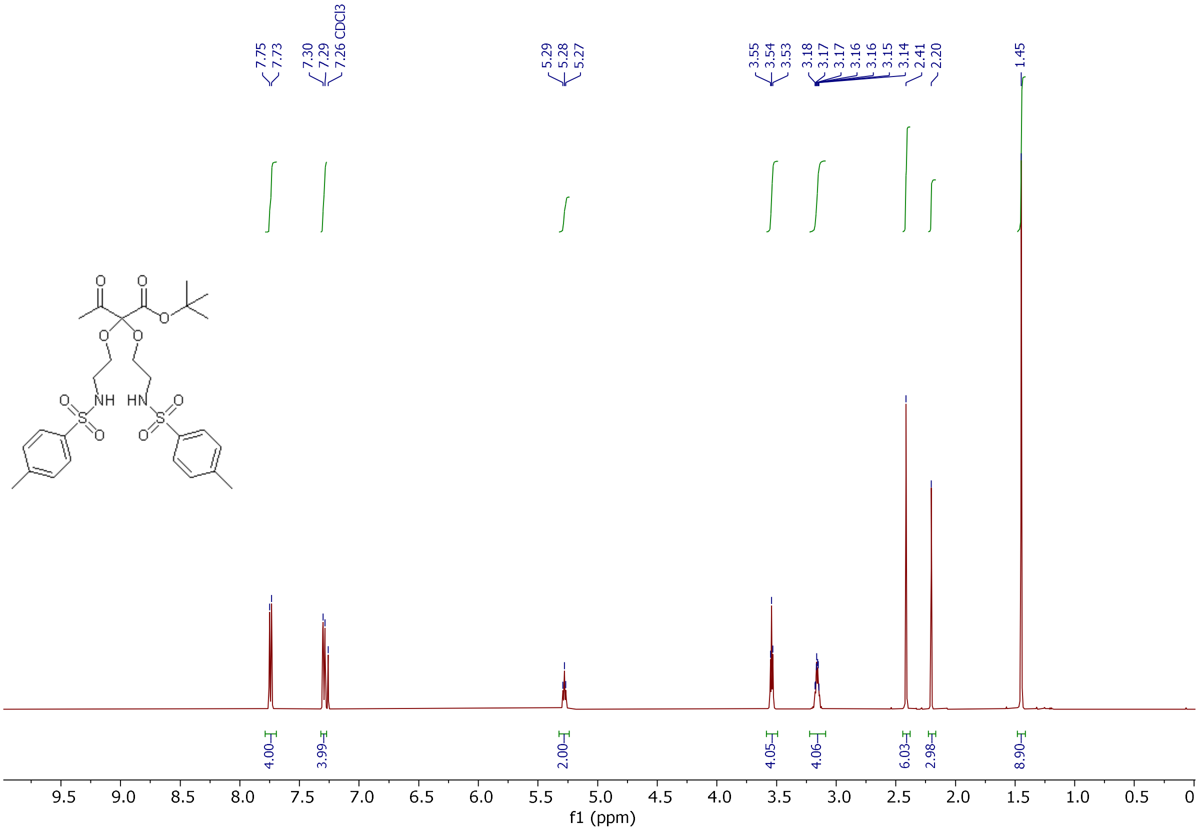

$^{13}C\{^{1}H\}$ NMR of **5e** (126 MHz, $CDCl_3$)

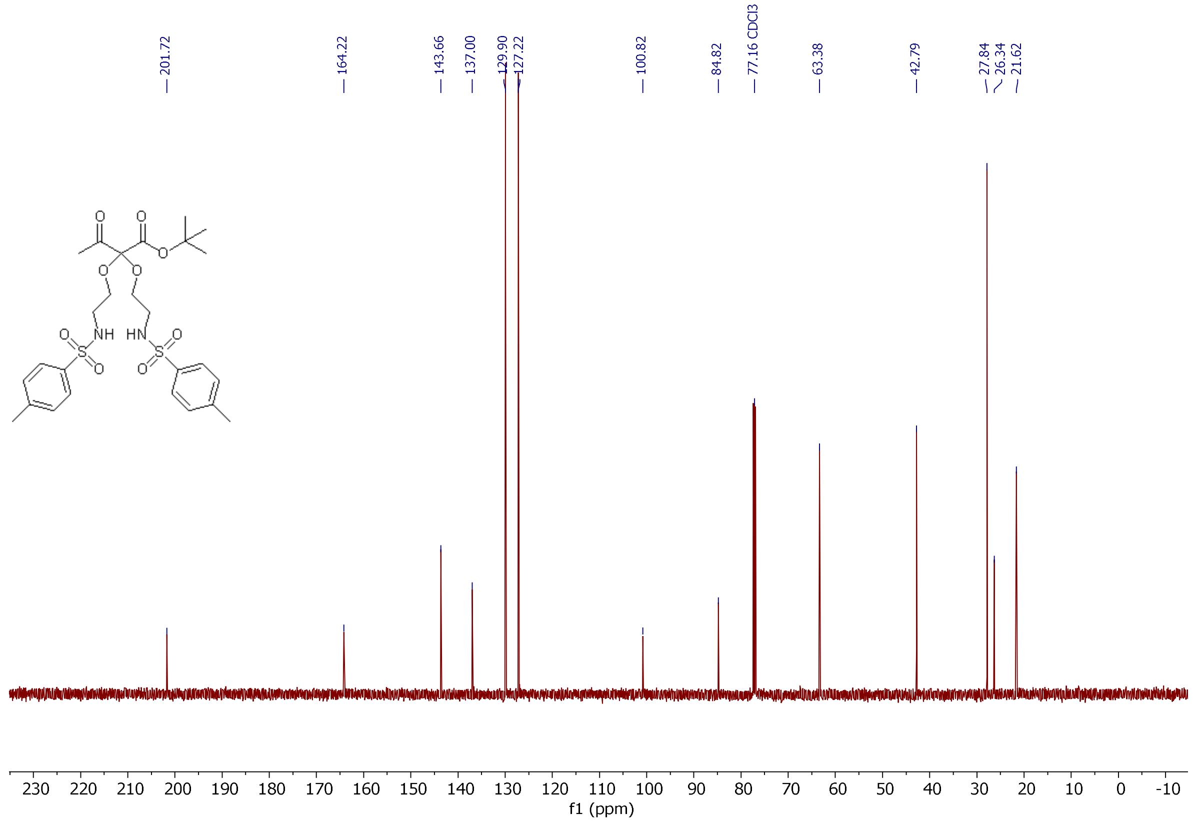

^{1}H NMR of **5f** (500 MHz, $CDCl_3$)

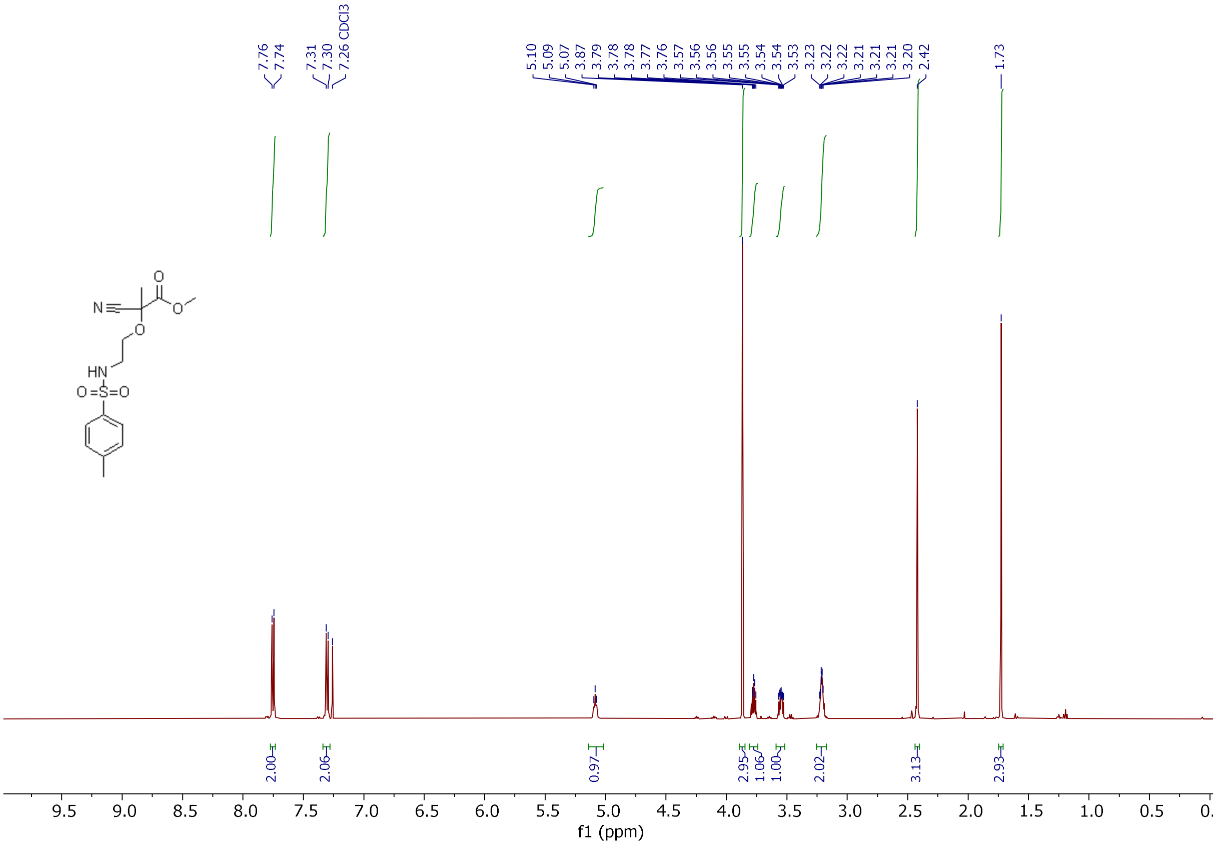

^{13}C{^{1}H} NMR of **5f** (126 MHz, $CDCl_3$)

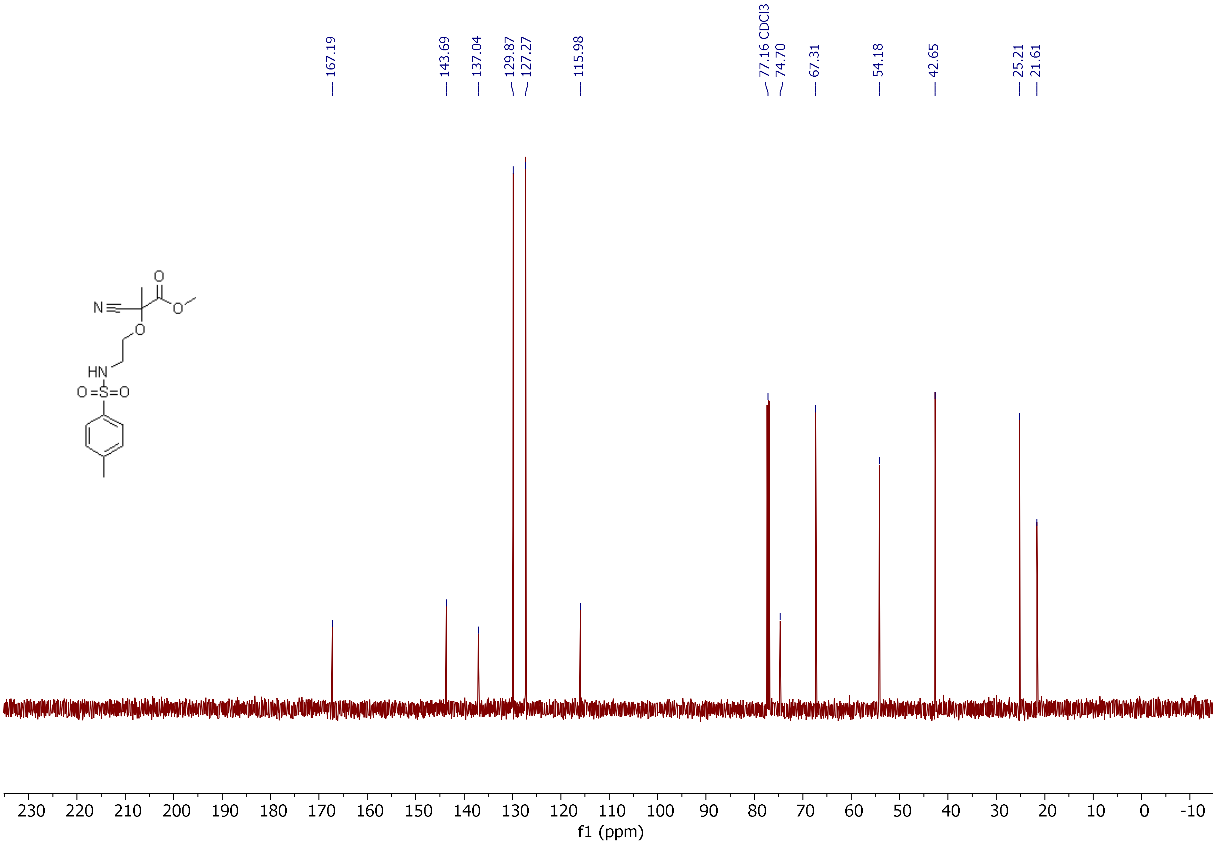

^{1}H NMR of **6** (500 MHz, $CDCl_3$)

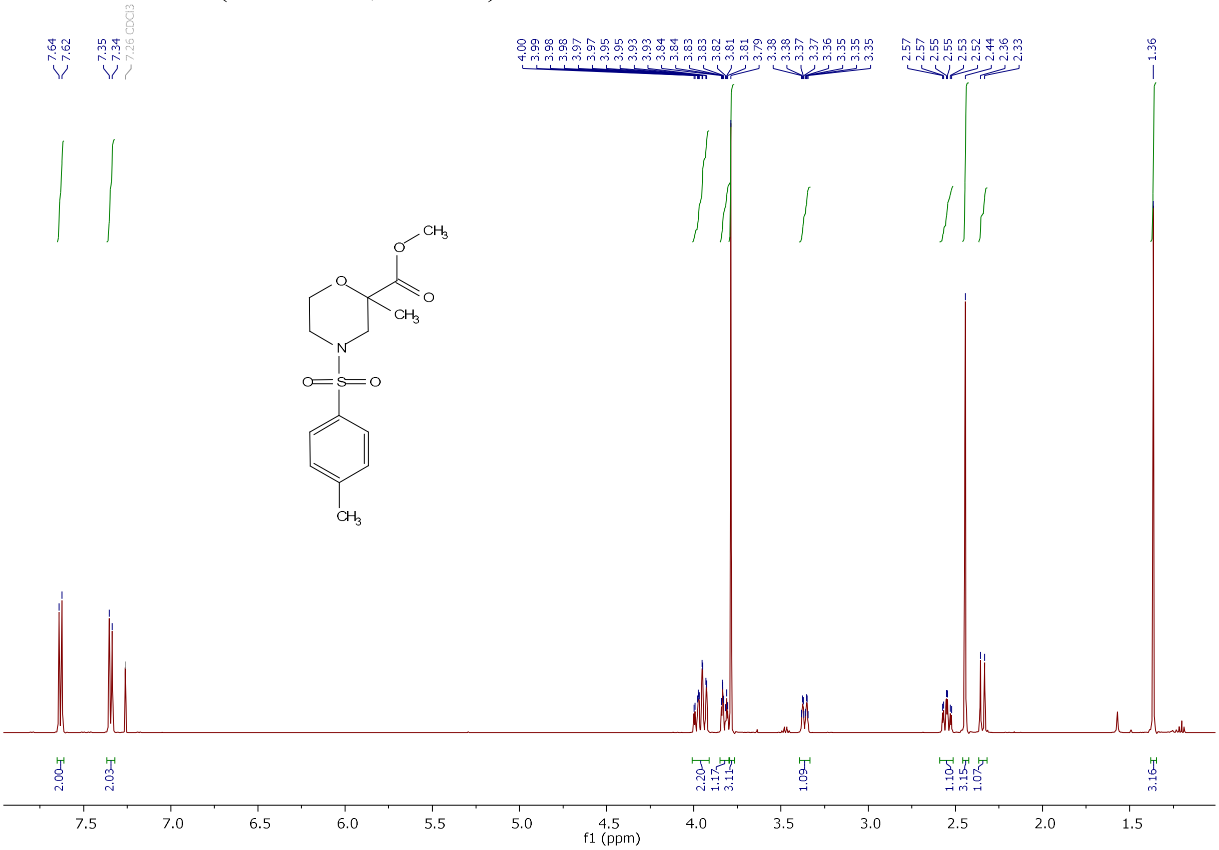

$^{13}C\{^{1}H\}$ NMR of **6** (126 MHz, $CDCl_3$)

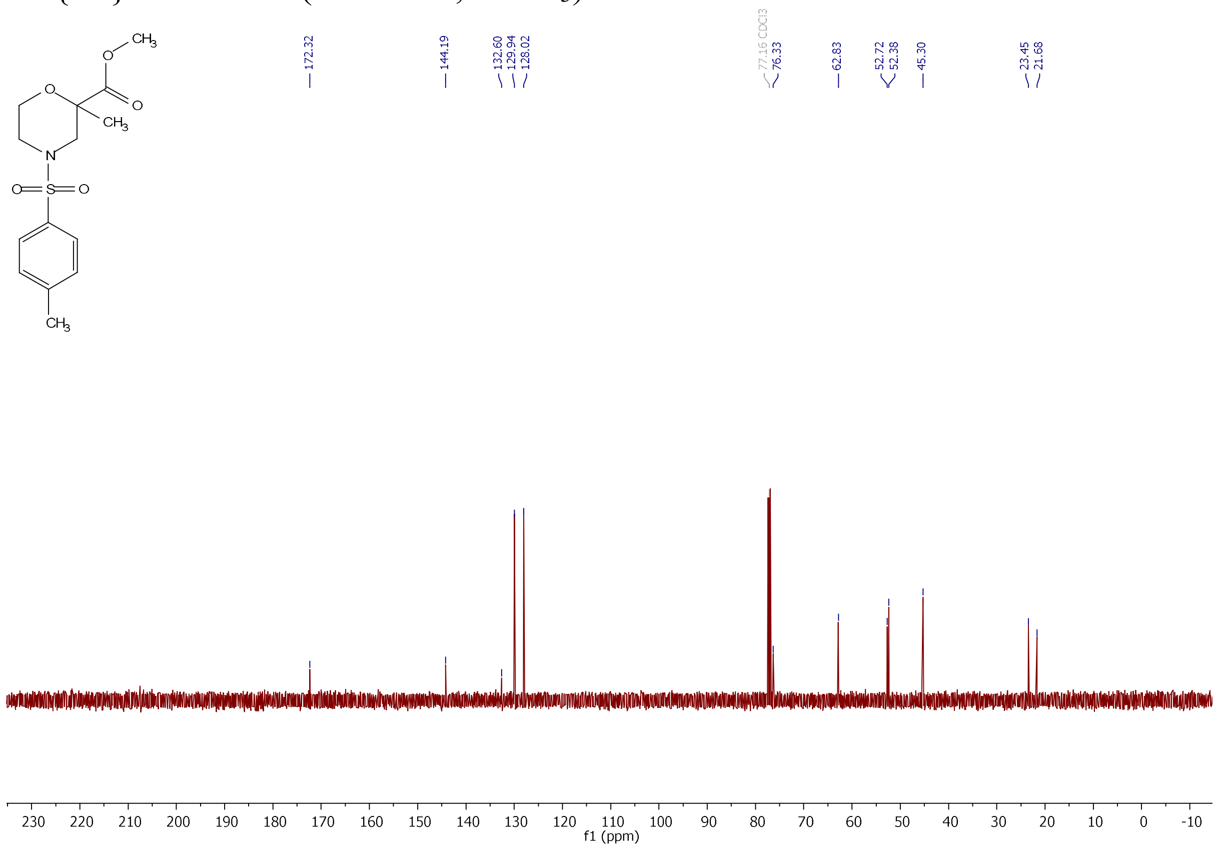

^{1}H NMR of **7** (300 MHz, $CDCl_3$)

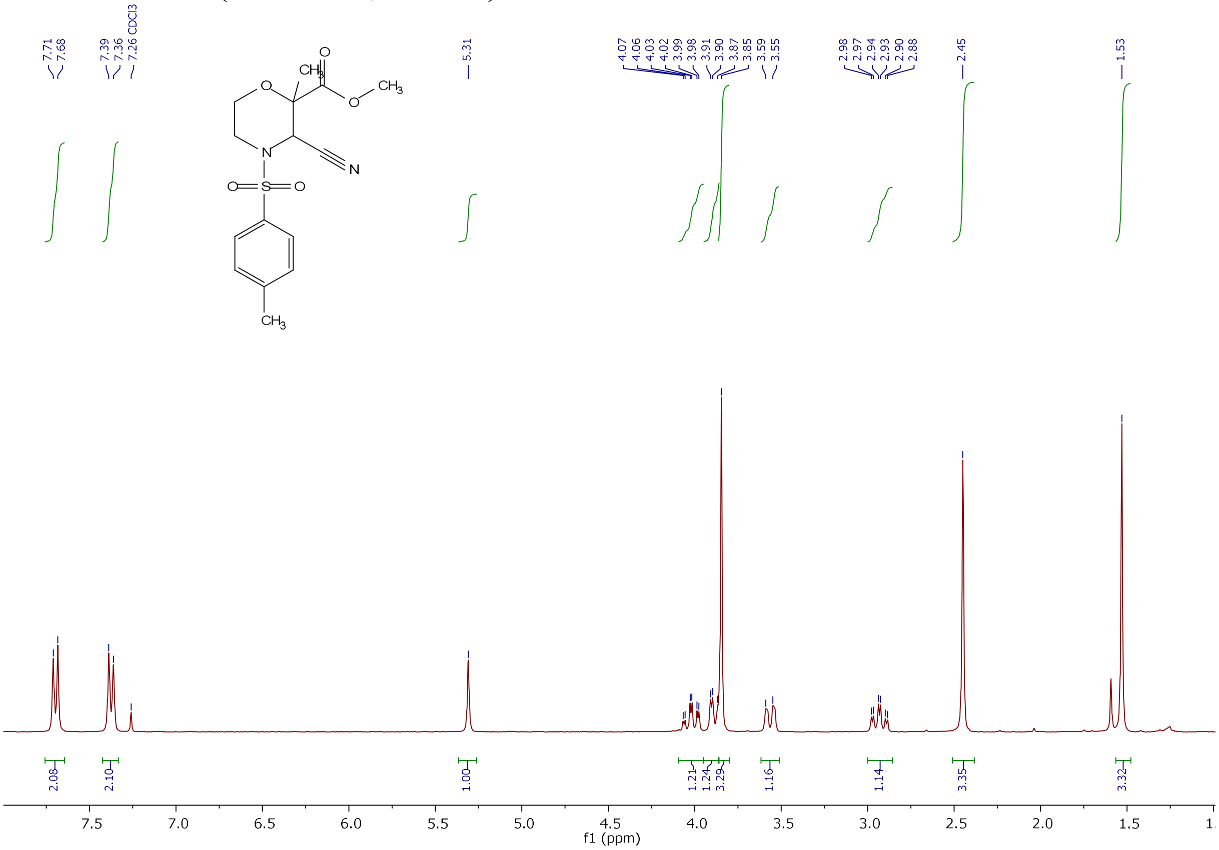

^{13}C{^{1}H} NMR of **7** (75 MHz, $CDCl_3$)

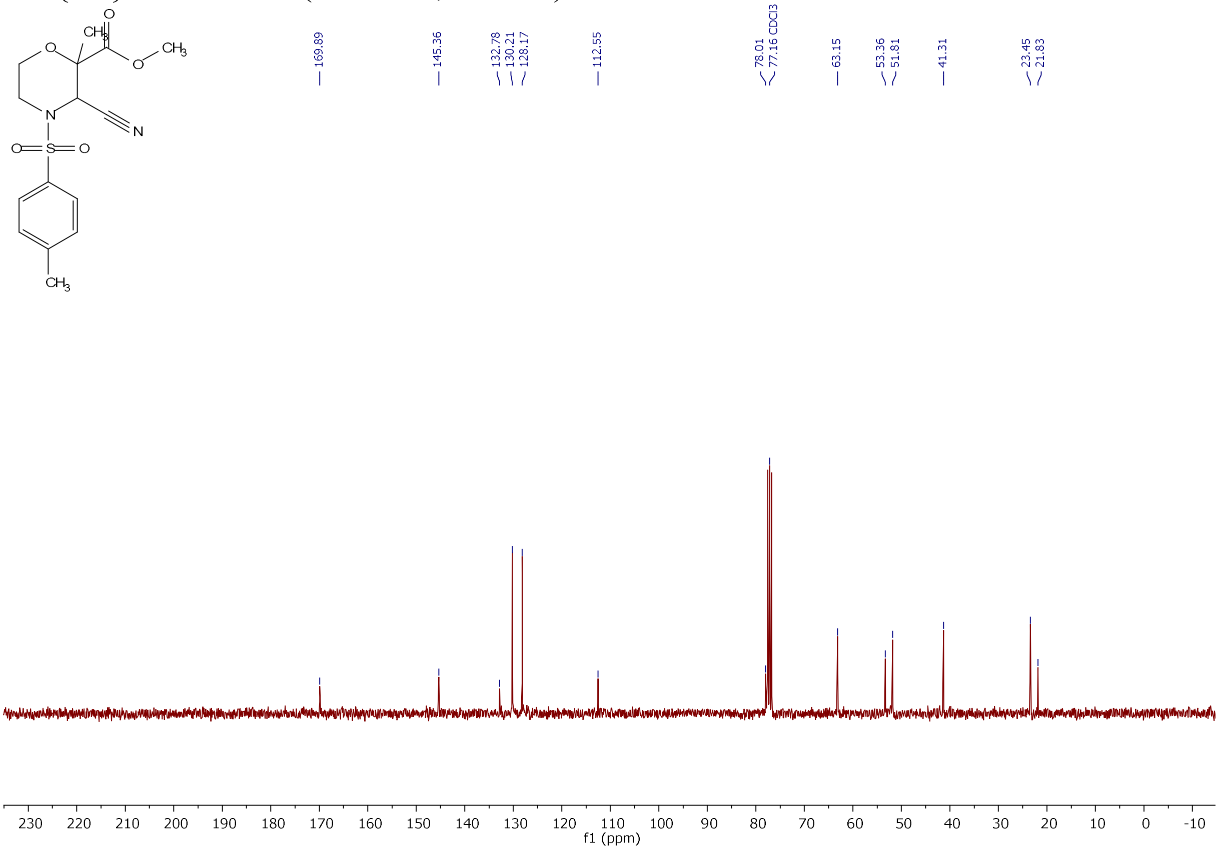

^{1}H NMR of **8** (500 MHz, $CDCl_3$)

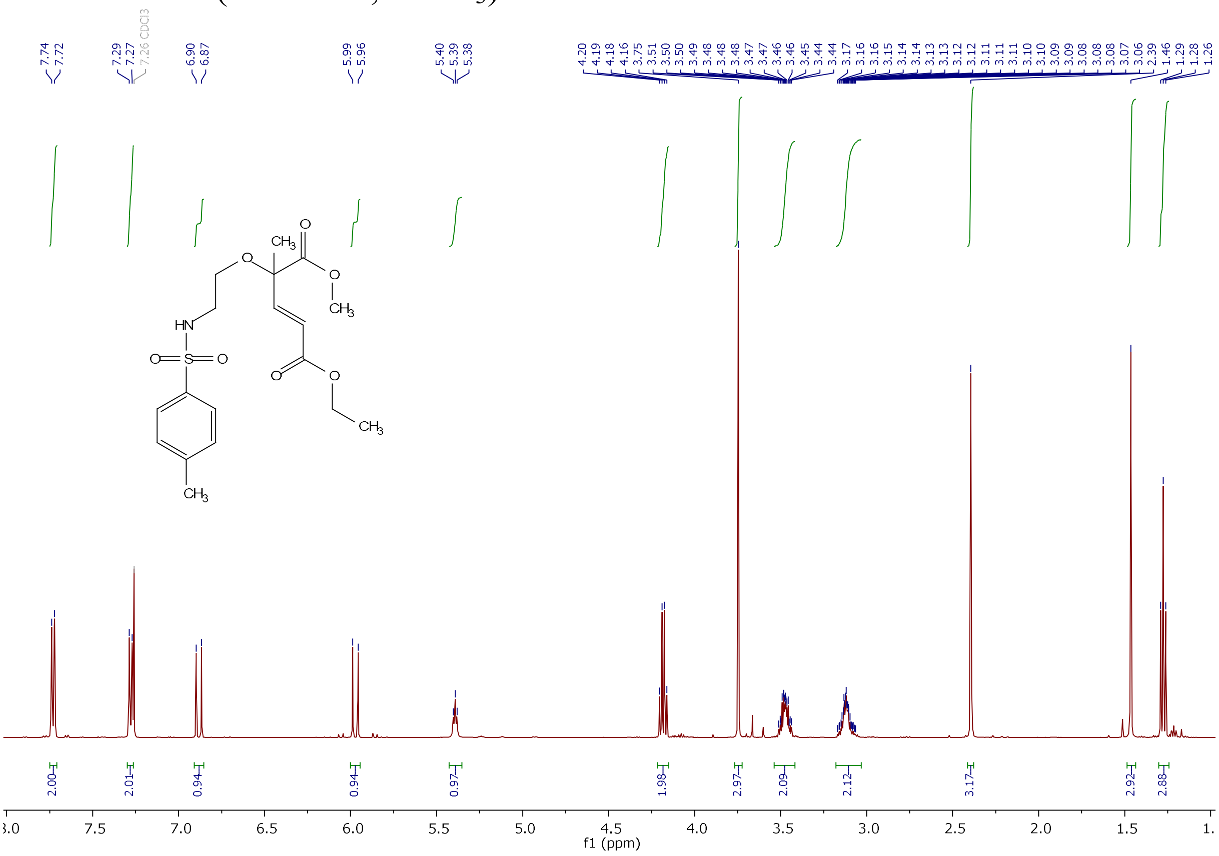

^{13}C{^{1}H} NMR of **8** (126 MHz, $CDCl_3$)

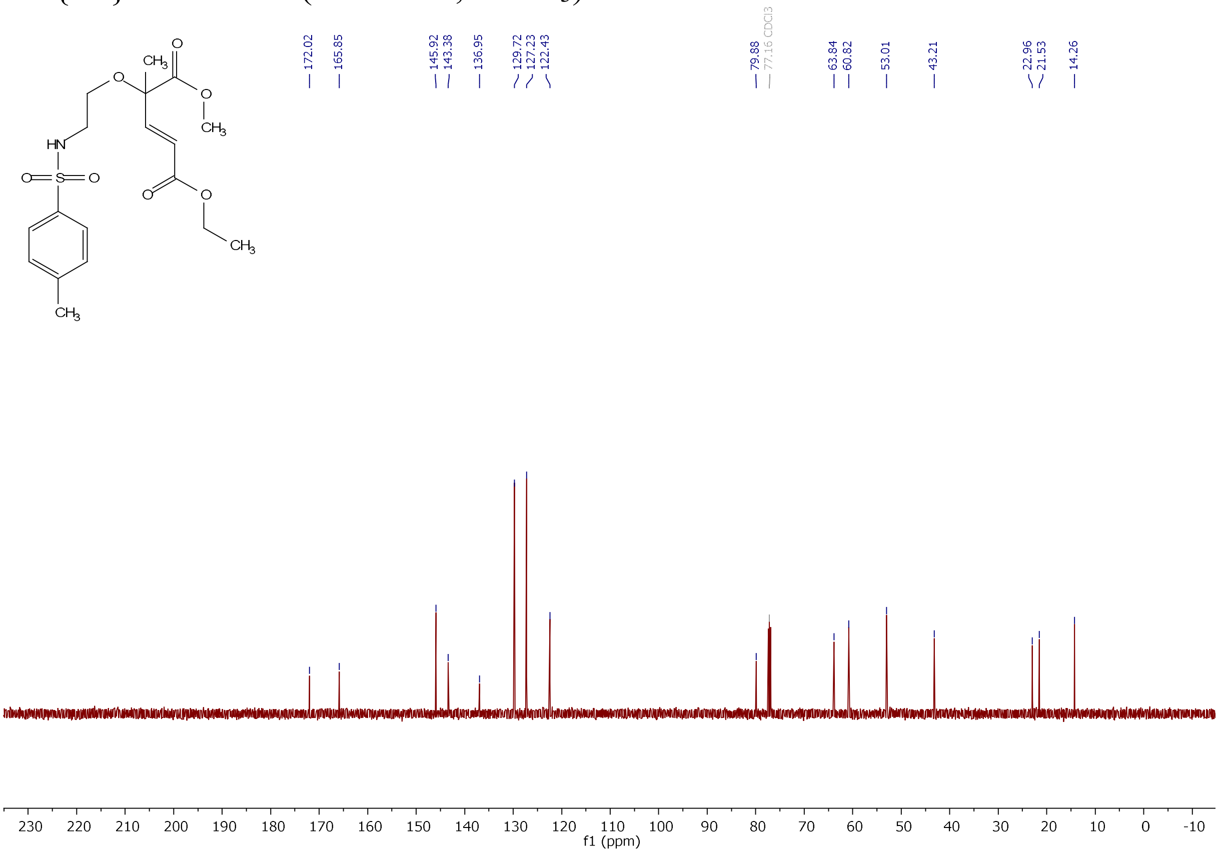

^{1}H NMR of **9** (300 MHz, $CDCl_3$)

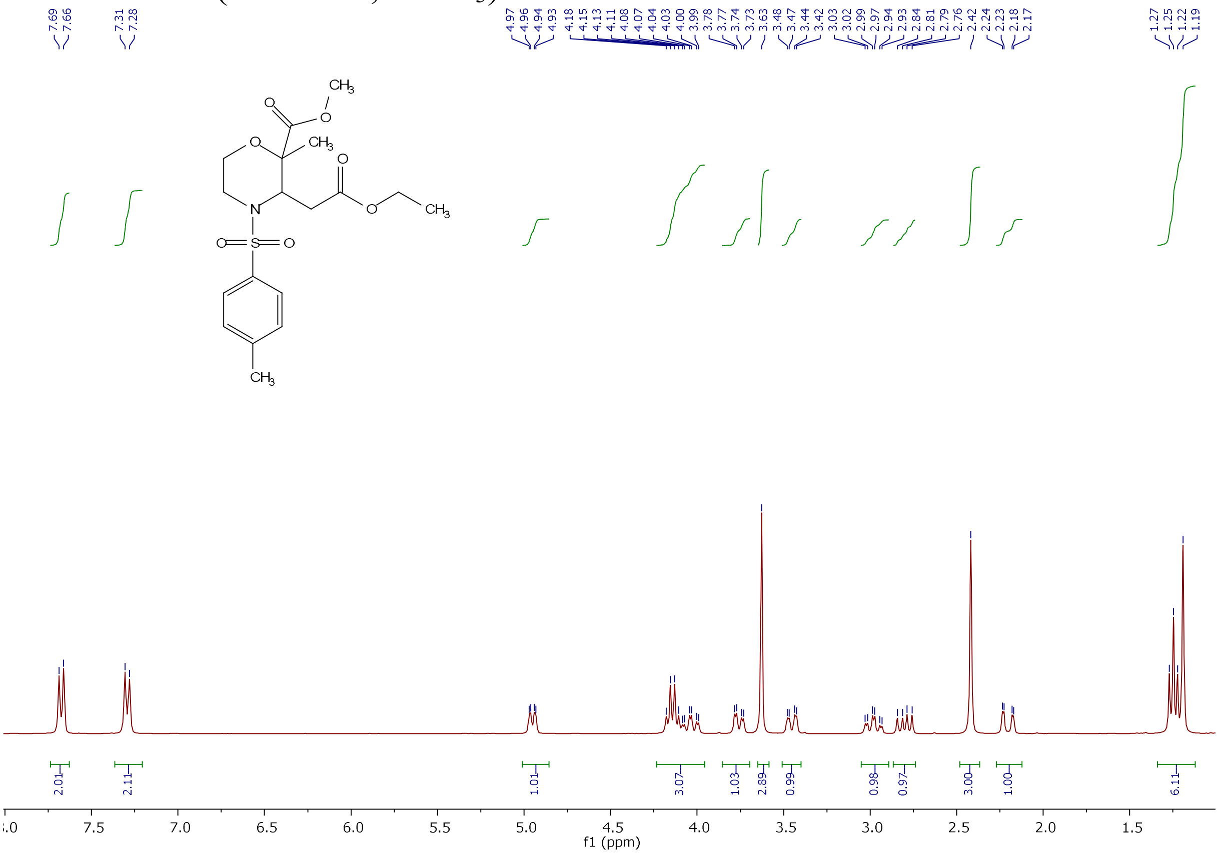

$^{13}C\{^{1}H\}$ NMR of **9** (75 MHz, $CDCl_3$)

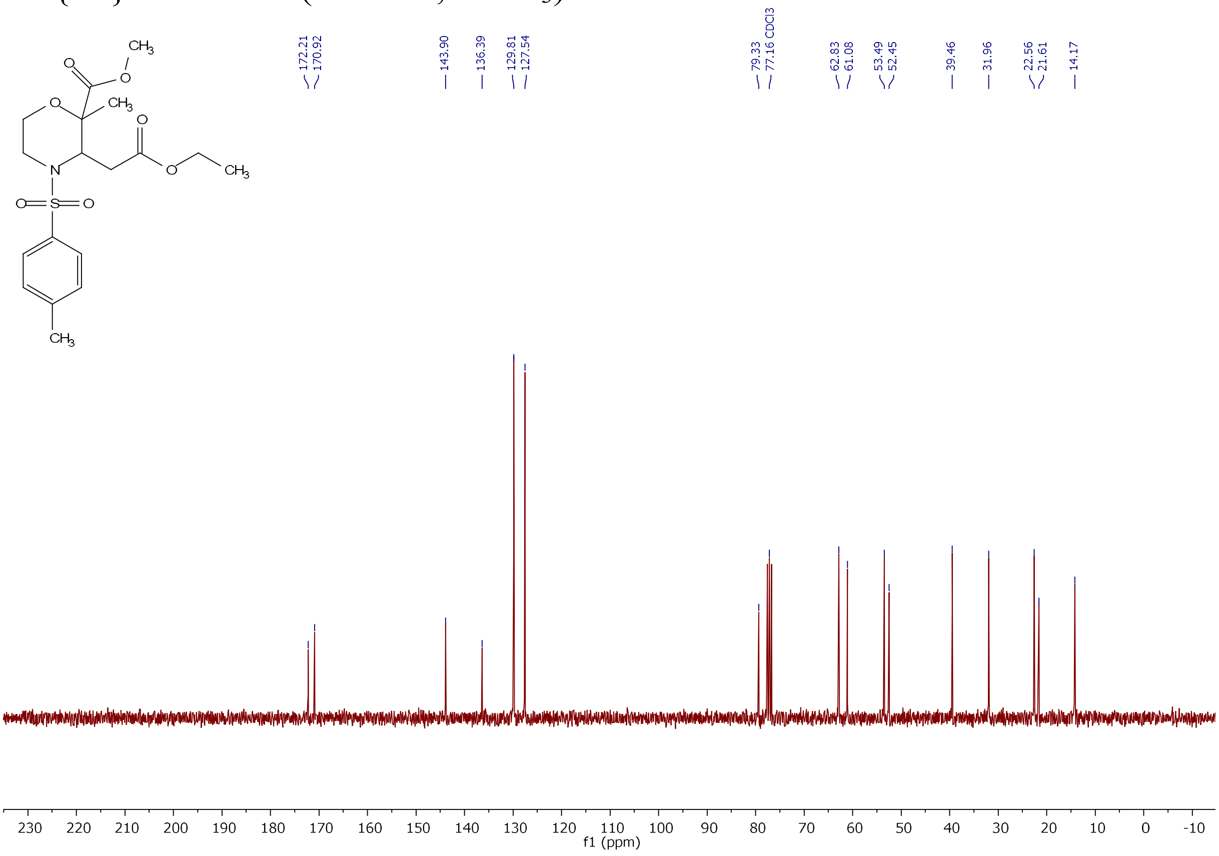

Key ROESY correlations of compound **9**

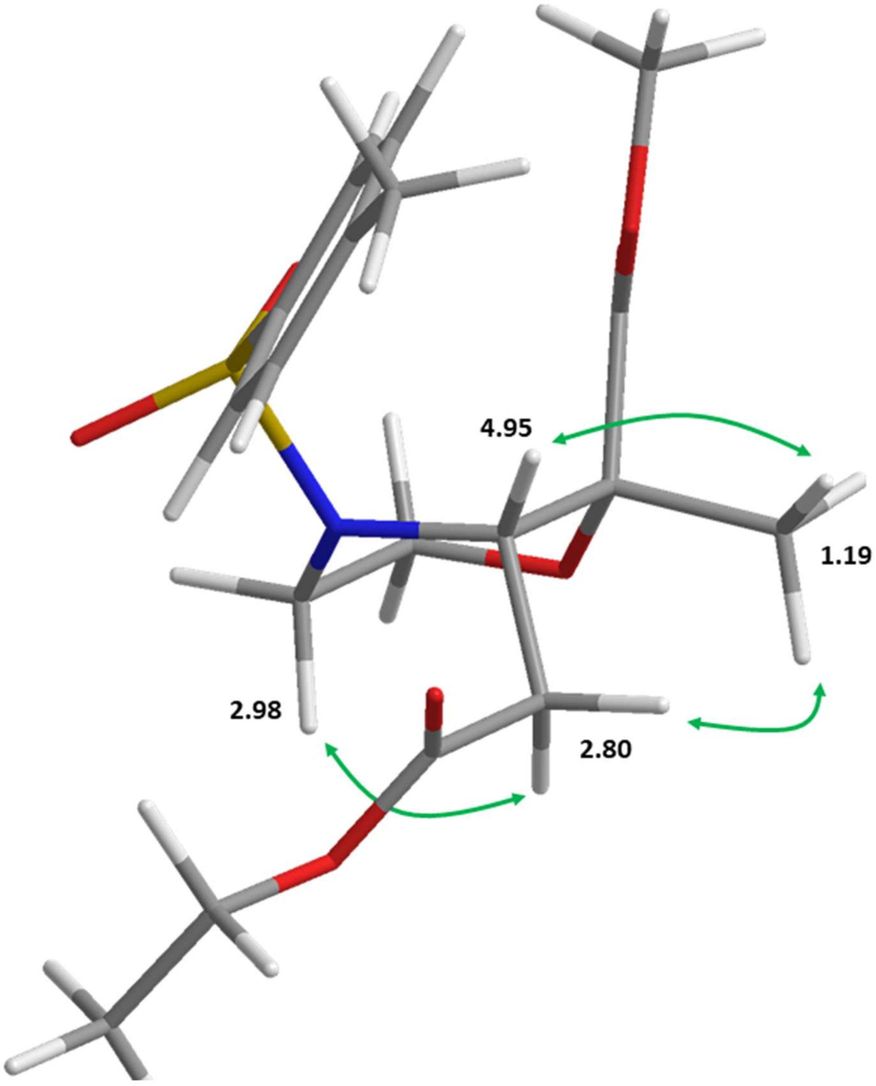

^{1}H NMR of **10’** (500 MHz, $CDCl_3$)

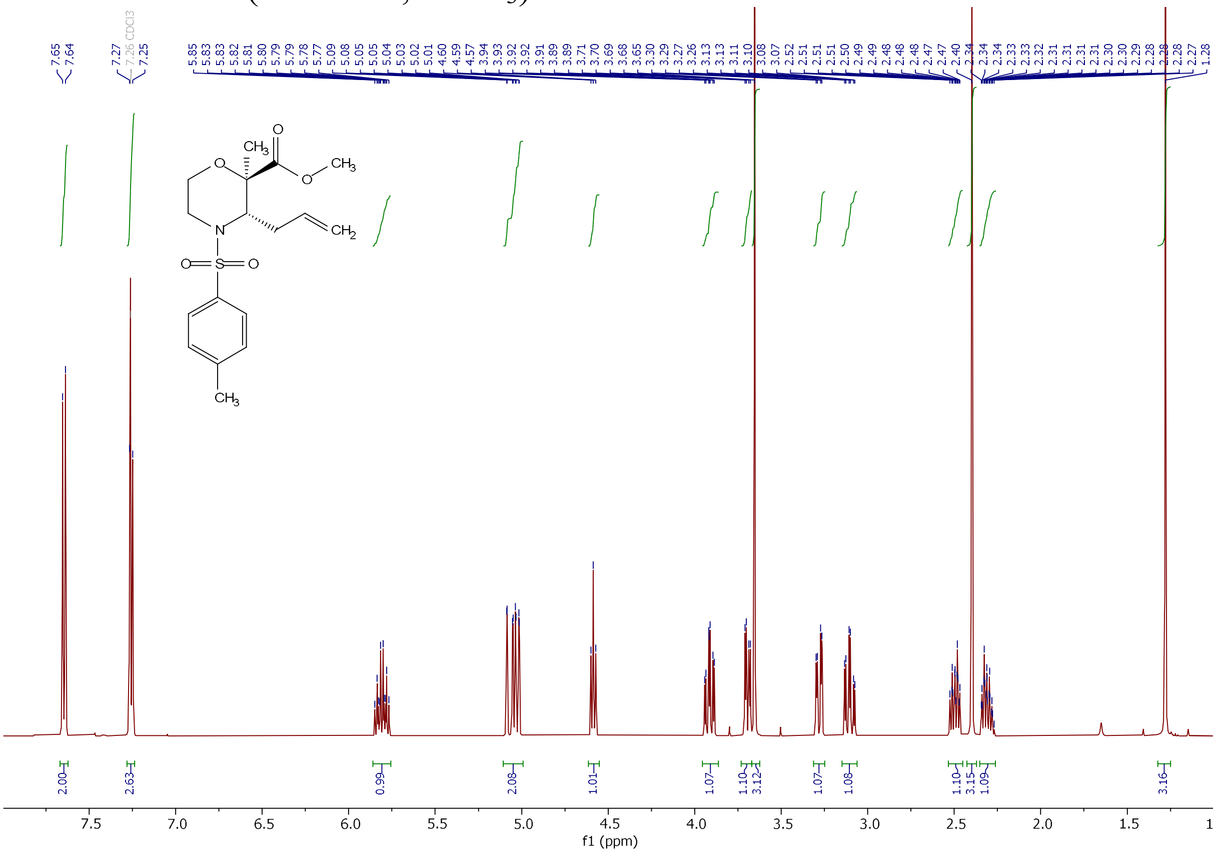

^{13}C{^{1}H} NMR of **10’** (126 MHz, $CDCl_3$)

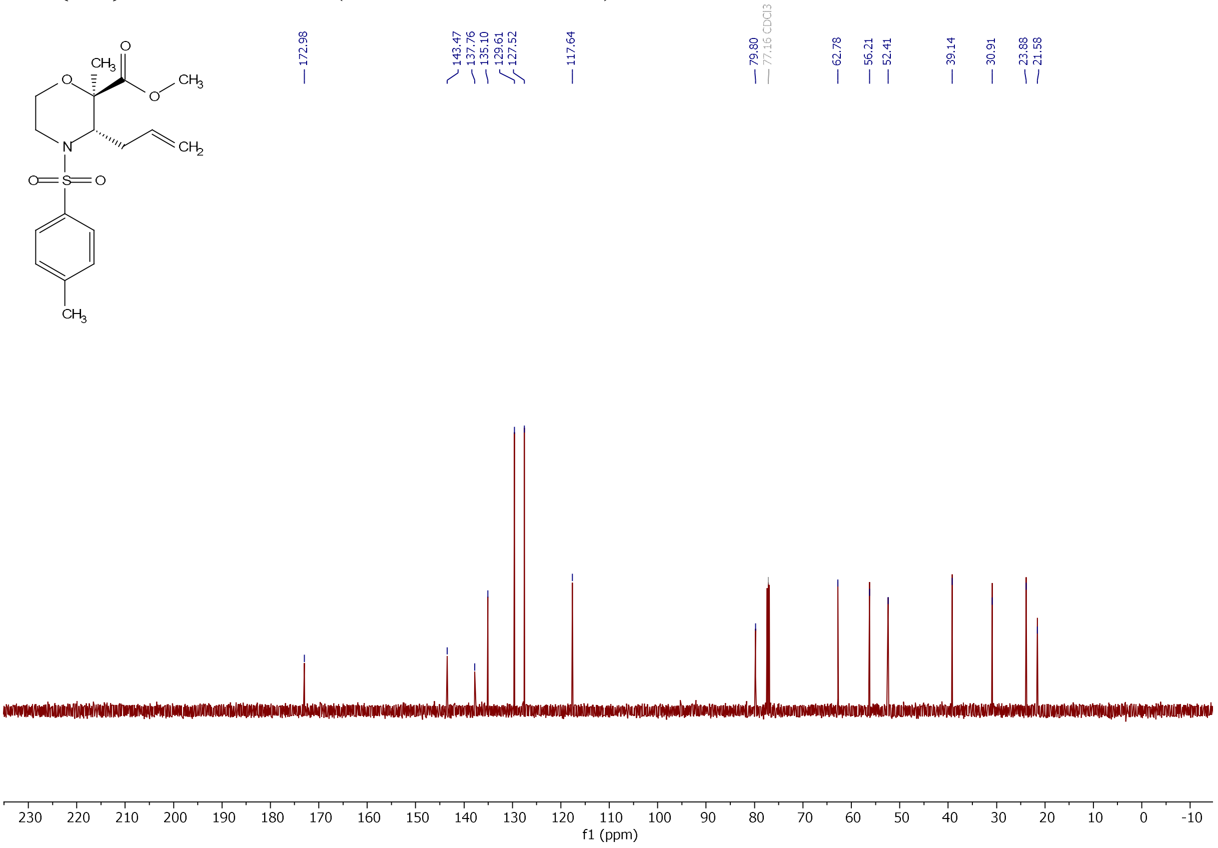

^{1}H NMR of **10’’** (500 MHz, $CDCl_3$)

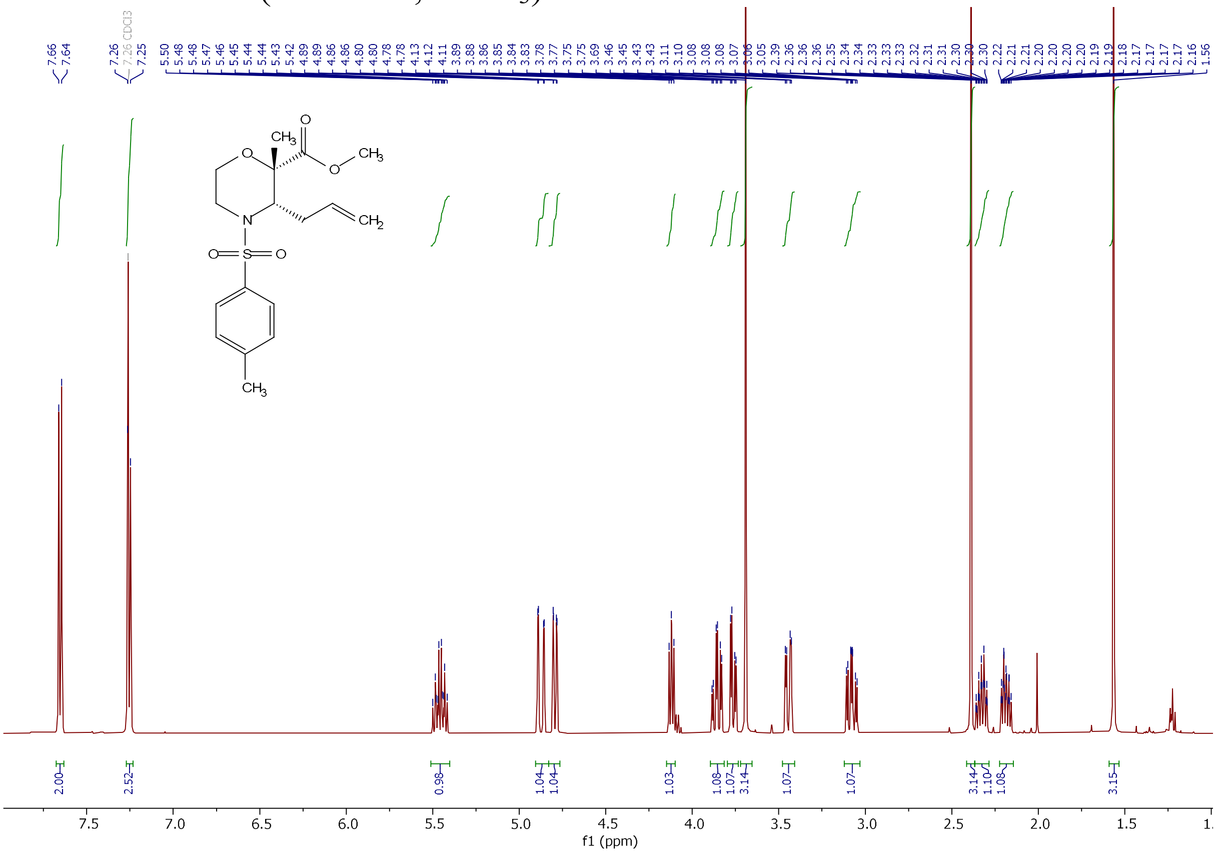

^{13}C{^{1}H} NMR of **10’’** (126 MHz, $CDCl_3$)

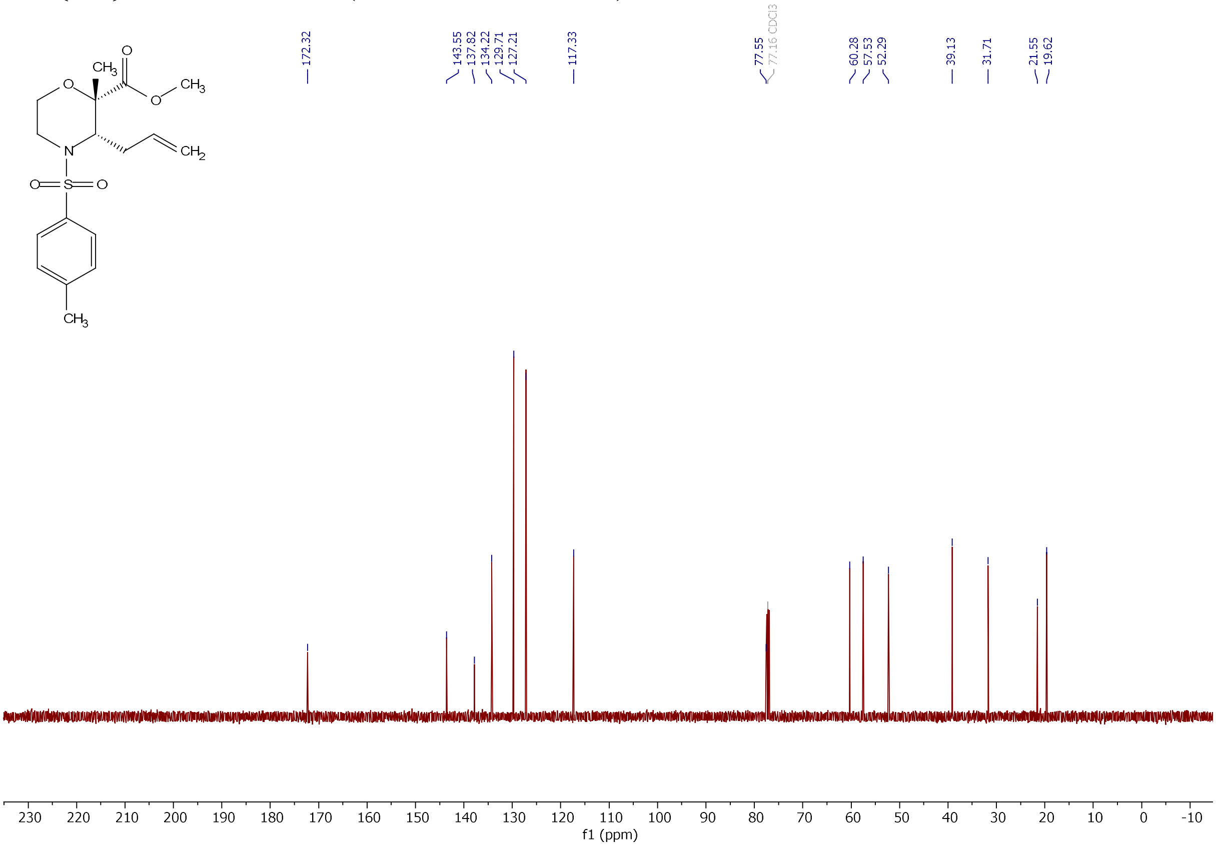

^{1}H NMR of **12** (500 MHz, $CDCl_3$)

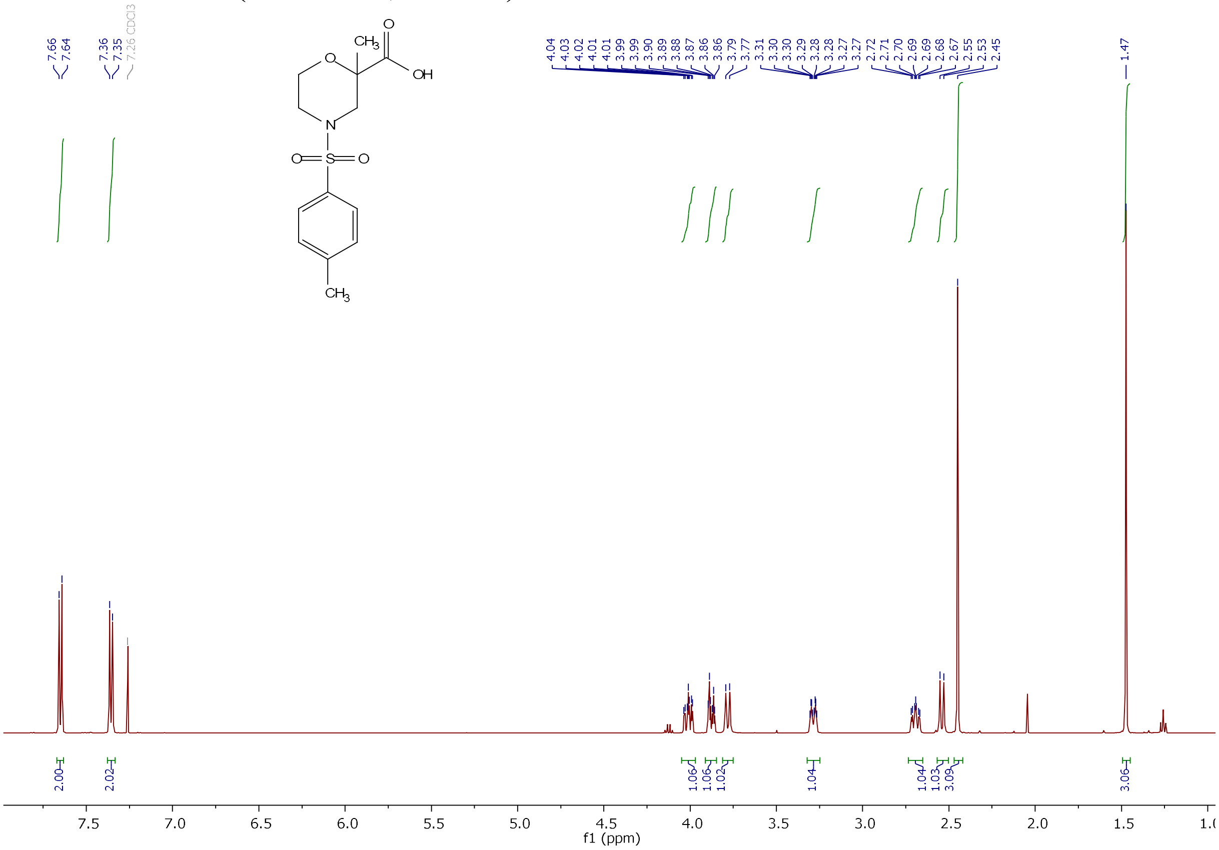

$^{13}C\{^{1}H\}$ NMR of **12** (126 MHz, $CDCl_3$)

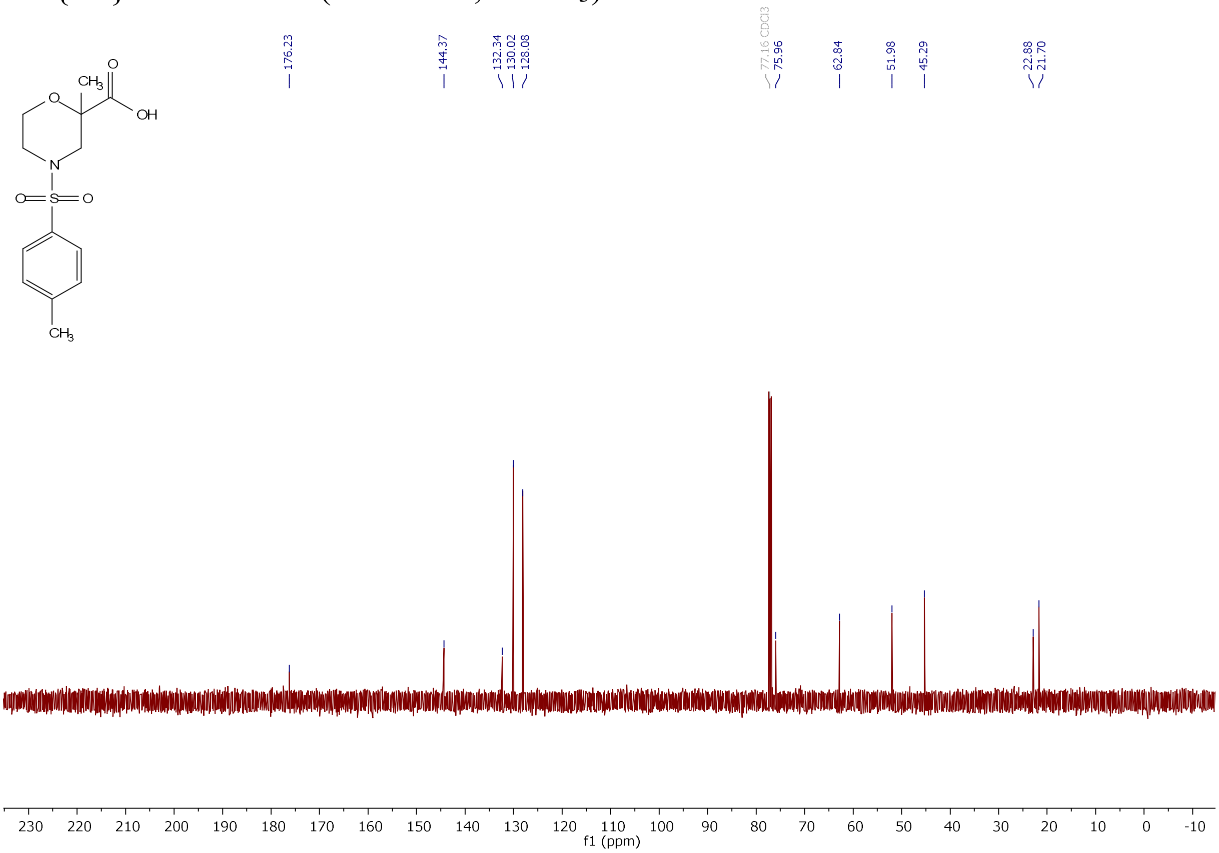

^{1}H NMR of **14** (500 MHz, $CDCl_3$)

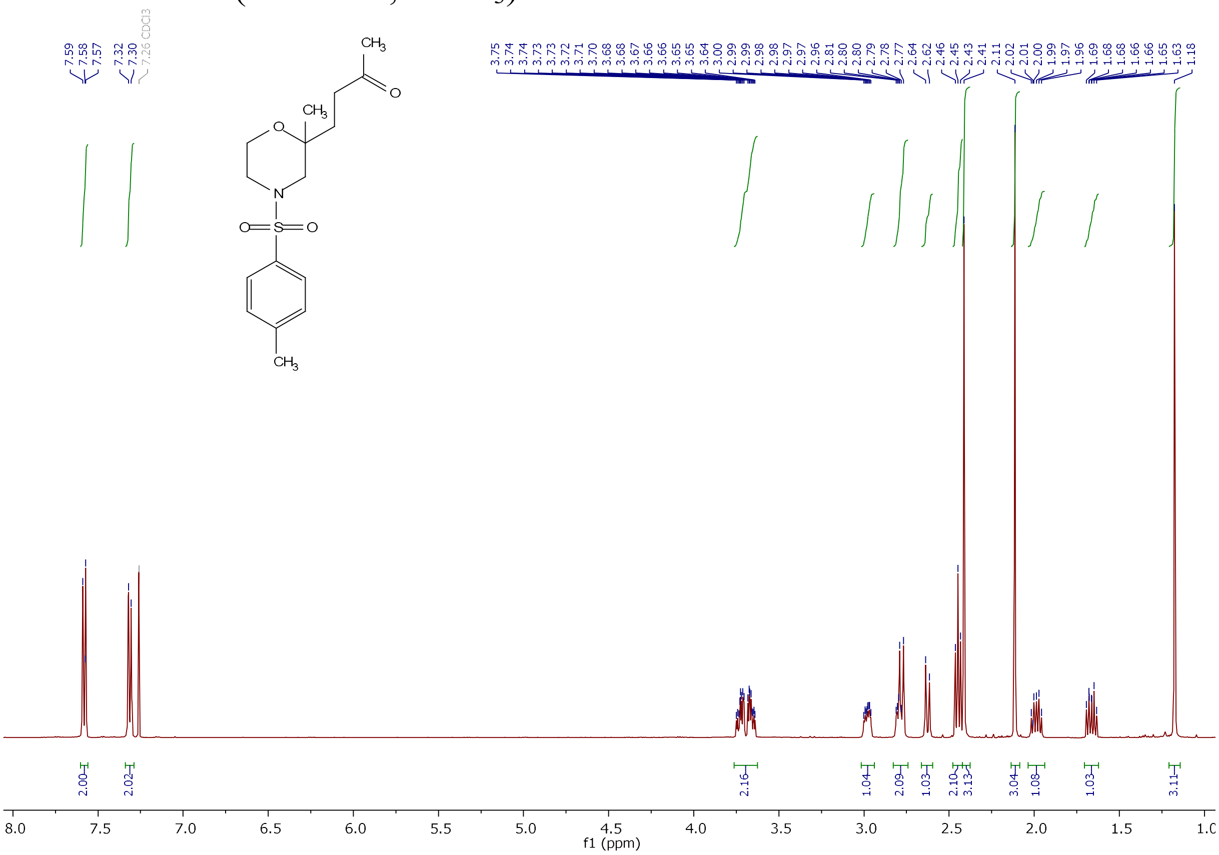

^{13}C{^{1}H} NMR of **14** (126 MHz, $CDCl_3$)

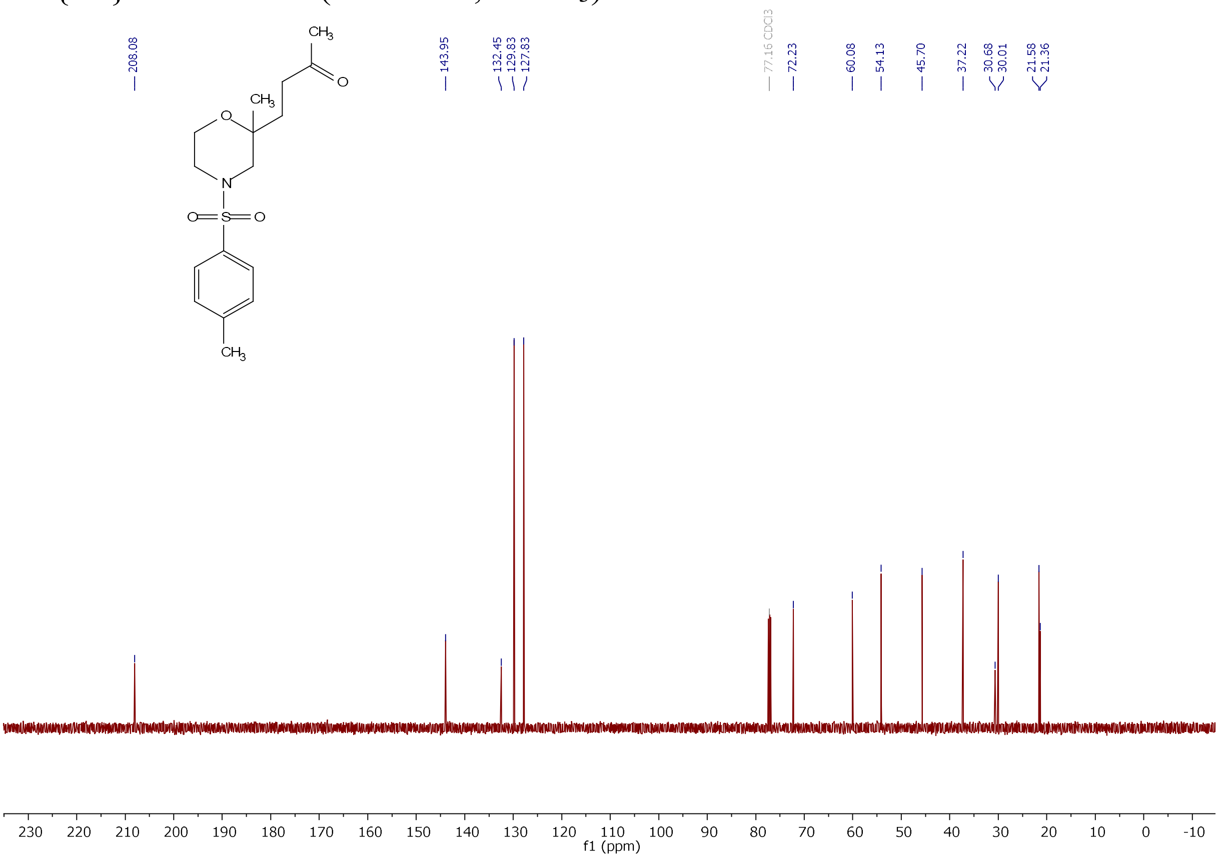

^{1}H NMR of **15’** (500 MHz, $CDCl_3$)

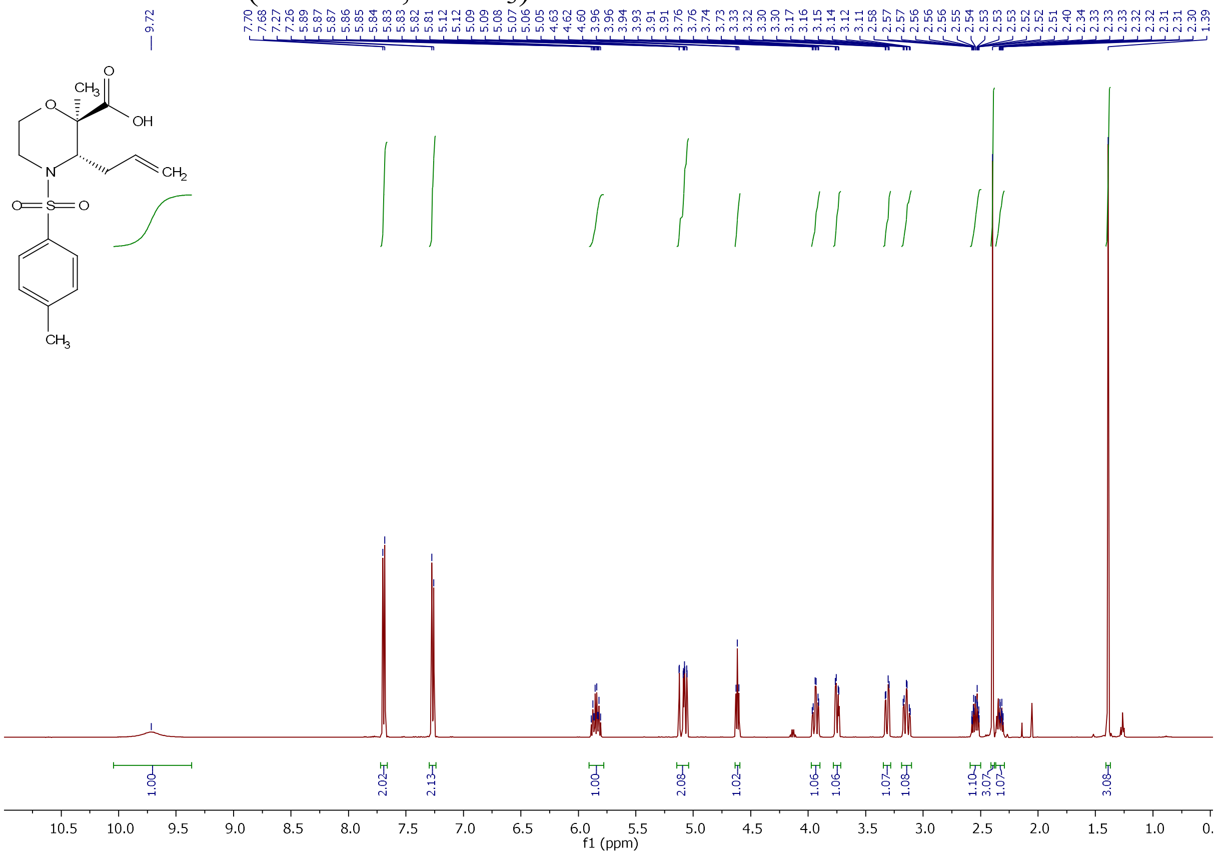

^{13}C{^{1}H} NMR of **15’** (126 MHz, $CDCl_3$)

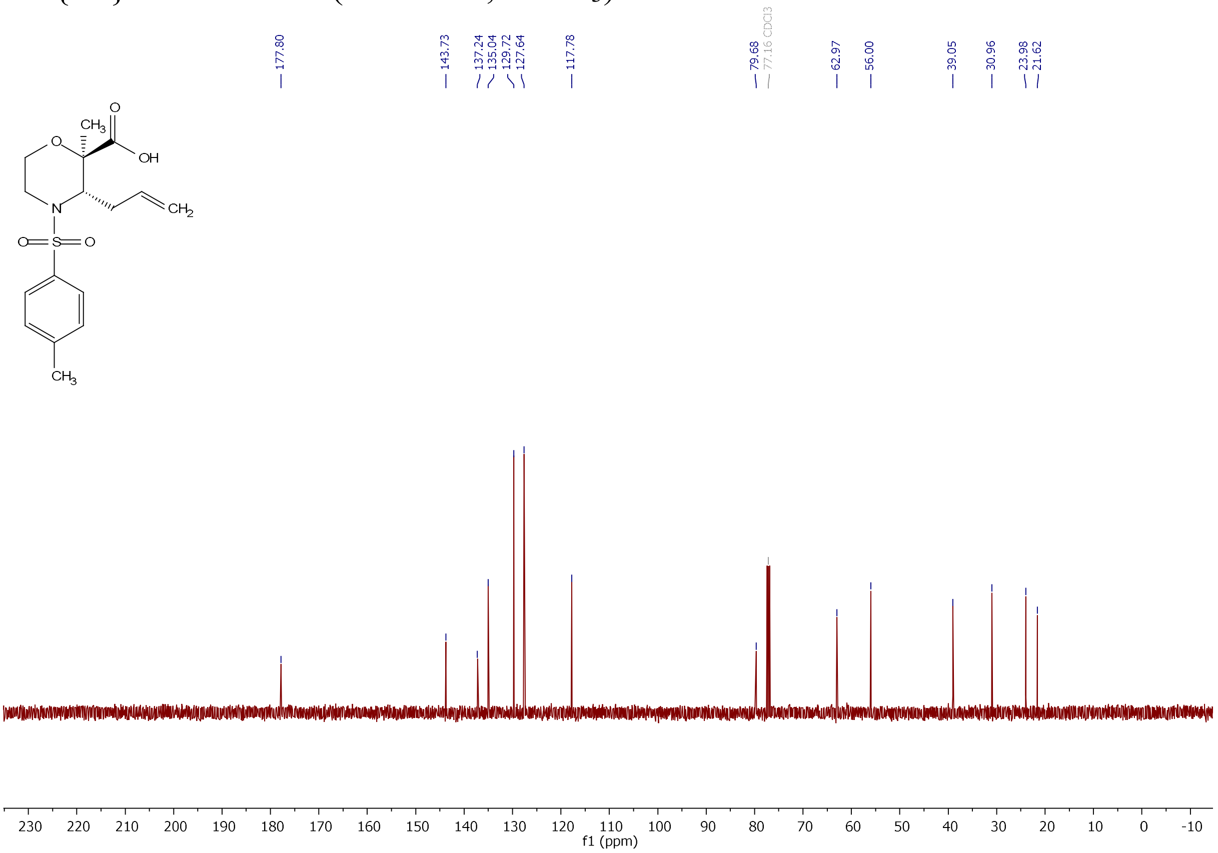

Key NOESY correlations of compound **15’**

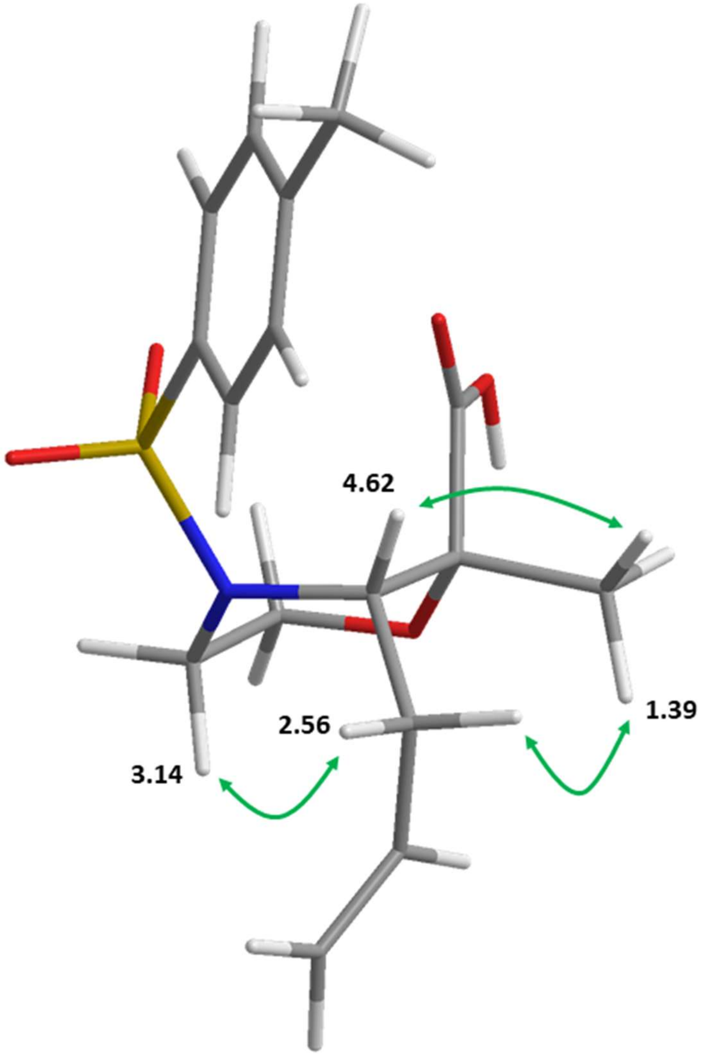

^{1}H NMR of **15”** (500 MHz, $CDCl_3$)

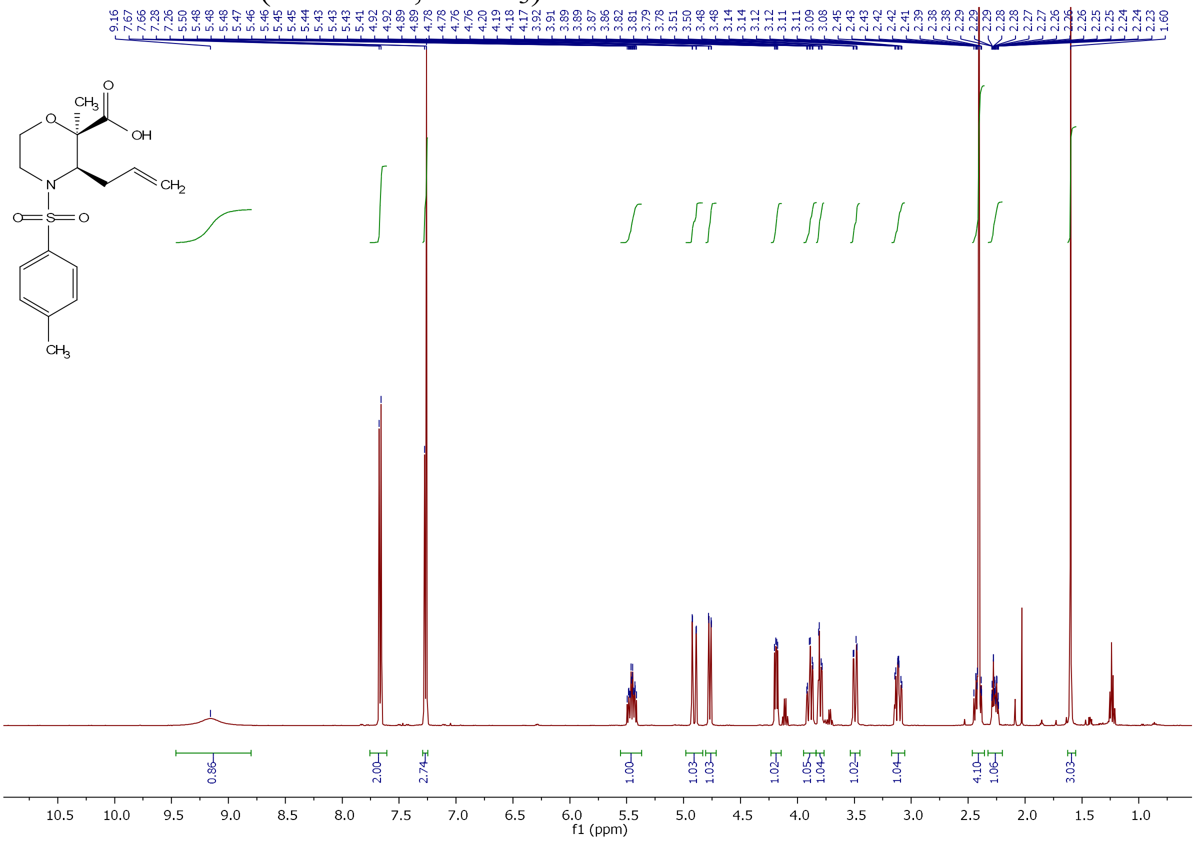

^{13}C{^{1}H} NMR of **15”** (126 MHz, $CDCl_3$)

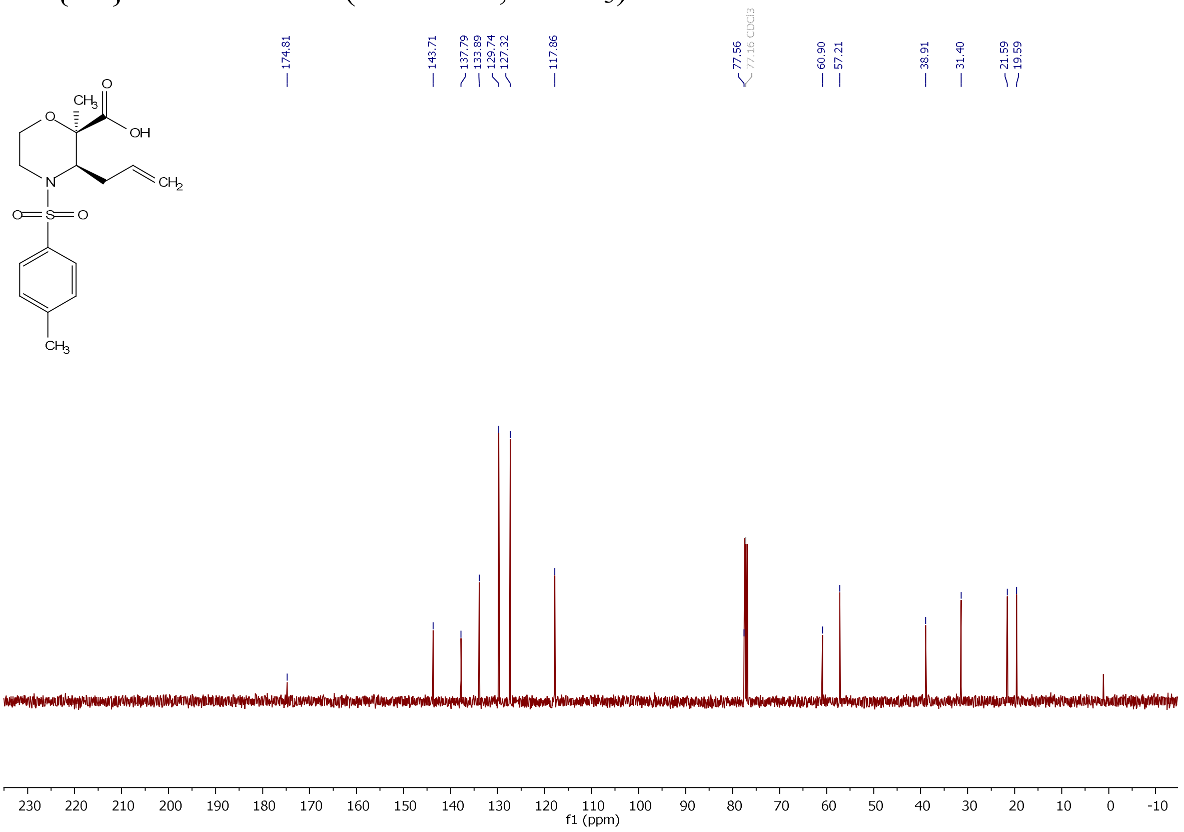

Key NOESY correlations of compound **15’’**

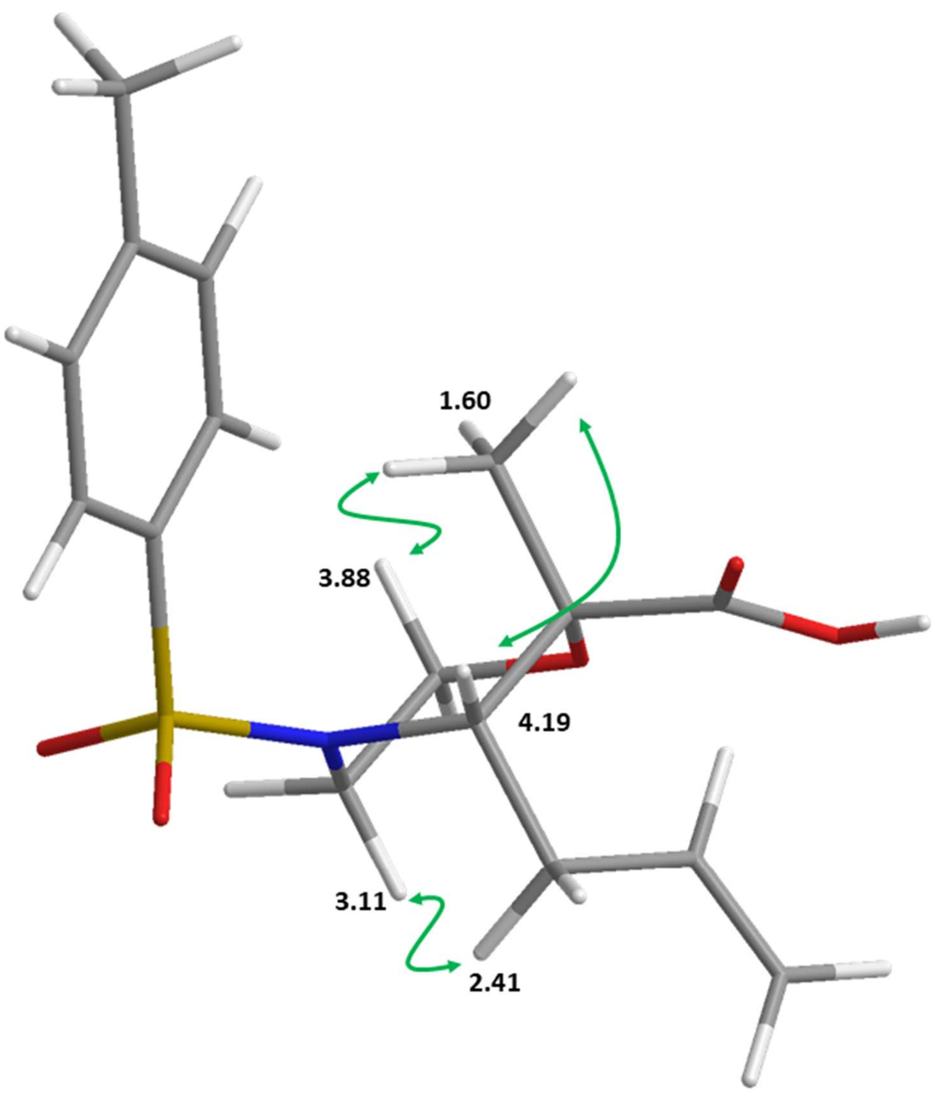

^{1}H NMR of **16** (500 MHz, $CDCl_3$)

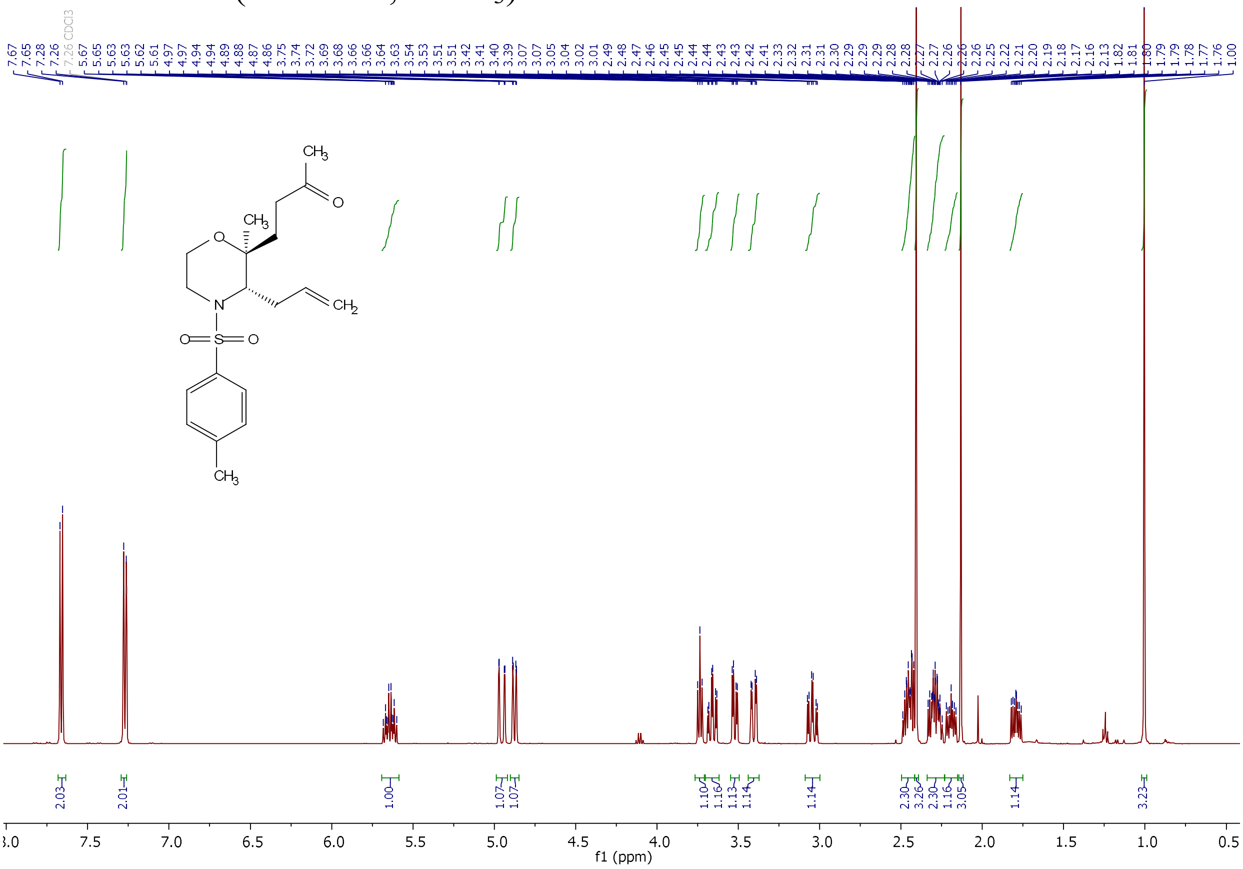

$^{13}C\{^{1}H\}$ NMR of **16** (126 MHz, $CDCl_3$)

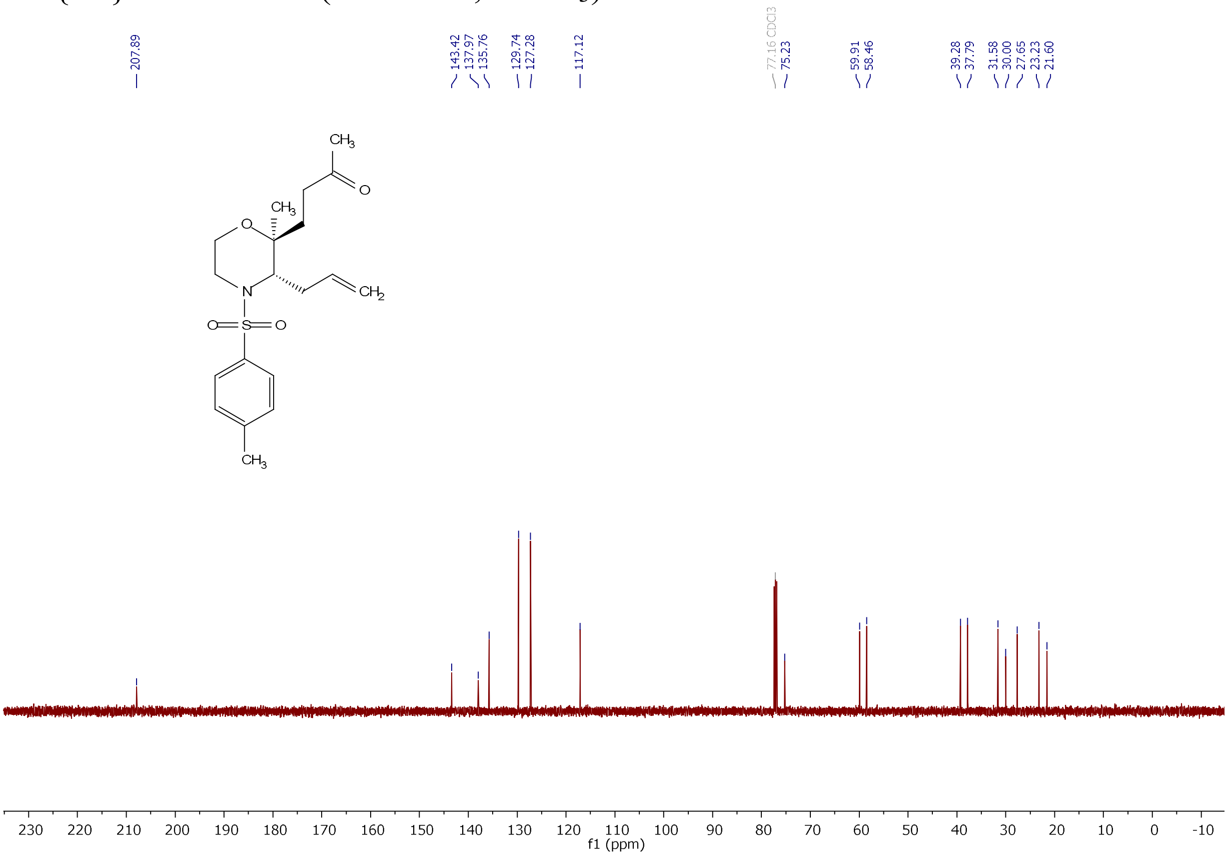

Key NOESY correlations of compound **16**

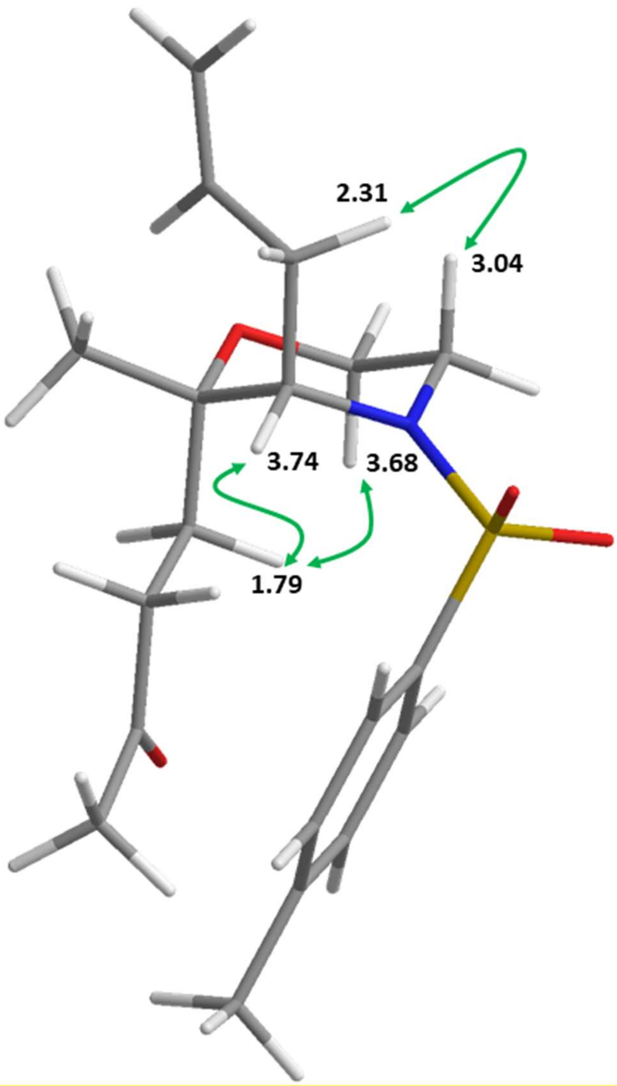

X-ray data

Experimental X-ray diffraction data are available from the Cambridge Crystallographic Data Centre on request quoting the deposition numbers CCDC 2201968 for *tert*-butyl 3-hydroxy-2-methyl-4-tosylmorpholine-2-carboxylate (**3c**) and CCDC 2201969 for methyl 3-cyano-2-methyl-4-tosylmorpholine-2-carboxylate (**7**).

***tert*-Butyl 3-hydroxy-2-methyl-4-tosylmorpholine-2-carboxylate (3c)**
Sample preparation: The racemic *tert*-butyl 3-hydroxy-2-methyl-4-tosylmorpholine-2-carboxylate (**3c**) was crystallized by slow evaporation from ethyl acetate and hexane solvents.
Crystal data: $C_{17}H_{25}NO_6S$, *Fwt.*: 371.44, colorless, chunk, size: 0.55 x 0.30 x 0.20 mm, monoclinic, space group $P\ 2_1$, a = 9.3497(4)Å, b = 10.6951(4)Å, c = 9.4311(4)Å, α = 90°, β = 97.321(7)°, γ = 90°, V = 935.38(7)Å^3, T = 294(2)K, Z= 2, F(000) = 396, D_x = 1.319 Mg/m^3, μ 0.205mm^{-1}.
A crystal of *tert*-butyl 3-hydroxy-2-methyl-4-tosylmorpholine-2-carboxylate (**3c**) was mounted on a fiber. Cell parameters were determined by least-squares using 47213 (3.285 ≤ θ ≤ 27.4998°) reflections.
Intensity data were collected on a Rigaku RAXIS-RAPID II diffractometer (monochromator; Mo-$K\alpha$ radiation, λ = 0.71075Å) at 293(2) K in the range 3.285 ≤ θ ≤ 27.476[1]. A total of 54348 reflections were collected of which 4278 were unique [R(int) = 0.0316, $R(\sigma)$ = 0.0194]; intensities of 3984 reflections were greater than 2σ(I). Completeness to θ = 0.997.
A numerical absorption correction was applied to the data (the minimum and maximum transmission factors were 0.978966 and 0.988504).
The structure was solved by direct methods[2] (and subsequent difference syntheses).
Anisotropic full-matrix least-squares refinement[3] on F^2 for all non-hydrogen atoms yielded R_1 = 0.0340 and wR^2 = 0.0742for 1332 [I>2σ(I)] and R_1 = 0.0392 and wR^2 = 0.0760 for all (4278) intensity data, (number of parameters = 232, goodness-of-fit = 1.061, the maximum and mean shift/esd is 0.000 and 0.000). The absolute structure parameter is 0.031(8). (Friedel coverage: 0.898, Friedel fraction max.: 0.998, Friedel fraction full: 0.999).
The maximum and minimum residual electron density in the final difference map was 0.26 and -0.26e.Å^{-3}.
The weighting scheme applied was $w = 1/[\sigma^2(F_o^2)+(0.04900.0000P)^2+0.0000P]$ where $P = (F_o^2+2F_c^2)/3$.
Hydrogen atomic positions were calculated from assumed geometries. Hydrogen atoms were included in structure factor calculations, but they were not refined. The isotropic displacement parameters of the hydrogen atoms were approximated from the U(eq) value of the atom they were bonded to.

Methyl 3-cyano-2-methyl-4-tosylmorpholine-2-carboxylate (7)
Sample preparation: The racemic methyl 3-cyano-2-methyl-4-tosylmorpholine-2-carboxylate (**7**) was crystallized by slow evaporation from ethyl acetate and hexane solvents.
Crystal data: $C_{15}H_{18}N_2O_5S$, *Fwt.*: 338.37, colorless, needle, size: 0.50 x 0.15 x 0.15 mm, triclinic, space group P -1, a = 7.0521(15)Å, b = 9.422(2)Å, c = 12.625(3)Å, α = 90.213(6)°, β = 104.418(7)°, γ = 97.088(7)°, V = 805.7(3)Å^3, T = 143(2)K, Z= 2, F(000) = 356, D_x = 1.395 Mg/m^3, μ 0.228mm^{-1}.
A crystal of methyl 3-cyano-2-methyl-4-tosylmorpholine-2-carboxylate (**7**) was mounted on a fiber. Cell parameters were determined by least-squares using 15378 (3.005 ≤ θ ≤ 27.63°) reflections.
Intensity data were collected on a Rigaku RAXIS-RAPID II diffractometer (monochromator;

Mo-$K\alpha$ radiation, λ = 0.71075Å) at 143(2) K in the range 3.007 ≤ θ ≤ 23.257[1]. A total of 14988 reflections were collected of which 14988 were unique [R(int) = 0.0979, $R(\sigma)$ = 0.1396]; intensities of 9562 reflections were greater than 2σ(I). Completeness to θ = 0.998.
A numerical absorption correction was applied to the data (the minimum and maximum transmission factors were 0.984457 and 0.995451).
The structure was solved by direct methods[2] (and subsequent difference syntheses).
Anisotropic full-matrix least-squares refinement[3] on F^2 for all non-hydrogen atoms yielded R_1 = 0.0716 and wR^2 = 0.1541for 1332 [I>2σ(I)] and R_1 = 0.1176 and wR^2 = 0.1719 for all (14988) intensity data, (number of parameters = 212, goodness-of-fit = 1.011, the maximum and mean shift/esd is 0.000 and 0.000).
The maximum and minimum residual electron density in the final difference map was 0.71 and -0.38e.Å^{-3}.
The weighting scheme applied was $w = 1/[\sigma^2(F_o^2)+(0.06220.0000P)^2+0.0000P]$ where $P = (F_o^2+2F_c^2)/3$.
Hydrogen atomic positions were calculated from assumed geometries. Hydrogen atoms were included in structure factor calculations but they were not refined. The isotropic displacement parameters of the hydrogen atoms were approximated from the U(eq) value of the atom they were bonded to.

Table S2. Summary of crystallographic data, data collections, structure determination and refinement for *tert*-butyl 3-hydroxy-2-methyl-4-tosylmorpholine-2-carboxylate and methyl 3-cyano-2-methyl-4-tosylmorpholine-2-carboxylate

Number	**3c**	**7**
CCDC	2201968	2201969
Empirical formula	$C_{17}H_{25}NO_6S$	$C_{15}H_{18}N_2O_5S$
Formula weight	371.44	338.37
Temperature	294(2)	143(2)
Radiation and wavelength	Mo-Kα,<br>λ =0.71075Å	Mo-Kα,<br>λ =0.71075Å
Crystal system	monoclinic	triclinic
Space group	$P\,2_1$	P -1
Unit cell dimensions		
a (Å)	9.3497(4)	7.0521(15)
b(Å)	10.6951(4)	9.422(2)
c(Å)	9.4311(4)	12.625(3)
α (°)	90	90.213(6)
β (°)	97.321(7)	104.418(7)
γ (°)	90	97.088(7)
Volume (Å^3)	935(1)	806(1)
Z, Z'	2, 1	2, 1
Density (calculated) (Mg/m^3)	1.319	1.395
Absorption coefficient, μ (mm^{-1})	0.205	0.228
F(000)	396	356
Crystal color, description	colorless, chunk	colorless, needle
Crystal size (mm)	0.55 x 0.30 x 0.20	0.50 x 0.15 x 0.15

Absorption correction	numerical	numerical
Max. and min. transmission	0.978966 and 0.988504	0.984457 and 0.995451
θ–range for data collection	3.285 ≤ θ ≤ 27.476°	3.007 ≤ θ ≤ 23.257°
Index ranges	-12 ≤ *h* ≤ 12;	-7 ≤ *h* ≤ 7;
	-13≤ *k* ≤13;	-10≤ *k* ≤10;
	-12 ≤ *l* ≤ 12	-14 ≤ *l* ≤ 14
Reflections collected	54348	14988
Completeness to 2θ	0.997	0.998
Independent reflections	4278	14988
	[*R*(int) =0.0316]	[*R*(int) =0.0979]
Reflections $I>2\sigma(I)$	3984	9562
Data / restraints / parameters	4278 /1 /232	14988 /0 /212
Goodness-of-fit on *F*2	1.061	1.011
Final *R* indices [$I>2\sigma(I)$]	R_1 =0.0340,	R_1 =0.0716,
	wR^2 =0.0742	wR^2 =0.1541
R indices (all data)	R_1 =0.0392,	R_1 =0.1176,
	wR^2 =0.0760	wR^2 =0.1719
Max. and mean shift/esd	0.000;0.000	0.000;0.000
Largest diff. peak and hole	0.26;-0.26 e.Å^{-3}	0.71;-0.38 e.Å^{-3}
Absolute structure parameter		*centrosymmetric space group*
Flack (x)	0.031(8)	-
Parsonz (z)	0.036(12)	-
Hooft (y)	0.023(7)	-

References:

[1]CrystalClear SM 1.4.0 (Rigaku/MSC Inc., 2008).
[2]Numerical Absorption Correction: T. Higashi, (1998), rev. 2002. (Rigaku/MSC Inc.).
[3] G. M. Sheldrick, *Acta Cryst.* 2015, **C71**, 3-8.
